# An updated tribal classification of Lamiaceae based on plastome phylogenomics

**DOI:** 10.1186/s12915-020-00931-z

**Published:** 2021-01-08

**Authors:** Fei Zhao, Ya-Ping Chen, Yasaman Salmaki, Bryan T. Drew, Trevor C. Wilson, Anne-Cathrine Scheen, Ferhat Celep, Christian Bräuchler, Mika Bendiksby, Qiang Wang, Dao-Zhang Min, Hua Peng, Richard G. Olmstead, Bo Li, Chun-Lei Xiang

**Affiliations:** 1 CAS Key Laboratory for Plant Diversity and Biogeography of East Asia, Kunming Institute of Botany, Chinese Academy of Sciences, Kunming, 650201 China; 2 Center of Excellence in Phylogeny of Living Organisms, Department of Plant Science, College of Science, University of Tehran, P.O. Box 14155-6455, Tehran, Iran; 3 Department of Biology, University of Nebraska at Kearney, Kearney, NE 68849 USA; 4 National Herbarium of New South Wales, Australian Institute of Botanical Science, Royal Botanic Gardens & Domain Trust, Sydney, Australia; 5 Museum of Archaeology, University of Stavanger, NO-4036 Stavanger, Norway; 6 Department of Biology, Faculty of Arts and Sciences, Kırıkkale University, Kırıkkale, Turkey; 7 Department of Botany, University of Wisconsin–Madison, Madison, WI 53706 USA; 8 Department of Botany, Natural History Museum Vienna, Burgring 7, 1010 Wien, Austria; 9 NTNU University Museum, Norwegian University of Science and Technology, 7491 Trondheim, Norway; 10 Natural History Museum, University of Oslo, Oslo, Norway; 11 State Key Laboratory of Systematic & Evolutionary Botany, Institute of Botany, Chinense Academy of Sciences, Xiangshan, Beijing, 100093 China; 12 Research Centre of Ecological Sciences, College of Agronomy, Jiangxi Agricultural University, Nanchang, 330045 China; 13 Department of Biology, University of Washington, Seattle, WA USA

**Keywords:** Lamiaceae, Lamioideae, Mints, Phylogenomics, Tribal relationships

## Abstract

**Background:**

A robust molecular phylogeny is fundamental for developing a stable classification and providing a solid framework to understand patterns of diversification, historical biogeography, and character evolution. As the sixth largest angiosperm family, Lamiaceae, or the mint family, consitutes a major source of aromatic oil, wood, ornamentals, and culinary and medicinal herbs, making it an exceptionally important group ecologically, ethnobotanically, and floristically. The lack of a reliable phylogenetic framework for this family has thus far hindered broad-scale biogeographic studies and our comprehension of diversification. Although significant progress has been made towards clarifying Lamiaceae relationships during the past three decades, the resolution of a phylogenetic backbone at the tribal level has remained one of the greatest challenges due to limited availability of genetic data.

**Results:**

We performed phylogenetic analyses of Lamiaceae to infer relationships at the tribal level using 79 protein-coding plastid genes from 175 accessions representing 170 taxa, 79 genera, and all 12 subfamilies. Both maximum likelihood and Bayesian analyses yielded a more robust phylogenetic hypothesis relative to previous studies and supported the monophyly of all 12 subfamilies, and a classification for 22 tribes, three of which are newly recognized in this study. As a consequence, we propose an updated phylogenetically informed tribal classification for Lamiaceae that is supplemented with a detailed summary of taxonomic history, generic and species diversity, morphology, synapomorphies, and distribution for each subfamily and tribe.

**Conclusions:**

Increased taxon sampling conjoined with phylogenetic analyses based on plastome sequences has provided robust support at both deep and shallow nodes and offers new insights into the phylogenetic relationships among tribes and subfamilies of Lamiaceae. This robust phylogenetic backbone of Lamiaceae will serve as a framework for future studies on mint classification, biogeography, character evolution, and diversification.

**Graphical abstract:**

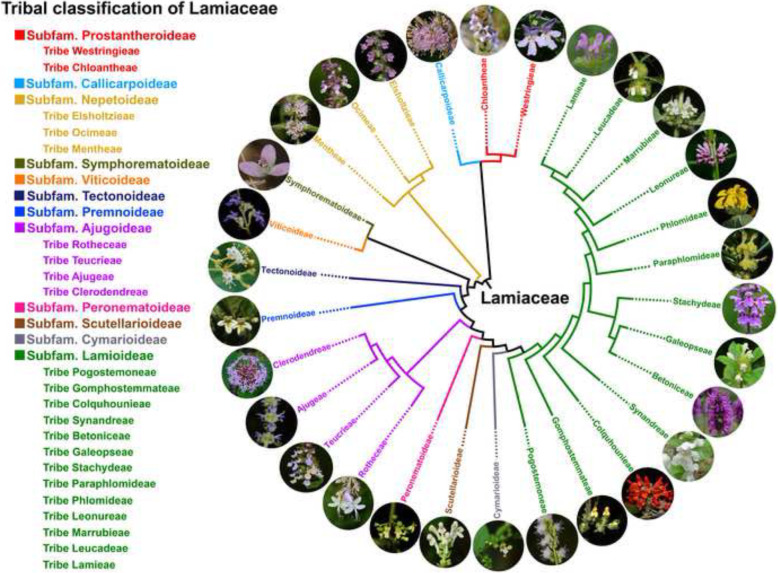

**Supplementary Information:**

The online version contains supplementary material available at 10.1186/s12915-020-00931-z.

## Background

Lamiaceae, generally known as the mint family, have long been known for their aromatic oils, which have played an undeniably significant role within culinary, medicinal, and horticultural aspects of human history. Species of Lamiaceae are of wide economic importance as sources of wood (e.g., *Tectona grandis* L. f.), landscape ornamentals (e.g., scarlet sage [*Salvia splendens* Sellow ex Wied-Neuw.]), cosmetics (e.g., lavender [*Lavandula angustifolia* Mill.]), culinary herbs (e.g., basil [*Ocimum basilicum* L.], oregano [*Origanum vulgare* L.], thyme [*Thymus vulgaris* L.]), and medicinal herbs (e.g., Korean mint [*Agastache rugosa* (Fisch. & C.A. Mey.) Kuntze], peppermint [*Mentha* × *piperita* L.]). Despite the recognition of this family (Lamiaceae s.s.) from advances in systematics and taxonomy of the late twentieth century, the family has historically been considered a “natural” group based on a combination of readily recognizable features such as an herbaceous habit, quadrangular stems, opposite phyllotaxy, bilabiate flowers, a gynobasic style, and four nutlets. However, morphological and molecular phylogenetic studies in the past three decades have significantly changed the concept of the family, and an expanded Lamiaceae (Lamiaceae s.l.) is now widely accepted. As currently circumscribed, Lamiaceae comprise more than 230 genera and over 7000 species, making it the sixth largest angiosperm family and the largest family in the order Lamiales [1–3]. Although unequivocally shown to be members of the family, inclusion of some disparate groups such as *Vitex* L. (originally placed in Verbenaceae because they were trees with fleshy fruits) has challenged the earlier concepts of the family.

Early infrafamilial classifications within Lamiaceae were predominately based on the treatment of Bentham [4], who divided the family into eight tribes. Briquet [5], for example, followed the division of Bentham [4], but raised some of the tribes to subfamilial rank and merged four tribes into the single large subfamily Lamioideae. Erdtman [6], however, recognized only two subfamilies based on palynological distinctions, viz., Lamioideae (with tricolpate pollen shed at the two-celled stage) and Nepetoideae (with hexacolpate pollen shed at the three-celled stage). Combining the classifications of Briquet [5] and Erdtman [6], Wunderlich [7] recognized six subfamilies within Lamiaceae, rejecting Lamioideae as circumscribed by Briquet [5] and accepting a subfamily Nepetoideae close to that of Erdtman [6]. Cantino and Sanders [8] revealed that Nepetoideae sensu Erdtman [6] is monophyletic with several synapomorphies, whereas no synapomorphy was found for Lamioideae sensu Erdtman [6].

The mint family has long been thought to have evolved from Verbenaceae-like ancestors, and these two families were considered separate largely based upon gynoecial structure. Although a deeply four-lobed ovary with a gynobasic style is typical for most traditionally recognized Lamiaceae (i.e. Lamiaceae s.s.), and an unlobed ovary with a terminal style is typical of most Verbenaceae, there exists in both families a continuum in extent of lobing and separation of fruits into single seeded units [9]. Noting this, Cantino [9, 10] carried out a cladistic analysis of the Lamiaceae s.s. and the Verbenaceae s.l. based on 85 morphological and anatomical characters, which provided support to reject that the Lamiaceae s.s. was monophyletic, demonstrating several clades of the Verbenaceae s.l. recovered among clades of the Lamiaceae s.s. Based on these results, Cantino et al. [11] published a list of subfamilies and genera of the Lamiaceae s.l. that had been proposed earlier by Junell [12]. This incorporated the transfer of the cymose subfamilies Caryopteridoideae, Chloanthoideae, Viticoideae, Symphorematoideae, and tribe Monochileae to the expanded Lamiaceae, rendering the Verbenaceae s.s. as only the subfamily Verbenoideae. Verbenaceae s.s. can be recognized by having racemose inflorescences, tricolporate pollen, and ovules attached to the carpel margins, while the Lamiaceae s.l. generally possess thyrsoid inflorescences, colpate pollen, and ovules attached to the sides of the false septa of ovary [13]. Moreover, the Verbenaceae s.s. have thickened stigma lobes with conspicuous stigmatic tissue, hypocrateriform corollas with included stamens, and usually terete stems, whereas in the Lamiaceae s.l., stigma lobes are slender with inconspicuous stigmatic tissue, corollas that are rarely hypocrateriform, and stems are typically quadrangular. Since Cantino et al. [11], the expanded concept of the Lamiaceae s.l. has been consistently supported as monophyletic by molecular phylogenetic studies [14–20] and is widely accepted in various classifications [1, 3]. We acknowledge these results and use the names Lamiaceae and Verbenaceae in their contemporary circumscription. Though today both Lamiaceae and Verbenaceae are placed within “core Lamiales” of the asterids, they have unexpectedly not been recovered as sister taxa despite their morphological similarities: Lamiaceae belong to a clade that includes Mazaceae, Phrymaceae, Wightiaceae, Paulowniaceae, and Orobanchaceae, whereas Verbenaceae are recovered as sister to Thomandersiaceae [18, 21, 22].

Following Cantino et al. [11], Harley et al. [1] published a global, genus-level taxonomic conspectus of Lamiaceae. Except for the ten genera *Acrymia* Prain, *Callicarpa* L., *Cymaria* Benth., *Garrettia* H.R. Fletch., *Holocheila* (Kudô) S. Chow, *Hymenopyramis* Wall. ex Griff., *Ombrocharis* Hand.-Mazz., *Peronema* Jack, *Petraeovitex* Oliv., and *Tectona* L. that were treated as *incertae sedis*, the remaining 226 genera were assigned to seven subfamilies: Ajugoideae, Lamioideae, Nepetoideae, Prostantheroideae, Scutellarioideae, Symphorematoideae, and Viticoideae [1]. Since the publication of this classification [1], numerous molecular phylogenetic studies have been carried out to explore the relationships at the subfamilial [19], tribal [23–33], or generic [34–50] level. However, relationships among four subfamilies (Nepetoideae, Tectonoideae, Premnoideae, and Ajugoideae) remain unresolved and those among some tribes were also unclear in those studies.

In terms of taxon number, the most comprehensively sampled phylogenetic study of Lamiaceae was conducted by Li et al. [19] using an ingroup sampling of 288 species from 191 genera and employing five plastid DNA regions (*matK*, *ndhF*, *rbcL*, *rps16*, and *trnL*-*trnF*). The backbone of this phylogeny was comprised of 12 clades, all provided with high branch support, and seven of which corresponded to a portion of the Viticoideae and six of the previously recognized subfamilies of Harley et al. [1]. The other five clades consisted of previously *incertae sedis* genera and were each provided subfamilial rank as the Cymarioideae (including *Acrymia* and *Cymaria*), Peronematoideae (including *Hymenopyramis*, *Petraeovitex*, *Peronema*, and *Garrettia*), Premnoideae (including *Premna* L., *Gmelina* L., and *Cornutia* L.), Callicarpoideae (including *Callicarpa*), and Tectonoideae (including *Tectona*) [19, 51].

Despite the improved resolution in our understanding of Lamiaceae and its subfamilies, the work by Li et al. [19] was not able to clarify relationships among Nepetoideae, Tectonoideae, Premnoideae, and Ajugoideae, nor were they able to provide resolution to understand the tribal classification within some subfamilies (viz. Lamioideae). While recent phylogenetic analyses have greatly improved our understanding of the major lineages and classifications of Lamioideae [52, 53], the tribal membership of *Betonica* L., *Colquhounia* Wall., *Galeopsis* L., *Metastachydium* Airy Shaw ex C.Y. Wu & H.W. Li, *Paralamium* Dunn., and *Roylea* Wall. ex Benth. remains unclear [2, 53]. Furthermore, Xiang et al. [54] identified four major clades within the Ajugoideae, but did not propose a formal tribal classification. The uncertain relationships among and within these subfamilies have hindered the further study of character evolution and diversification patterns within Lamiaceae.

Next-generation sequencing (NGS) provides a significantly larger amount of DNA sequence data than has been previously available for phylogenetic studies within angiosperms [55]. While the use of complete plastome sequences is not a panacea [56], it has successfully resolved previously intractable phylogenetic problems within flowering plants at multiple taxonomic levels [57–65]. Concordantly, recent phylogenomic studies based on plastome sequences have provided new insight into both generic and species-level relationships within Scutellarioideae [66] and *Salvia* [67], respectively. In order to resolve the remaining ambiguities at the tribal and subfamilial level, we sequenced and analyzed the complete plastome for 175 representative taxa from all currently recognized tribes in the 12 subfamilies of Lamiaceae. The focus of this study was to (1) improve the resolution of the phylogenetic backbone of Lamiaceae, (2) modify the tribal classification of Lamiaceae based on our results, and (3) provide a summary of the recent phylogenetic and taxonomic progress achieved for each subfamily and tribe.

## Results

### Characteristic of plastome features and datasets

Our sequencing generated between 13,829,468 (*Siphocranion flavidum* Y.P. Chen & C.L. Xiang) and 81,265,290 (*Chloanthes coccinea* Bartl.) clean reads from the 50 newly sequenced species, with the mean base coverage ranging from 110× (*Congea tomentosa* Roxb.) to 3104× (*Lamium amplexicaule* L.) estimated by the GetOrganelle pipeline [68]. Since we failed to assemble the complete plastome of *Callicarpa americana* L., the average base coverage for this species is unavailable (noted as “NA” in Table 1). Statistics about the assemblies for each newly sequenced species are provided in Table 1.

**Table 1:** Newly sampled species in this study (*NA* data unavailable)

Systematic assignment		Species	Locality	Clean reads	Mean coverage of base (x)	GenBank accession numbers
Phrymaceae (outgroup)		*Mimulus* sp.	The United States Botanic Garden (USBG), United States	19,584,540	478	MT473772
Ajugoideae	Ajugeae	*Caryopteris forrestii* Diels	Lijiang, Yunnan, China	67,295,160	485	MT473742
Ajugoideae	Teucrieae	*Schnabelia oligophylla* Hand.-Mazz.	Kunming, Yunnan, China	67,359,376	726	MT473777
Ajugoideae	Clerodendreae	*Clerodendrum japonicum* (Thunb.) Sweet	Kunming, Yunnan, China	69,357,954	854	MT473745
Ajugoideae	Clerodendreae	*Clerodendrum trichotomum* Thunb.	Huairou, Beijing, China	69,621,568	536	MT473746
Ajugoideae	Rotheceae	*Rotheca serrata* (L.) Steane & Mabb.	Kunming, Yunnan, China	69,698,896	328	MT473776
Callicarpioideae	–	*Callicarpa americana* L.	Gainesville, Florida, United States	69,222,992	NA	--
Callicarpioideae	–	*Callicarpa arborea* Roxb.	Kunming, Yunnan, China	70,066,596	341	MT473738
Callicarpioideae	–	*Callicarpa brevipes* (Benth.) Hance	Guangzhou, Guangdong, China	68,119,222	383	MT473739
Callicarpioideae	–	*Callicarpa macrophylla* Vahl	Kunming, Yunnan, China	69,104,110	499	MT473740
Callicarpioideae	–	*Callicarpa peichieniana* Chun & S.L. Chen ex H. Ma & W.B. Yu	Guangzhou, Guangdong, China	68,759,068	215	MT473741
Cymarioideae	–	*Cymaria dichotoma* Benth.	Changjiang, Hainan, China	68,070,464	1189	MT473753
Lamioideae	Paraphlomideae	*Paraphlomis javanica* (Blume) Prain	Kunming, Yunnan, China	66,797,022	239	MT473773
Lamioideae	Gomphostemmateae	*Gomphostemma lucidum* Wall. ex Benth.	Changjiang, Hainan, China	66,781,246	274	MT473764
Lamioideae	Gomphostemmateae	*Chelonopsis souliei* (Bonati) Merr.	Litang, Sichuan, China	67,646,436	572	MT473743
Lamioideae	Colquhounieae	*Colquhounia coccinea* Wall.	Kunming, Yunnan, China	66,842,836	171	MT473749
Lamioideae	Colquhounieae	*Colquhounia seguinii* Vaniot	Kunming, Yunnan, China	66,760,344	337	MT473750
Lamioideae	Colquhounieae	*Colquhounia vestita* Wall.	Cuona, Xizang, China	67,753,130	192	MT473751
Lamioideae	Lamieae	*Lamium amplexicaule* L.	Zuogong, Xizang, China	67,339,814	3104	MT473770
Lamioideae	Synandreae	*Macbridea alba* Chapm.	The United States Botanic Garden (USBG), United States	20,514,794	474	MT473771
Lamioideae	Stachydeae	*Galeopsis bifida* Boenn.	Deqin, Yunnan, China	67,442,714	500	MT473759
Nepetoideae	Elsholtzieae	*Elsholtzia densa* Benth.	Shangri-La, Yunnan, China	18,273,016	888	MT473757
Nepetoideae	Elsholtzieae	*Elsholtzia rugulosa* Hemsl.	Kunming, Yunnan, China	67,318,028	553	MT473758
Nepetoideae	Ocimeae	*Siphocranion flavidum* Y.P. Chen & C.L. Xiang	Malipo, Yunnan, China	13,829,468	436	MT473778
Nepetoideae	Ocimeae	*Siphocranion macranthum* (Hook. f.) C.Y. Wu	Nanchuan, Congqing, China	13,860,798	241	MT473779
Nepetoideae	Ocimeae	*Hanceola exserta* Y.Z. Sun ex C.Y. Wu	Hezhou, Guangxi, China	67,557,758	203	MT473765
Nepetoideae	Ocimeae	*Isodon amethystoides* (Benth.) H. Hara	Lin'an, Zhejiang, China	25,146,824	696	MT473767
Nepetoideae	Ocimeae	*Isodon lophanthoides* (Buch.-Ham. ex D. Don) H. Hara	Kunming, Yunnan, China	40,730,966	316	MT473768
Nepetoideae	Ocimeae	*Isodon ternifolius* (D. Don) Kudô	Longling, Yunnan, China	32,984,960	542	MT473769
Nepetoideae	Ocimeae	*Coleus xanthanthus* C.Y. Wu & Y.C. Huang	Mengla, Yunnan, China	25,669,120	821	MT473748
Nepetoideae	Menheae	*Dracocephalum taliense* Forrest	Heqing, Yunnan, China	68,863,176	446	MT473756
Nepetoideae	Menheae	*Clinopodium abyssinicum* (Benth.) Kuntze	Kabarnet, Baringo, Kenya	48,657,815	833	MT473747
Peronematoideae	–	*Garrettia siamensis* H.R. Fletcher	Mengla, Yunnan, China	69,566,486	1905	MT473760
Peronematoideae	–	*Hymenopyramis cana* Craib	Changjiang, Hainan, China	66,946,216	298	MT473766
Premnoideae	–	*Premna szemaoensis* C. P'ei	Kunming, Yunnan, China	69,409,616	477	MT473775
Premnoideae	–	*Premna vietnamensis* Bo Li	K'Bang, Gia Lai, Vietnam	80,675,070	460	MT473774
Premnoideae	–	*Gmelina arborea* Roxb. ex Sm.	Mengla, Yunnan, China	67,974,942	493	MT473761
Premnoideae	–	*Gmelina hainanensis* Oliv.	Kunming, Yunnan, China	67,354,640	1527	MT473762
Premnoideae	–	*Gmelina philippensis* Cham.	Mengla, Yunnan, China	69,953,046	479	MT473763
Prostantheroideae	Chloantheae	*Chloanthes coccinea* Bartl.	Australian National Botanic Gardens (ANBG), Australia	81,265,290	598	MT473744
Prostantheroideae	Chloantheae	*Dasymalla teckiana* (F. Muell.) B.J. Conn & Henwood	Australian National Botanic Gardens (ANBG), Australia	41,308,508	519	MT473754
Prostantheroideae	Chloantheae	*Dicrastylis parvifolia* F. Muell.	Australian National Botanic Gardens (ANBG), Australia	81,081,410	577	MT473755
Symphorematoideae	–	*Congea tomentosa* Roxb.	Mengla, Yunnan, China	40,494,132	110	MT473752
Symphorematoideae	–	*Sphenodesme mollis* Craib	Mengla, Yunnan, China	81,008,454	529	MT473780
Tectonoideae	–	*Tectona grandis* L. f.	Mengla, Yunnan, China	40,169,710	514	MT473781
Viticoideae	–	*Vitex glabrata* R. Br.	Mengla, Yunnan, China	70,126,282	722	MT473782
Viticoideae	–	*Vitex negundo* var. *cannabifolia* (Siebold & Zucc.) Hand.-Mazz.	Kunming, Yunnan, China	67,083,468	1387	MT473783
Viticoideae	–	*Vitex quinata* (Lour.) F.N. Williams	Mengla, Yunnan, China	69,282,366	828	MT473784
Viticoideae	–	*Vitex tripinnata* (Lour.) Merr.	Guangzhou, Guangdong, China	67,065,514	1404	MT473785
Viticoideae	–	*Vitex yunnanensis* W.W. Sm.	Luquan, Yunnan, China	70,217,642	395	MT473786

All plastomes exhibit a typical quadripartite structure of the large single-copy (LSC, 81,341–85,891 bp) and small single-copy (SSC, 9969–20,681 bp) regions, separated by a pair of inverted repeats (IR regions, 23,085–31,573 bp). The chloroplast genome maps are provided in Additional file 1 (Fig. S1). The GC content was evenly distributed, and the average GC content was 38.10% (Additional file 2: Table S1). All the newly sequenced and annotated plastomes in the present study were submitted to the National Center for Biotechnology Information (NCBI) database with accession numbers MT473738–MT473786 (Table 1).

The aligned length of the combined 79 protein-coding regions (CR) is 72,082 bp. Removal of ambiguous sites and single-taxon insertions results in an aligned length of 69,822 bp (CRM), of which 41,459 sites are constant (59.38%). The aligned regions and the excluded ambiguous sites of the individual loci are listed in Additional file 3 (Table S2), and properties of the five datasets are summarized in Table 2

**Table 2:** Data characteristics with models selected for each dataset used for phylogenetic study in the present study

Dataset	CRM	CR	CR12	CR3	dePCS
GC content	38.3%	38.3%	40.2%	34.5%	30.8%
Alignment sites (bp)	69,822	72,082	48,069	24,013	72,082
Constant sites (bp)	41,459	43,415	31,083	12,331	50,977
Parsimony-informative sites (bp)	29,945	20,185	11,561	8,624	14,473
Variable sites (bp)	28,363	28,667	16,986	11,682	21,105
Missing data	4.31%	4.31%	4.31%	4.31%	4.31%
Best-fit model	GTR+I+G	GTR+I+G	GTR+I+G	GTR+I+G	GTR+G

.

### Phylogenomic analyses

**Fig. 1.**
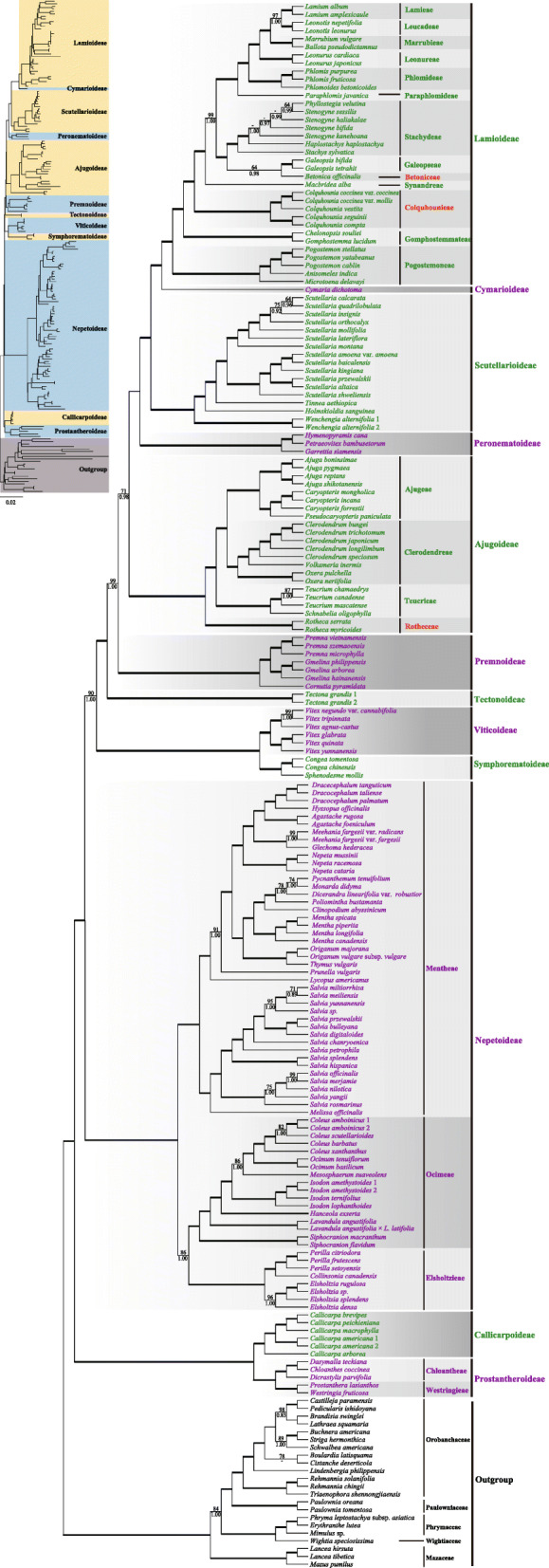
Maximum likelihood phylogeny of Lamiaceae based on combined 79 plastid coding regions dataset, with ambiguously aligned sites excluded. Maximum likelihood bootstrap support (MLBS) and Bayesian inference posterior probability (BIPP) are shown above and below the branches, respectively. Bold horizontal lines indicate clades with BIPP = 1.00) and MLBS = 100%. A “–” indicates MLBS values < 50% and BIPP < 0.8. Subfamilies and tribes recognized by Li et al. [19] and Li and Olmstead [51] are indicated by gray boxes, while new tribes proposed in this study were marked in red font

All analyses yielded an identical topology for the ingroup at the tribal level (Fig. 1; Additional files 4, 5, 6, 7: Figs. S2, S3, S4, S5), although the support is variable among different datasets. All 12 subfamilies were recovered and well-supported in all analyses (Fig. 1; Additional files 4, 5, 6, 7: Figs. S2, S3, S4, S5). The topology recovered by the combined dataset with the ambiguously aligned positions excluded (CRM) is presented as the primary tree (Fig. 1) for the following discussion of phylogenetic relationships.

Within Lamiaceae, two primary clades were recovered and subdivided as 12 clades corresponding to the 12 subfamilies (Fig. 1), with each subfamily being monophyletic (excepting Cymarioideae, which was represented by only one species). The first clade comprised the Prostantheroideae and Callicarpoideae (i.e., Calliprostantherina sensu Li et al. [19]), both with strong support (MLBS = 100%, BIPP = 1.00; Fig. 1; Additional files 4, 5, 6, 7: Figs. S2, S3, S4, S5, and all support values follow this order hereafter). The two tribes of Prostantheroideae, Chloantheae and Westringieae, were each recovered as monophyletic and sister taxa with strong support (100%, 1.00). The second clade of Lamiaceae consisted of Nepetoideae, Symphorematoideae, Viticoideae, Tectonoideae, Premnoideae, Ajugoideae, Peronematoideae, Scutellarioideae, Cymarioideae, and Lamioideae (Fig. 1; Additional files 4, 5, 6, 7: Figs. S2, S3, S4, S5).

Within Nepetoideae (100%, 1.00), the monophyly of Elsholtzieae, Ocimeae, and Mentheae was robustly supported in all analyses (100%, 1.00). However, relationships among the three tribes varied among different datasets. Most of the datasets (CRM, CR, CR3, dePCS) supported Elsholtzieae as sister to Ocimeae (Fig. 1, 86%, 1.00; Additional files 4, 5: Figs. S2, S3; Additional file 7: Fig. S5), while in the phylogeny based on dataset CR12, Elsholtzieae were weakly supported as sister to Mentheae (Additional file 6: Fig. S4, 45%, 0.66).

In tribe Elsholtzieae, the genus *Elsholtzia* Willd. was recovered as sister to *Collinsonia* L. and *Perilla* L., and the sister relationships received maximal support in all analyses (Fig. 1; Additional files 4, 5, 6, 7: Figs. S2, S3, S4, S5). Representatives of all seven subtribes of Ocimeae formed a well-resolved clade, with subtribe Siphocranioninae (*Siphocranion* spp.) diverging first, followed by subsequent bifurcations for subtribes Lavandulinae (*Lavandula* spp.), Hanceolinae (*Hanceola exserta* Y.Z. Sun ex C.Y. Wu), Isodoninae (*Isodon* spp.), Hyptidinae (*Mesosphaerum suaveolens* (L.) Kuntze), Ociminae (*Ocimum* spp.), and Plectranthinae (*Coleus* spp.). Relationships within tribe Mentheae were also well resolved (100%, 1.00), with subtribe Salviinae recovered as sister to the remaining four subtribes, Prunellinae, Lycopinae, Menthinae, and Nepetinae.

Along the backbone of the tree, subsequent to the branching of the Nepetoideae, Symphorematoideae (100%, 1.00) and Viticoideae (100%, 1.00) formed a clade (i.e., Viticisymphorina sensu Li et al. [19]), which was followed by subsequent bifurcation supporting clades of the Tectonoideae (100%, 1.00), Premnoideae (100%, 1.00), and then Ajugoideae, respectively (Fig. 1, 100%, 1.00). Ajugoideae (100%, 1.00) were divided into four subclades that corresponded with the structure of tribal classification: each tribe was recovered as monophyletic and provided with high branch support (100%, 1.00). Within the Ajugoideae, Rotheceae were recovered as sister to the Teucrieae, Clerodendreae, and Ajugeae.

The sister clade of Ajugoideae was comprised of Peronematoideae, Scutellarioideae, Cymarioideae, and Lamioideae (i.e., the phylogenetically defined Perolamiina in Li et al. [19]). Monophyly of Ajugoideae plus Perolamiina was supported in all analyses with moderate support values (Fig. 1, 71%, 0.98; Additional files 4, 5, 6, 7: Figs. S2, S3, S4, S5), and Peronematoideae were recovered as monophyletic (100%, 1.00) and sister to Scutellarioideae + Cymarioideae + Lamioideae (i.e., Scutelamiina sensu Li et al. [19]). Within Scutellarioideae, four out of five genera were included for analyses and the monotypic genus *Wenchengia* C.Y. Wu & S. Chow (100%, 1.00) is sister to the remaining three genera (100%, 1.00). The sister clade of Scutellarioideae consisted of Cymarioideae and Lamioideae (100%, 1.00). Within Lamioideae, Pogostemoneae were the earliest diverging lineage, followed by the Gomphostemmateae, Colquhounieae, Synandreae, Betoniceae, Galeopseae, Stachydeae, Paraphlomideae, Phlomideae, Leonureae, Marrubieae, Leucadeae, and Lamieae; consistent with previously published studies [52, 53], most tribes received maximal support values, although some tribes were only represented by a limited number of species (e.g., Lamieae, Leucadeae, and Leonureae).

## Discussion

It has been more than 20 years since the first attempt was made to employ molecular data as evidence to infer a phylogenetic tree for Lamiaceae, which made use of the *rbcL* region of the chloroplast genome [15]. Subsequently, various phylogenetic analyses have greatly contributed to our understanding of the circumscription, classification, and phylogeny of this family, progressively improving the resolution of relationships [15, 19, 25, 27–31, 44, 46, 52–54, 69]. This study, based on coding plastome sequences, provides the most comprehensive phylogeny of Lamiaceae at the tribal level to date. With increased taxon sampling and a vastly expanded DNA dataset, the results of our plastid phylogeny significantly clarify the remaining ambiguities for all relationships among subfamilies and provide better support for all nodes in the phylogenetic tree at the subfamilial level.

In our phylogenetic analyses, 12 subfamilies are recovered and well-supported as monophyletic (Fig. 1; Additional files 4, 5, 6, 7: Figs. S2, S3, S4, S5). Our results correspond with the most recent phylogenetic study using five cpDNA regions [19] and have resolved the placement of the Nepetoideae, Premnoideae, and Ajugoideae which were previously unknown. Nepetoideae, the largest subfamily of Lamiaceae, is sister to a grade of lineages comprising the Symphorematoideae, Viticoideae, Tectonoideae, Premnoideae, Ajugoideae, Peronematoideae, Scutellarioideae, Cymarioideae, and Lamioideae (Fig. 1). However, our results differ somewhat from those of the Mint Evolutionary Genomics Consortium [20], which used 520 single-copy nuclear genes from 48 Lamiaceae species representing 11 of 12 subfamilies. Their results of the first-diverging lineages were consistent with ours and only differ within the clade of Premnoideae, Ajugoideae, Peronematoideae, Scutellarioideae, Cymarioideae, and Lamioideae, where most of the relationships in their tree were weakly supported. Furthermore, taxon sampling was sparse in their study, and it is possible that additional taxon sampling could alter the subfamilial relationships that their analyses recovered.

Relationships within Lamioideae are also relatively similar with previous broad-scale studies [52, 53], but internal support values from our study are generally higher. Within Lamioideae, five genera (*Betonica*, *Colquhounia*, *Galeopsis*, *Metastachydium*, and *Roylea*) have not previously been assigned tribal status [2, 52, 53]. In addition, the phylogenetic position of *Paralamium* remains unclear [2, 53], since the genus has not been included in any published molecular phylogenetic study. We included three of these genera (*Betonica*, *Colquhounia*, and *Galeopsis*) in our study.

*Colquhounia* is recovered as sister (Fig. 1, 100%, 1.00) to the clade of Synandreae, Betoniceae, Galeopseae, Stachydeae, Paraphlomideae, Phlomideae, Leonureae, Marrubieae, Leucadeae, and Lamieae. The morphological distinctiveness and well-supported phylogenetic position of *Colquhounia* substantiates tribal recognition within Lamioideae as tribe Colquhounieae (see “Taxonomic treatment”).

Corroborating previous phylogenetic studies [52, 53], our chloroplast phylogeny demonstrates that *Galeopsis* and *Betonica* form a clade (Fig. 1, 64%, 0.98) that is sister to the Stachydeae (100%, 1.00). This clade in turn is recovered as sister to a clade of Paraphlomideae, Phlomideae, Leonureae, Marrubieae, Lamieae, and Leucadeae. Using cpDNA markers, Scheen et al. [52] and Bendiksby et al. [53] found this same structure, and our unpublished data based on chloroplast DNA markers (M. Bendiksby and Y. Salmaki, in prep.) also suggests these two genera occupy different positions within Lamioideae. In contrast, analyses using the low-copy nuclear pentatricopeptide repeat (*PPR*) region recovered *Galeopsis* as sister to tribe Synandreae rather than sister to *Betonica*, albeit this was provided with low support [69]. With the available evidence (see “Discussion”), the phylogeny supports that *Betonica* and *Galeopsis* are distinct from other tribes. As suggested by Li and Olmstead [51], “for the benefit of those who need a complete, rank-based classification of Lamiaceae to arrange genera and species in checklists”, a new monotypic tribe (i.e., Betoniceae) is established here and the tribe Galeopseae (also monotypic) is resurrected, to accommodate the systematic positions of these two genera within Lamioideae. The tribal placement of the remaining three genera, *Paralamium*, *Roylea*, and *Metastachydium*, is still uncertain.

Within Ajugoideae, we recover the same relationships as reported by Xiang et al. [54], who sampled 51 taxa representing 22 of the 23 genera of the subfamily and identified four main clades. All clades are recovered as monophyletic and receive better resolution (Fig. 1). Although Xiang et al. [54] improved our understanding of relationships within Ajugoideae, a tribal classification scheme for the subfamily has been needed. Corroborating previous studies [54], we propose a formal tribal classification for subfamily Ajugoideae, including the new tribe Rotheceae (see “Taxonomic treatment”).

The advances in our knowledge reported in the results above cement a foundation in our understanding of relationships within Lamiaceae. In order to provide a clearer picture in light of these results and to consolidate the numerous advances made in the systematics of Lamiaceae since Harley et al. [1], the following sections provide a detailed discussion and commentary for each subfamily and tribe.

### Subfamily Prostantheroideae Luerss.

Prostantheroideae consist of approximately 315 species allocated to two tribes: Chloantheae and Westringieae. They are distinguished from all other subfamilies by having a prominent albuminous seed [4, 8]. While multiple cell layers can be found in the endosperm in other subfamilies [70] (therefore technically albuminous), the endosperm never develops to a size that can be easily seen [1].

Although confined to Australia, Prostantheroideae are widely distributed throughout most of the continent, in both temperate and tropical climates. Within this expanse, the habitats they occupy range from riparian zones of cool temperate rainforest to crests of shifting sand dunes in the central arid region.

Prostantheroideae are sister to Callicarpoideae (i.e., Calliprostantherina sensu Li et al. [19]). This relationship was first discovered by Olmstead et al. [71], then consistently supported by subsequent molecular phylogenetic studies [18–20, 31, 72] as well as our own (Fig. 1; Additional files 4, 5, 6, 7: Figs. S2, S3, S4, S5). Together, both Prostantheroideae and Callicarpoideae form a sister clade to the remaining Lamiaceae (Fig. 1) [18–20, 31, 72]. In addition to having albuminous seeds, Prostantheroideae are distinguished from Callicarpoideae by their dry fruits (vs. fleshy fruits).

#### Tribe Chloantheae Benth. & Hook. f

Chloantheae consist of 13 genera and ca. 100 species of shrubs (or subshrubs) distributed across mainland Australia [73]. This distribution includes a large number of species adapted to extreme arid habitats, with genera such as *Newcastelia* F. Muell. and *Dicrastylis* Drumm. ex Harv. occupying sandy deserts of the central inland [74].

A remarkable diversity in floral morphology is displayed across Chloantheae, with corollas ranging from 5-merous and zygomorphic (e.g., *Chloanthes* R. Br. and *Dasymalla* Endl.) to 5–8 (–10)-merous and actinomorphic (e.g., *Dicrastylis*). All species are distinguished (particularly from the sister tribe Westringieae) by an unlobed ovary, which develops into a 1 (–2) seeded dry indehiscent fruit [1], and a distinctive indumentum of complex dendritic trichomes (typically tomentose) covering branches, leaves, and flowers (except four species in the Westringieae).

Many taxonomic changes have been made for Chloantheae and its constituents. Since the description of *Chloanthes* and *Pityrodia* R. Br. [75], most genera were shuffled between different tribes of Verbenaceae [76, 77]. Most were allocated within the tribe Chloantheae (Verbenaceae) by Bentham [4]. This treatment was followed later by Hutchinson’s recognition as family Chloanthaceae [78], which was accepted by some authors [74, 79–83], but not all [84, 85].

Phylogenetic analysis of morphological [9] and molecular data [71] indicated that Chloantheae is sister to Westringieae within Lamiaceae, which is supported here (Fig. 1). The contemporary understanding of generic relationships within the tribe was informed by the comprehensively sampled molecular phylogeny of Conn et al. [24], which found that *Pityrodia* was not monophyletic, precipitating the description of *Muniria* N. Streiber & B.J. Conn and restoration of *Dasymalla* and *Quoya* Gaudich. [73]. Another new genus, *Apatelantha*, was recently described to accommodate a clade identified by Conn et al. [24] composed of individuals formerly assigned to *Lachnostachys* Hook., *Newcastelia*, and *Physopsis* Turcz. [86]. Although our study only samples three taxa in Chloantheae, as in previous studies [73], it supports the close relationship between *Dasymalla* and *Chloanthes* relative to *Dicrastylis* (Fig. 1; Additional files 4, 5, 6, 7: Figs. S2, S3, S4, S5).

#### Tribe Westringieae Bartl.

Westringieae consist of five genera and over ca. 210 species of subshrubs, shrubs, and small trees distributed across Australia [1]. Frequently found restricted to exposed and rocky or well-drained places, members of the tribe are distributed throughout habitats within which these places occur, from rainforests to ranges of the Australian arid inland.

Flowers are 5-merous and weakly to strongly zygomorphic, similar to bird or insect pollination syndromes typically found in other Lamiaceae [87–89]. The tribe can be distinguished from Chloantheae by a four-lobed ovary, which develops into four nutlets [1]. The variation in anther morphology (e.g., outgrowth of the antheridial connective of *Prostanthera* Labill.) combined with reductions in fertility (reduction of abaxial stamens to staminodes in *Westringia* Sm.) in this tribe distinguishes it from Chloantheae (which typically has four bithecate anthers) and assists with informing the contemporary generic delimitation in the tribe [1, 90].

Tribal recognition of Westringieae and its generic constituency was first described by Bentham [91]. The monophyly of this tribe, in addition to its sister relationship to Chloantheae, has been substantiated by numerous phylogenetic analyses [9, 19, 71] including our own (Fig. 1). Further investigation into generic relationships has shown that *Hemiandra* R. Br., *Hemigenia* R. Br., *Microcorys* R. Br., and *Westringia* are closely related to each other with respect to *Prostanthera* [87, 90, 92], although the relationship between them still needs to be resolved by more comprehensively sampled phylogenetic studies.

### Subfamily Callicarpoideae Bo Li & R.G. Olmstead

This recently described subfamily consists only of the genus *Callicarpa* which contains ca. 170 species of small trees or shrubs primarily distributed in tropical to temperate Asia, tropical and subtropical America, Australia, and some Pacific Islands [19, 51]. Callicarpoideae differs from other subfamilies by having a peltate or capitate stigma and a drupaceous fruit with four stony pyrenes [51]. Furthermore, Callicarpoideae possess actinomorphic flowers which are unusual within Lamiaceae (generally zygomorphic). The group is remarkably morphologically homogeneous given its broad geographical distribution, although there is variation in the number of flower parts and stamen structure among different species within Callicarpoideae.

*Callicarpa* was historically placed in Verbenaceae and treated as a member of tribe Callicarpeae in subfamily Viticoideae [5]. It was first transferred to Lamiaceae based on a cladistic analysis of morphological, anatomical, and palynological characters [9, 10] and later confirmed by molecular study [19]. Because only one or few representatives of the genus were included, different phylogenetic analyses resolved *Callicarpa* in different positions within Lamiaceae [19, 31, 52, 53, 71].

The sister relationship between *Callicarpa* and Prostantheroideae was first discovered by Olmstead et al. [71] and confirmed by subsequent studies [18–20, 31, 72]. In our analyses, they form a well-supported clade, which is sister to the remaining Lamiaceae (Fig. 1; Additional files 4, 5, 6, 7: Figs. S2, S3, S4, S5).

### Subfamily Nepetoideae (Dumort.) Luerss.

Nepetoideae are the most species-rich subfamily within Lamiaceae, with about 3400 species divided into three tribes, Elsholtzieae, Mentheae, and Ocimeae [1]. Nepetoideae are native to every continent except Antarctica and are found in each of the seven global regions of high Lamiaceae diversity [1, 93]. Although only clarified when comparative pollen analyses were established [6, 8], Nepetoideae are now considered among the most clearly defined subfamilies of Lamiaceae and have consistently been supported as monophyletic in molecular analyses [15, 19, 31, 44, 94, 95]. Nepetoideae contain nearly all the aromatic species within Lamiaceae and are characterized by hexacolpate, trinucleate pollen [6, 8], an investing embryo [96], and the presence of rosmarinic acid [1]. Additionally, mucilaginous nutlets are only known to occur in the Nepetoideae within Lamiaceae and occur in all three tribes [97]. Thus, mucilaginous nutlets may also represent a synapomorphy within Nepetoideae.

The tribal assignment for groups now in Nepetoideae has been controversial [4, 5, 7] and was summarized by Cantino [10]. Results from morphological and molecular studies [9, 10, 95] led to a fundamentally new tribal classification for Nepetoideae proposed by Cantino et al. [11]. They recognized the four tribes Elsholtzieae, Ocimeae, Lavanduleae, and Mentheae, with the latter containing the largest number of changes in circumscription. Harley et al. [1] basically adopted this treatment of Cantino et al. [11], with the exception of subsuming Lavanduleae within Ocimeae. Although the three tribes of Harley et al. [1] are well-supported in both previous studies [16, 23, 27, 31, 98] and our analyses (Fig. 1; Additional files 4, 5, 6, 7: Figs. S2, S3, S4, S5), relationships among the three tribes remain murky. Previous studies have either found (1) Ocimeae to be sister to the Mentheae-Elsholtzieae clade [95], or (2) Mentheae to be sister to the Ocimeae-Elsholtzieae clade [16, 23, 27, 98], or (3) Elsholtzieae to be sister to the Mentheae-Ocimeae clade [31]. Our results reveal that Elsholtzieae is sister to Ocimeae in most of the analyses (CRM, CR, CR3, dePCS) (Fig. 1; Additional files 4, 5, 7: Figs. S2, S3,  S5), but is weakly supported as sister to Mentheae by the dataset CR12 (Additional file 6: Fig. S4). Since none of the abovementioned relationships are strongly supported, nor a broad sampling within all three tribes are included in these studies, further studies are still needed to resolve the relationships among the three tribes.

#### Tribe Elsholtzieae (Burnett) R.W. Sanders & P.D. Cantino

Elsholtzieae are the smallest tribe of Nepetoideae, comprising eight genera and ca. 70 species mostly distributed across East and Southeast Asia. *Collinsonia*, which is restricted to eastern North America, is the sole New World member of this tribe [1, 98]. Species of Elsholtzieae share divergent stamens, a weakly 2-lipped corolla, and an asymmetric disc with an elongate anterior lobe, but it is unclear whether these features are apomorphic [1, 31].

The tribe was formally validated by Sanders and Cantino [99] and consisted of six genera in the classification of Cantino et al. [11]: *Collinsonia*, *Elsholtzia*, *Keiskea* Miq., *Mosla* (Benth.) Buch.-Ham. ex Maxim., *Perilla*, and *Perillula* Maxim. In the molecular phylogenetic study of Nepetoideae by Wagstaff et al. [95], Elsholtzieae was represented by *Elsholtzia*, *Collinsonia*, and *Perilla* and formed a well-supported clade. Based on a sampling of all genera of Elsholtzieae using two nrDNA and four cpDNA markers, the results by Chen et al. [31] confirmed that the previously *incertae sedis* genus *Ombrocharis* is a member of the tribe and sister to *Perillula*. Contemporaneously, based on results from molecular phylogenetic analyses [31] and karyological studies [100], Mayta-Anco et al. [101] established a new genus, *Vuhuangia* Solomon Raju, Molinari & Mayta, to accommodate *Elsholtzia flava* (Benth.) Benth. and *E. penduliflora* W.W. Sm. However, Li et al. [98], apparently unaware of *Vuhuangia,* demonstrated that *Elsholtzia* was not monophyletic and outlined *E. flava* and *E. penduliflora* should be separated from *Elsholtzia* as a distinct genus.

Biogeographic analysis of an expanded sample of Elsholtzieae showed that the tribe originated in East Asia and then dispersed to Southeast Asia and North America; the uplifts of the Qinghai-Tibetan Plateau and climate changes from Middle Miocene onwards may have promoted the species diversification of Elsholtzieae [98].

#### Tribe Ocimeae Dumort.

Ocimeae are characterized by declinate stamens lying along the anterior lip of the corolla and synthecous anthers [1, 102]. As currently circumscribed, a total of 43 genera and over 1200 species are included in Ocimeae, distributed mainly in the tropics and subtropics [1, 103, 104]. Major centers of diversity include tropical Africa and Madagascar, China and Malaysia, and South America [1, 103].

In early classifications of Lamiaceae [4, 5], Ocimeae were recognized as subfamily Ocimoideae. Based on an expansive morphological cladistic analysis, Cantino [9, 10] reduced Ocimoideae to tribe Ocimeae within subfamily Nepetoideae sensu Cantino et al. [11]. Ocimeae was further divided into three subtribes: Hyptidinae, Plectranthinae, and Ociminae [11]. Because *Isodon* (Schrad. ex Benth.) Spach, *Hanceola* Kudô, and *Siphocranion* Kudô are very different from other Ocimeae in terms of nutlet, inflorescence, and calyx morphology, Paton and Ryding [102] treated the three genera as *incertae sedis* within Ocimeae, while Harley et al. [105] later established subtribe Hanceolinae to accommodate them.

Paton et al. [23] carried out the first molecular phylogenetic analyses of Ocimeae and revealed that the genus *Lavandula* L. was sister to the remaining Ocimeae and thus subtribe Lavandulinae was recognized within Ocimeae [23]. However, the two genera *Hanceola* and *Siphocranion* were not included in their analysis. The phylogenetic relationships within Ocimeae were further elucidated based on more comprehensive sampling by Zhong et al. [106], who demonstrated that *Siphocranion*, *Hanceola*, and *Isodon* each formed a distinct lineage within Ocimeae. The subtribes Siphocranioninae and Isodoninae were thus described to accommodate *Siphocranion* and *Isodon*, respectively, while subtribe Hanceolinae only includes *Hanceola* [106].

Recently, Chen et al. [107] reported a new species of *Siphocranion*, and in their molecular phylogenetic analyses based on six cpDNA markers, Siphocranioninae is shown to be sister to the remaining subtribes, with Lavandulinae further supported as the sister group of the clade including Hanceolinae, the Isodoninae-Hyptidinae clade, and the Plectranthinae-Ociminae clade. Our phylogenomic analyses largely confirm the results of Chen et al. [107], with the exception that Isodoninae is resolved as sister to the Hyptidinae-Ociminae-Plectranthinae clade (Fig. 1; Additional files 4, 5, 6, 7: Figs. S2, S3, S4, S5).

#### Tribe Mentheae Dumort.

Mentheae are characterized by stamens divergent or ascending (not declinate), a distinctly 2-lipped corolla (rarely weakly so), symmetric disc (if asymmetric and anterior lobe elongate, then corolla distinctly 2-lipped), and nutlets with an areolate abscission scar. Some of the most widely known medicinal and culinary plants are found within this group: mint, oregano, sage, savory, and thyme. Mentheae comprise both the largest number of genera and species of any tribe within Nepetoideae and Lamiaceae. Many of the plants in this group are of economic and ecological importance and thus have commonly attracted the attention of scientists. This has resulted in fundamentally differing taxonomic approaches at all taxonomic ranks, making it difficult to provide accurate numbers for genera (about 60) or species (at least 2000).

Due to the abovementioned fluidity regarding circumscription within Mentheae, the classification of Harley et al. [1] is regarded as the starting point for a modern subtribal classification. There, three subtribes were recognized, Menthinae, Nepetinae, and Salviinae, along with two genera of uncertain placement (*Heterolamium* C.Y. Wu and *Melissa* L.). Since the treatment of Harley et al. [1], relationships within Menthinae have been greatly clarified based on molecular phylogenetic studies [25, 27, 108–110]. Drew and Sytsma [27] accommodated *Cleonia* L., *Horminum* L., and *Prunella* L. in Prunellinae and erected a new subtribe, Lycopinae, for the enigmatic genus *Lycopus* L. (a tribe Lycopeae was previously proposed [111]). *Neoeplingia* Ramamoorthy, Hiriart & Medrano along with *Melissa* were transferred to Salviinae [27] while *Hyssopus* L. and the previously unplaced *Heterolamium* were included in Nepetinae based on morphological [112] and molecular results [27, 113]. The currently accepted number of subtribes is thus five. This is also well-supported by our analyses, where Salviinae is sister to the other four subtribes; among the remaining subtribes, Nepetinae and Menthinae are sister groups, with Prunellinae and Lycopinae as successive sister groups to Nepetinae and Menthinae (Fig. 1; Additional files 4, 5, 6, 7: Figs. S2, S3, S4, S5).

### Subfamily Symphorematoideae Briq.

Symphorematoideae contain about 21 species in three genera of woody climbers, *Congea* Roxb., *Sphenodesme* Jack, and *Symphorema* Roxb., and occur mainly in tropical regions of Asia. Symphorematoideae are characterized by having capitate cymes surrounded by bracteoles which are often conspicuous, colorful, and accrescent, and incompletely 2-locular ovaries [19].

Historically, Symphorematoideae has been treated as a separate family with the same circumscription [114, 115] or (more commonly) as part of Verbenaceae [5, 116]. It was first found to be related to Lamiaceae in the molecular era [15, 16], and then transferred to Lamiaceae and treated as a subfamily [1, 117]. Li et al. [19] were the first to include all three genera of Symphorematoideae in a comprehensive phylogenetic analysis of Lamiaceae based on chloroplast sequences, and Symphorematoideae was found to be monophyletic and sister to Viticoideae. Such a sister relationship was further recovered in phylogenetic analyses based on nuclear genes [20] and confirmed in our phylogenomic analyses using plastome sequences (Fig. 1; Additional files 4, 5, 6, 7: Figs. S2, S3, S4, S5).

### Subfamily Viticoideae Briq.

Viticoideae currently include ca. 280 species in three genera: *Vitex* (250 spp.), *Teijsmanniodendron* Koord. (23 spp.), and *Pseudocarpidium* Millsp. (9 spp.). These genera are distributed predominantly in the Tropics with a few species of *Vitex* occurring in temperate regions of the Northern Hemisphere [19].

Viticoideae as defined by Briquet [5] were a heterogeneous group whose circumscription has shrunk dramatically. Segregated from traditional Viticoideae are three subfamilies, Callicarpoideae, Premnoideae, and Tectonoideae in the present classification, and part of Ajugoideae and Scutellarioideae. Furthermore, the type genus of Viticoideae, *Vitex*, has expanded to include *Paravitex* H.R. Fletcher, *Petitia* Jacq., *Tsoongia* Merr., and *Viticipremna* H.J. Lam based on molecular studies [19, 42]. Even though only three genera remain in Viticoideae as currently circumscribed, the  intergeneric relationships are still questionable, with the positions of *Teijsmanniodendron* and *Pseudocarpidium* poorly resolved [19]. As mentioned above, the sister relationship between Viticoideae and Symphorematoideae is firmly supported, and the two subfamilies share several anatomical traits [19]. Morphologically, species of Viticoideae can be easily recognized by the palmately compound leaves and dry or fleshy drupes or schizocarps.

### Subfamily Tectonoideae Bo Li & R.G. Olmstead

Tectonoideae comprise only the three species of *Tectona.* They are large trees native to tropical Asia from India to Southeast Asia, but are widely cultivated and naturalized in Africa, Central and South America, and the Caribbean [51].

*Tectona* was originally placed in tribe Tectoneae of Viticoideae [5], but was revealed to be sister to a large clade comprising Lamioideae, Cymarioideae, Scutellarioideae, Peronematoideae, Ajugoideae, and Premnoideae [19]. The relationship is also confirmed by our analyses (Fig. 1; Additional files 4, 5, 6, 7: Figs. S2, S3, S4, S5). However, *Tectona* was recovered as sister to a larger clade including the aforementioned subfamilies (Cymarioideae not sampled) as well as Symphorematoideae and Viticoideae in an analysis using low-copy nuclear markers [20]. Regardless of phylogenetic position, Tectonoideae represents a genetically isolated clade in Lamiaceae and has a series of distinct morphological traits [19, 51].

### Subfamily Premnoideae Bo Li, R.G. Olmstead & P.D. Cantino

Premnoideae were recently established to include three former viticoid genera (Sensu Harley et al. [1]): *Cornutia*, *Gmelina*, and *Premna* [19], with the total species number estimated at about 150 (B. Li, *pers. comm.*). Nearly all species of this subfamily are woody shrubs, trees, or climbers, occurring mainly in Old World tropical to subtropical regions (*Gmelina* and *Premna*) and the New World Tropics (*Cornutia*) [19].

With the current circumscription, Premnoideae are well-supported in our phylogenomic trees (Fig. 1; Additional files 4, 5, 6, 7: Figs. S2, S3, S4, S5). However, in a phylogeny of Lamiaceae based on nuclear genes, *Cornutia* was not recovered in Premnoideae but was sister to the Lamioideae-Ajugoideae-Peronematoideae-Scutellarioideae clade [20, 72]. In the analyses of Li et al. [19], the relationships among Premnoideae, Ajugoideae, and Lamioideae-Cymarioideae-Scutellarioideae-Peronematoideae were not well resolved, but in our phylogenomic analyses, Premnoideae are strongly supported to be sister to the clade comprising Lamioideae, Cymarioideae, Scutellarioideae, Peronematoideae, and Ajugoideae (Fig. 1; Additional files 4, 5, 6, 7: Figs. S2, S3, S4, S5).

### Subfamily Ajugoideae Kostel.

Ajugoideae are the third-largest subfamily within Lamiaceae and contain about 770 species in 23 genera [19, 48, 54, 118, 119] distributed worldwide but most common in tropical regions [1]. A possible synapomorphy of Ajugoideae may be pollen with branched to granular columellae [9].

Briquet [5] first elevated tribe Ajugeae sensu Bentham [4] to subfamilial rank, which was followed by most subsequent treatments [1, 7, 116, 120]. Circumscription of Ajugoideae, however, has changed considerably. The recognition of some subfamilies (i.e., Teucrioideae and Caryopteridoideae) that include many traditionally verbenaceous genera (e.g., *Caryopteris* Bunge, *Clerodendrum* L., *Schnabelia* Hand.-Mazz., and *Teucrium* L.) was untenable. These genera were later transferred to Ajugoideae based on molecular phylogenetic [15, 16] and morphological evidence [121].

A recent phylogenetic study that sampled 22 out of the 23 genera of Ajugoideae and used four cpDNA markers (*matK*, *rbcL*, *trnL*-*trnF*, and *rps16*) strongly supported the monophyly of Ajugoideae and identified four major clades [54]. Relationships among these clades are consistent with the results in our study.

Currently, no tribal classification has been assigned for Ajugoideae. Although some old tribal names have been proposed [5, 91, 122], the circumscription of Lamiaceae at that time was much narrower compared to our current understanding, and many genera now placed within Ajugoideae (e.g., *Caryopteris*, *Clerodendrum*, *Rotheca*, *Schnabelia*, *Volkameria* L.) were previously treated as members of Verbenaceae. Based on results from both the present and previous studies [19, 54], we suggest that the four clades be recognized as tribes Ajugeae, Clerodendreae, Teucrieae, and Rotheceae, with the last proposed here as a new tribe (see “Taxonomic treatment” below).

#### Tribe Rotheceae

Rotheceae are established as a new tribe (see “Taxonomic treatment” below) comprising four genera: *Rotheca* (60 spp.), *Glossocarya* Wall. ex Griff. (13 spp.), *Discretitheca* P.D. Cantino (1 sp.), and *Karomia* Dop. (9 spp.). The tribe is disjunctly distributed from Australia (Queensland) and tropical southern Asia to southern Africa. No non-molecular synapomorphy has been found for this tribe.

*Rotheca*, the largest genus in this tribe, was resurrected by Steane and Mabberley [123] to maintain the monophyly of the genus *Clerodendrum* [35]. In the present study, we demonstrate *Rotheca* to be sister to all other members of the subfamily, as reported by Yuan et al. [124]. Although only *Rotheca* was sampled here, a close relationship to the other three genera has been demonstrated previously [54]. Steane et al. [36] found *Karomia* to be sister to *Rotheca* based on *ndhF* sequences, and this relationship was corroborated by Li et al. [19] based on five cpDNA markers. Xiang et al. [54] found that *Karomia*, *Discretitheca*, *Glossocarya*, and *Rotheca* formed a clade, but with moderate support. *Discretitheca* and *Glossocarya* were only first included in molecular phylogenetic analyses [54], and detailed morphological studies as well as molecular phylogenetic studies for these two genera are scarce and more studies are needed. As with *Discretitheca* and *Glossocarya*, only one species of *Karomia* (*K. speciosa* (Hutch. & Corbishley) R. Fern*.*) has been included in previous molecular phylogenetic analyses [36, 54], although DNA sequences of two species have been reported (the additional species is *K. tettensis* (Klotzsch) R. Fern. which was used mainly for ecological analyses [125]). Overall, the systematic relationships within this tribe await to be fully clarified.

#### Tribe Teucrieae Dumort.

Teucrieae consist of ca. 260 species in three genera, *Teucrium* (ca. 250 spp.), *Schnabelia* (5 spp.), and *Rubiteucris* Kudô (2 spp.). The latter two genera are endemic to East Asia, while *Teucrium* has a subcosmopolitan distribution. A possible synapomorphy of the tribe is the confluence of anther thecae at anthesis, a feature that also characterizes Ajugeae, where it may have arisen independently.

*Teucrium* is the largest genus in this tribe. A previous phylogenetic study [48] suggested the inclusion of *Oncinocalyx* F. Muell., *Spartothamnella* Briq., and *Teucridium* Hook.f. in *Teucrium*, and this treatment was confirmed by Xiang et al. [54]. Although both *Rubiteucris* and *Schnabelia* are small genera, the taxonomy and systematic relationships of *Rubiteucris* and *Schnabelia* were not sufficiently resolved until recent molecular phylogenetic studies based on a broad sampling [48, 54]. Here, the monophyly of Teucrieae is strongly supported (Fig. 1; Additional files 4, 5, 6, 7: Figs. S2, S3, S4, S5).

#### Tribe Ajugeae Benth.

Ajugeae contain 79 species in six genera: *Ajuga* L. (ca. 50 spp.), *Amethystea* L. (1 sp.), *Caryopteris* (7 spp.), *Pseudocaryopteris* (Briq.) P.D. Cantino (3 spp.), *Trichostema* Gronov. (17 spp.), and *Tripora* P.D. Cantino (1 sp.). *Ajuga* is distributed primarily in Eurasia, *Amethystea* is widespread in temperate Asia [1], *Trichostema* is restricted to North America [126], and the remaining three genera are endemic to East Asia. A possible synapomorphy is the confluence of the anther thecae at anthesis (with a reversal in *Caryopteris*), a feature that also characterizes Teucrieae and may have arisen independently in the two tribes. In most other species of Ajugoideae and in most of the closest outgroups, the thecae remain separate at anthesis. However, it is equally parsimonious to hypothesize that confluent anther thecae are a synapomorphy of the clade comprising Ajugeae, Clerodendreae, and Teucrieae, with a subsequent reversal at the base of Clerodendreae.

The traditionally delimited genus *Caryopteris* [5, 54, 127] is polyphyletic [9, 128] and species previously included in *Caryopteris* have been distributed in six genera: *Caryopteris*, *Discretitheca*, *Pseudocaryopteris*, *Rubiteucris*, *Schnabelia*, and *Tripora*, of which three were placed in tribe Ajugeae, two belong to tribe Teucrieae, and one belongs to tribe Rotheceae. A sister-group relationship between *Tripora* and *Pseudocaryopteris* was inferred in previous studies [54, 129, 130], but support values varied in different studies. The sister relationship between the North American genus *Trichostema* and the East Asian genus *Caryopteris* was also reported in many studies [15, 16, 35, 36, 130, 131]. Although *Ajuga* is the largest genus in this tribe, no phylogenetic study has been carried out for the genus to date, and infrageneric relationships within this genus still need further investigation.

#### Tribe Clerodendreae Briq.

Clerodendreae consist of ca. 350 species in ten genera: *Clerodendrum* (ca. 150 spp.), *Volkameria* (30 spp.), *Kalaharia* Baill. (1 sp.), *Amasonia* L.f. (8 spp.), *Tetraclea* A. Gray (2 spp.), *Aegiphila* Jacq. (120 spp.), *Ovieda* L. (21 spp.), *Oxera* Labill. (21 spp.), *Hosea* Ridl. (1 sp.), and probably *Monochilus* Fisch. & C.A. Mey. (2 spp.). *Monochilus* has not been included in any published molecular analysis, but based on a cladistic analysis of morphological data, Cantino [9] suggested a close relationship between *Monochilus* and *Amasonia*. Both genera usually have alternate to subopposite leaves, a rare feature in Lamiaceae. *Monochilus* was not included in the molecular results presented here but the presence of alternate to subopposite leaves suggests that *Monochilus* should be treated within tribe Clerodendreae. However, this relationship needs to be tested using molecular evidence.

Clerodendreae are pan-tropical/subtropical in distribution, predominantly distributed in the Americas, Africa, Asia, and Pacific Oceania. A probable synapomorphy for the tribe is a drupaceous fruit with four one-seeded pyrenes. In some species, the fruits split into four fleshy schizocarps. A similar fruit type is found in *Rotheca* (Tribe Rotheceae), where it apparently evolved independently. The character polarity is not entirely clear because Premnoideae also have drupaceous fruits. However, the fruits of Premnoideae contain a single four-seeded pyrene instead of four one-seeded ones. The other closely related groups (subfamilies Peronematoideae, Scutellarioideae, Cymarioideae, and Lamioideae) have dry fruits [19].

In terms of the number of genera, this is the largest tribe within subfamily Ajugoideae. Previous molecular phylogenetic studies concentrated mainly on two genera, *Clerodendrum* [34–36, 124] and *Oxera* [118, 119]. As a result of the disintegration of the traditionally defined *Clerodendrum*, some genera (i.e., *Volkameria*, *Ovieda*, *Rotheca*) were resurrected [34–36, 123, 124]. Species relationships within those genera, however, remain uncertain. In addition, relationships within the clade including *Ovieda*, *Aegiphila*, *Clerodendrum*, *Tetraclea*, *Amasonia*, *Kalaharia*, and *Volkameria*, require further study.

### Subfamily Peronematoideae Bo Li, R.G. Olmstead & P.D. Cantino

Peronematoideae were recently established to accommodate a well-supported clade comprising four small, mostly tropical Asian genera, *Garrettia* (1 sp.), *Hymenopyramis* (7 spp.), *Peronema* (1 sp.), and *Petraeovitex* (8 spp.), which are sister to a larger clade formed by subfamilies Scutellarioideae, Cymarioideae, and Lamioideae [19]. These four genera were previously placed in the subfamily Caryopteridoideae of Verbenaceae [5, 132, 133] and were all transferred to Lamiaceae by Cantino et al. [11], with *Hymenopyramis* placed in Viticoideae, *Peronema* and *Petraeovitex* in Teucrioideae, and *Garrettia* in Ajugoideae. However, all the four genera were treated as *incertae sedis* in Harley et al.’s classification of Lamiaceae [1].

In recent molecular phylogenetic studies, *Garrettia* was first inferred to be sister to a clade comprising Scutellarioideae, *Acrymia*, *Cymaria*, and Lamioideae [53], while the same sister relationship to an equivalent clade of the Scutellarioideae-*Cymaria*-Lamioideae clade (*Acrymia* was not sampled) was later found for a small well-supported clade comprised of *Hymenopyramis*, *Petraeovitex*, and *Peronema* [31, 44], as confirmed in our phylogenomic trees (Fig. 1; Additional files 4, 5, 6, 7: Figs. S2, S3, S4, S5). When *Garrettia*, *Hymenopyramis*, *Petraeovitex*, and *Peronema* were included in the same analysis, they grouped together in a highly supported clade that is sister to the Scutellarioideae-Cymarioideae-Lamioideae clade [19]. Morphologically, the four genera are very heterogeneous but do share some common traits as noted by Chen et al. [44] and Li et al. [19].

### Subfamily Scutellarioideae (Dumort.) Caruel

Scutellarioideae consist of ca. 390 species in five genera: *Holmskioldia* Retz. (1 sp.), *Wenchengia* (1 sp.), *Renschia* Vatke (1 sp.), *Tinnea* Kotschy ex Hook. f. (19 sp.), and *Scutellaria* L. (ca. 360 spp.) [1, 9, 121]. Species numbers and distribution of these genera are extremely uneven. *Scutellaria* is the largest and most widely distributed genus, having a cosmopolitan distribution [1, 134, 135]. *Tinnea* is much smaller and is distributed in tropical and southern Africa. The monotypic genera *Renschia*, *Wenchengia*, and *Holmskioldia* are endemic to Somalia, Southeast Asia (Hainan Island of China, Vietnam), and subtropical Himalayan regions, respectively. Scutellarioideae is diagnosed by the following synapomorphic characters: pericarps with tuberculate or elongate processes [136], high densities of xylem fibers in the calyces [137], and thyrses with single-flowered cymes that form raceme-like inflorescences (but most species of *Tinnea* and *Holmskioldia* have cymose inflorescences).

Scutellarioideae had been thought to be sister to Lamioideae [31, 44], but with the separation of Cymarioideae from the Lamioideae [19], Scutellarioideae is sister to the Cymarioideae-Lamioideae clade. Based on previous studies and our phylogenomic results, *Tinnea* and *Holmskioldia* are successive sister groups to *Scutellaria*, with *Wenchengia* sister to the rest of Scutellarioideae [15, 16, 19, 31, 44, 66, 136]. However, relationships within Scutellarioideae remain unresolved because *Renschia* has never been included in a molecular phylogenetic study. To date, four phylogenetic studies have focused on *Scutellaria* [66, 138–140], but none included a comprehensive taxon sampling of the genus or of Scutellarioideae as a whole. Thus, relationships within *Scutellaria* still need to be addressed in future studies.

### Subfamily Cymarioideae Bo Li, R.G. Olmstead & P.D. Cantino

Cymarioideae were recently established to include two small genera that have previously been considered *incertae sedis* [1], *Acrymia* (1 sp.) and *Cymaria* (2 spp.), which are endemic to Southeast Asia.

Bendiksby et al. [53] found that *Acrymia* and *Cymaria* were the closest relatives of Lamioideae, which was supported by a subsequent study [44] but only with moderate support. Li et al. [19] further confirmed this relationship with high support values and consequently established a new subfamily, Cymarioideae, to accommodate the systematic position of the *Acrymia*-*Cymaria* clade. In the present study, *Cymaria dichotoma* Benth. is sister to Lamioideae in all analyses (Fig. 1; Additional files 4, 5, 6, 7: Figs. S2, S3, S4, S5).

Regarding the systematic placement of the *Acrymia*-*Cymaria* clade, two different treatments are feasible [19]. The *Acrymia*-*Cymaria* clade could be treated as a separate subfamily or as a distinct tribe within Lamioideae; both options are acceptable based on the principle of monophyly. However, as suggested by Bendiksby et al. [53] and Chen et al. [44], the inclusion of *Acrymia*-*Cymaria* within Lamioideae would make the subfamily morphologically heterogeneous and difficult to diagnose. The apomorphy of axial monochasial cymes which defines Cymarioideae is especially distinct and is not found within Lamioideae. Thus, we concur with the approach of Li et al. [19] and recognize Cymarioideae as a subfamily here.

### Subfamily Lamioideae Harley

Lamioideae are the second largest subfamily within Lamiaceae, containing about 1260 species in 62 genera, with a near-cosmopolitan distribution, though concentrated in Eurasia and northern to tropical Africa [52, 53, 69].

Considerable progress has been made in our understanding of subfamily Lamioideae in recent years. Since Harley et al. [1], one genus has been established (*Rydingia* Scheen & V.A. Albert [141]), four genera have been resurrected (*Acanthoprasium* (Benth.) Spenn. [53]; *Betonica* [52]; *Phlomoides* Moench [142]; *Pseudodictamnus* Fabr. [33]), eight genera have been reduced to synonyms (*Alajja* Ikonn. and *Sulaimania* Hedge & Rech. f. [53]; *Lamiophlomis* Kudô, *Notochaete* Benth., and *Pseuderemostachys* Popov [142]; *Eremostachys* Bunge [28]; *Bostrychanthera* Benth. [43]; *Stachyopsis* Popov & Vved. [143]), and *Holocheila*, which was formerly treated as *incertae sedis* [1], has been shown to belong in Lamioideae [44]. Molecular phylogenies have also established that subfamily Cymarioideae is sister to Lamioideae [19].

A tribal classification of Lamioideae was the result of a molecular phylogeny based on cpDNA [52, 53]. The ten tribes have been corroborated as monophyletic groups using nuclear [143] and low-copy nuclear markers [67]. Four genera remained unplaced in the tribal classification because they formed monogeneric clades [53, 67]; however, two new tribes, i.e., Colquhounieae and Betoniceae, are proposed here to accommodate the genera *Colquhounia* and *Betonica*, respectively. The monotypic *Roylea* has still not been attributed to a tribe. *Roylea* groups within tribe Marrubieae in some nuclear-based phylogenies, but not in all and not in phylogenies based on cpDNA data [33, 53, 67, 143]. To date, only two genera, *Metastachydium* and *Paralamium*, have still not been included in molecular phylogenetic studies of Lamioideae, and their relationship with the other genera remains enigmatic.

#### Tribe Pogostemoneae Briq.

Pogostemoneae consist of 11 genera as currently circumscribed [44, 52, 53], including *Achyrospermum* Blume (25 spp.), *Anisomeles* R. Br. (26 spp. [144]), *Craniotome* Rchb. (1 sp.), *Colebrookea* Sm. (1 sp.), *Comanthosphace* S. Moore (4 spp.), *Eurysolen* Prain (1 sp.), *Holocheila* (1 sp.), *Leucosceptrum* Sm. (1 sp.), *Microtoena* Prain (19 spp.) [145], *Pogostemon* Desf. (80 spp.) [146, 147], and *Rostrinucula* Kudô. (2 sp.), and all genera are monophyletic [44, 52, 53, 145, 148]. Most genera of the tribe are distributed in East Asia to Southeast Asia, with three genera having a disjunct distribution between Asia and tropical Africa (*Pogostemon*, *Achyrospermum*, and *Anisomeles*). In addition, the monotypic genus *Paralamium* Dunn. is probably a member of Pogostemoneae based on the presence of small glossy nutlets [53].

Pogostemoneae were established by Briquet [5] and originally included seven genera (*Elsholtzia*, *Comanthosphace*, *Keiskea*, *Pogostemon*, *Dysophylla* Blume, *Tetradenia* Benth., and *Colebrookea*). Later, Kudô [149] and Press [150] circumscribed Pogostemoneae in a broad sense, adding 11 genera to the tribe [11, 52, 150]. A number of taxonomic and molecular phylogenetic studies [11, 19, 52, 53, 148, 150, 151] have indicated that six genera should be excluded from this tribe and that *Dysophylla* should be merged with *Pogostemon*, as suggested by Hasskarl [152] and Press [150], leaving the present total of 11 genera.

Cantino [10] and Cantino et al. [11] proposed a subfamily named Pogostemonoideae to include *Colebrookea*, *Comanthosphace*, *Leucosceptrum*, *Pogostemon*, *Rostrinucula*, *Anisomeles*, and *Eurysolen*, but with hesitation regarding the two latter genera. Recent molecular phylogenetic studies have shown that Pogostemonoideae are sister to Lamioideae and have been included in that subfamily [52, 53]. Our results recover tribe Pogostemoneae as sister to the clade contain all other members of Lamioideae (Fig. 1). Previous studies based on plastid DNA regions [52, 53] identified two well-supported clades within Pogostemoneae. One clade includes *Eurysolen*, *Leucosceptrum*, *Rostrinucula*, *Comanthosphace*, and *Achyrospermum* and is characterized by having dull and glandular nutlets, and the sclerenchyma region in the pericarp obsolete, indistinct, or absent. The second clade is composed of *Colebrookea*, *Craniotome*, *Microtoena*, *Anisomeles*, and *Pogostemon*. Within this clade, two subclades were recognized [53]. *Colebrookea* is the only genus within the first subclade. This subclade is distinctive by possessing nutlets that are hairy and with eglandular hairs at the apex, while the remaining genera formed a second subclade united by having glossy and glabrous nutlets. Morphological studies focusing on traditionally defined Pogostemoneae (i.e., Pogostemonoideae; [153, 154]) identified some useful taxonomic characters. Subsequently, Scheen et al. [52], while not identifying any morphological synapomorphies, suggested that small and relatively glossy nutlets, pericarps (typically) lacking a sclerenchyma region [153, 154], generally long-exserted stamens with (usually) bearded filaments, a (generally) weakly 2-lipped corolla, and (generally) broad bracts are potentially useful morphological characters in defining the tribe. Further comparative morphological studies combined with well-supported phylogenetic trees based on extensive sampling and additional nuclear loci will be necessary to determine synapomorphies for this tribe.

#### Tribe Gomphostemmateae Scheen & Lindqvist

Gomphostemmateae were established by Scheen et al. [52] to include three genera, *Gomphostemma* Wall. ex Benth. (ca. 36 spp.), *Chelonopsis* Miq. (ca. 16 spp.), and *Bostrychanthera* (2 spp.), that are distributed in temperate to tropical East Asia [1, 155]. Since then, the genus *Bostrychanthera* was subsumed within *Chelonopsis* by Xiang et al. [43] based on morphological [156, 157] and molecular data [43] (see also Bongcheewin et al. [158]), thus leading to only two genera (*Gomphostemma* and *Chelonopsis*) currently retained in this tribe.

Gomphostemmateae were shown to be sister to a large group of Lamioideae in previous studies [52, 53], but these results were equivocal due to suboptimal support values. Here, we find the same relationship but with higher support values (Fig. 1). Possible synapomorphies for the tribe include pollen with branched columellae [159] and fibers in the mesocarp [160, 161]. However, pericarp structure has only been reported in a few species, and it is unclear whether unexamined species share these characters. Thus, future detailed morphological studies are needed.

#### Tribe Colquhounieae

Colquhounieae are newly established here to accommodate the enigmatic *Colquhounia*. The genus comprises approximately five species endemic to the Himalayan massif from Nepal and north India to southwest China and Vietnam. Morphologically, the genus is characterized by having nutlets winged at the apex, which is rare within subfamily Lamioideae [52]; besides *Colquhounia*, only some species of *Chelonopsis* have this character.

Based on trichome morphology, Hu et al. [162] classified the genus into two sections, *Colquhounia* sect. *Simplicipili* C.Y. Wu & H.W. Li (including *C. seguinii* Vaniot) and *C.* sect. *Colquhounia* (all remaining species), but this classification was not supported by molecular phylogenetic results [43]. Although Scheen et al. [52] and Bendiksby et al. [53] found that *Colquhounia* occupied a phylogenetically distinct position within Lamioideae, they kept the genus unclassified at the tribal level, in part because only two species (*C. coccinea* Wall. and *C. elegans* Wall. ex Benth.) and only three markers (*trnL*-*trnF*, *rps16*, and *matK*) were used for phylogenetic reconstruction. In this study, five taxa were included for analyses and they form a distinct clade within Lamioideae. Thus, we describe this clade as a new tribe (see “Taxonomic treatment” below).

#### Tribe Synandreae Raf.

Synandreae were recircumscribed by Scheen et al. [163] to include the following five genera: *Brazoria* Englm. & A. Gray (3 spp.), *Macbridea* Elliott ex Nutt. (2 spp.), *Physostegia* Benth. (12 spp.), *Synandra* Nutt. (1 sp.), and *Warnockia* M.W. Turner (1 sp.). The only morphological synapomorphy for the tribe is a raceme-like inflorescence with sessile or very shortly pedicellate flowers [52, 163]. All five genera are characterized by having villous stamen filaments, but this is also found in some members of tribe Pogostemoneae (e.g., *Pogostemon*, *Anisomeles*) and tribe Stachydeae [1] (*Chamaesphacos* Schrenk ex Fisch. & C.A. Mey).

Previous studies involving cpDNA, nrDNA, and low-copy nuclear markers failed to adequately discern the position of Synandreae within Lamioideae [52, 53, 69, 163, 164]. Our results provide strong support for the placement of Synandreae, with the caveat that only one representative was included (Fig. 1). Only two lamioid tribes include species with a North American distribution, Synandreae and Stachydeae. The two tribes are not closely related [52, 53, 69] and therefore represent separate dispersals into North America [163].

#### Tribe Betoniceae

Betoniceae are newly established here to accommodate the phenetically and genetically isolated genus *Betonica* in Lamioideae. There are nine currently accepted *Betonica* species, three of which include 2–6 subspecific taxa distributed throughout Europe reaching Central Asia and Northwest Africa [165]. *Betonica* has repeatedly been included in, and excluded from, the genus *Stachys* L. Some authors have treated *Betonica* as a distinct genus [166–171], while Bentham [90] and Briquet [5] placed *Betonica* in its own section within *Stachys*. In the most recent morphological classification of *Stachys*, Bhattacharjee [172] recognized *Betonica* as a subgenus (*S*. subg. *Betonica* (L.) Bhattacharjee) within *Stachys*, defined by prominent sterile rosettes, usually unbranched flowering shoots arising from an axillary bud of the rootstock, and deeply crenate to serrate leaf margins; features that *Betonica* shares with the *Stachys* sections *Eriostomum* (Hoffmanns. & Link) Dumort. and *Ambleia* Benth. Yet, Bhattacharjee [172] mentions that *S*. subg. *Betonica* is divergent in the nature of the calyx (sessile calyx) and bracteoles (with a broad hardened base). Tomas-Bárberán et al. [173] points to differences in phytochemistry between *Betonica* and *Stachys* species, as currently circumscribed. Recently, Giuliani and Bini [174] found that *Betonica* possesses only peltate trichomes, while *Stachys* has different types of large capitate hairs that are lacking in *Betonica*. In addition, Giuliani and Bini [174] also found that peltate trichomes of *Betonica* species have unusual secretions composed of flavonoids and essential oils and suggested that *Betonica* should be considered a genus of its own.

An early molecular phylogenetic analysis of *Stachys* s.l. [39] based on both plastid and nuclear DNA sequence data demonstrated that the type species of *Betonica*, *B. officinalis* L. (as *Stachys officinalis* (L.) Trevis.), fell outside of the clade that contained the remainder of *Stachys* including the type species, *S. sylvatica* L. Perhaps not being aware of this, Harley et al. [1] retained *Betonica* in synonymy under *Stachys*. Since then, further molecular phylogenetic evidence has corroborated the distinctness of *Betonica* [52, 53, 69]. Based on results from a comprehensive plastid phylogeny of Lamioideae that included five species of *Betonica*, Scheen et al. [52] suggested *Betonica* should be resurrected from synonymy under *Stachys*. The five species of *Betonica* formed a strongly supported clade sister to *Galeopsis*, the sister relationship, however, receiving low statistical support. This phylogenetic result was corroborated by a follow-up study with more taxa and additional genetic markers [53]. The monophyly and distinctness of *Betonica* has received support also from nuclear data [69], with a weakly supported sister relationship to tribe Synandreae. Since *Betonica* so far has remained unclassified at the tribal level, and the genus seems to lack a clear affinity to any other lamioid taxon, we propose herein that the *Betonica* clade be recognized at the tribal level (see “Taxonomic treatment” below).

The intrageneric classification of *Betonica* into three sections [175, 176] (i.e., *B*. sect. *Betonica*, *B*. sect. *Foliosae* (Krestovsk. & Lazkov) Lazkov, and *B*. sect. *Macrostachya* (R. Bhattacharjee) Krestovsk.), has not received statistical support by any so far published molecular phylogenies [e.g., 52, 53]. *Betonica alopecuros* L., however, receives support as sister to the remaining species in these studies. The distinctness of *B. alopecuros* is also supported by morphology: yellow corollas with bifid upper lip and annulate corolla tubes [172, 177]. A more comprehensive study of this genus is needed.

#### Tribe Galeopseae (Dumort.) Vis.

In the present study, we propose resurrection of the tribe Galeopseae to accommodate the phenetically and genetically isolated genus *Galeopsis* in Lamioideae. Dumortier [167] established subtribe Galeopsinae (as “Galeopsideae”) within the Stachydeae and included the two genera, *Galeopsis* and *Lamium* L. Later, Visiani [178] elevated subtribe Galeopsinae to the rank of tribe (as “Galeopsideae” [98]) but included only *Galeopsis*. *Galeopsis* represents a morphologically highly distinct genus within subfamily Lamioideae, characterized by erect annual herbs with two conical protuberances near the base of the anterior lip of the corolla and anthers dehiscing by two valves, of which the upper is fimbriate [1, 179].

*Galeopsis* comprises 10 currently accepted species, two subspecies, and six documented hybrids [165]. The genus is distributed in temperate Eurasia with a center of species richness in Europe [180]. Strong support for the monophyly of *Galeopsis* was obtained in two recent molecular phylogenetic studies of Lamioideae that included three [52] and eight [53] species of *Galeopsis*, respectively.

Phylogenetically, *Galeopsis* holds a rather isolated, yet uncertain, position and has remained unclassified at the tribal level [52, 53, 69]. In both Scheen et al. [52] and Bendiksby et al. [53], *Galeopsis* was weakly supported as sister to *Betonica*, which in turn was sister to tribe Stachydeae, with even weaker support. Hence, a close relationship to *Lamium* and *Lamiastrum* Heist. ex Fabr., with which *Galeopsis* had been classified in most traditional classifications (e.g., subtribe Galeopsidinae Dumort.) based on the shared feature of a swollen corolla tube, was discarded. The sister relationship between *Betonica* and *Galeopsis* received some support from other sources of data. The two genera share the same base chromosome number [181] (*x* = 8), and flavonoid p-coumaroyl glucosides are present in both *Betonica* and *G.* subg. *Galeopsis* [182]. The placement of *Galeopsis* in the nuclear *PPR* phylogeny by Roy and Lindqvist [69], however, does not support a sister relationship to *Betonica*, and *Galeopsis* falls out largely unresolved in their Lamioideae phylogeny. Although our current plastome-based phylogeny corroborates a sister relationship between *Galeopsis* and *Betonica*, support values remain low (Fig. 1). Based upon this phylogenetic uncertainty, the lack of support from nuclear data, and a goal of achieving taxonomic stability, we resurrect the tribe Galeopseae to encompass the single genus *Galeopsis*.

Reichenbach [169] divided *Galeopsis* into two subgenera, *G.* subg. *Galeopsis* and *G.* subg. *Ladanum* Rchb. Subgenus *Galeopsis*, is readily distinguished from *G.* subg. *Ladanum* by the presence of rigid hairs and swollen stem nodes in the former. The division of the genera into two equally sized subgenera is supported by phytochemistry [182], crossing experiments [183], and molecular phylogenetics [53, 184]. *Galeopsis* subg. *Galeopsis* comprises the following five species: *G. bifida* Boenn., *G. pubescens* Besser., *G. speciosa* Mill., *G. tetrahit* L., and *G*. *sulphurea* Jord. According to molecular analyses by Bendiksby et al. [184], the latter appears to represent a valid species, distinct from *G. speciosa*, and represents the most likely maternal parent to *G. tetrahit* (*G. pubescens* being the paternal parent). *Galeopsis* subg. *Ladanum* comprises the following five species: *G. ladanum* L., *G. nana* Otsch., *G. pyrenaica* Bartl., *G. reuteri* Rchb. f., *G. segetum* Neck. Species within *G.* subg. *Ladanum* have proven indistinguishable in DNA phylogenetic analyses involving nuclear (NRPA2, 5S-NTS) and chloroplast (*matK*, *psbA*-*trnH*, *rps16*, *trnL*-*trnF*, and *trnS*-*trnG*) DNA regions (M. Bendiksby, unpubl.). Morphologically, however, they appear highly distinct, and AFLP data (genomic fingerprint) group accessions according to species [M. Bendiksby, unpubl.]. Hence, the species of *G*. subg. *Ladanum* have probably diverged recently and the multilocus data suffers from incomplete lineage sorting.

#### Tribe Stachydeae Dumort.

Cosmopolitan Stachydeae are the largest and taxonomically most challenging alliance of all recognized tribes in subfamily Lamioideae [29, 30, 52, 53, 69]. Stachydeae have previously been the subject of several molecular phylogenetic investigations [30, 37–39, 52, 53, 185]. Lindqvist and Albert [39] revealed that three genera endemic to Hawaiian (dry fruited *Haplostachys* (A. Gray) W.F. Hillebr., fleshy fruited *Phyllostegia* Benth., and *Stenogyne* Benth.) as well as the genera *Prasium* L., *Phlomidoschema* (Benth.) Vved., and *Sideritis* L. are nested within the large genus *Stachys*. Both *Prasium* with fleshy schizocarp and *Phlomidoschema*, which is characterized by a small corolla and branched hairs, are monotypic [1]. In contrast, *Stachys* comprises about 275 species and *Sideritis* comprises about 125 species [1]. This paraphyly of *Stachys* was corroborated by Scheen et al. [52] who showed that the Asian genera *Chamaesphacos*, *Suzukia* Kudô, and *Thuspeinanta* T. Durand also are embedded within *Stachys* and that the monotypic genus *Melittis* L. represents the sister to all other Stachydeae. In a later work, Bendiksby et al. [53] added *Hypogomphia* Bunge to the list of taxa nested within *Stachys*. Morphologically, the annuals *Chamaesphacos*, *Hypogomphia*, and *Thuspeinanta* are characterized by 1–3-flowered cymes and narrow nutlets, while *Suzukia* is recognized by a creeping habit and racemose inflorescences [1]. Thus, 12 genera and ca. 470 species are currently recognized in Stachydeae, but generic realignments are needed to reflect phylogenetic relationships. Scheen et al. [52] found no non-molecular synapomorphies for this diverse tribe, but listed the following characteristics as common among its members: calyx campanulate or weakly 2-lipped, calyx lobes often spiny, calyx throat often hairy, corolla strongly 2-lipped, anterior pair of stamens bending outwards after pollination, and nutlets usually apically rounded.

Besides some studies focusing on certain groups, such as Hawaiian [39, 185] and New World *Stachys* [30, 69], a comprehensive phylogenetic study of Stachydeae based on multiple loci analyses was performed by Salmaki et al. [29]. Analyses of nuclear ribosomal (nrITS) and plastid DNA data corroborated the monophyly of the tribe, with *Melittis* as sister to all remaining Stachydeae. Salmaki et al. [29] suggested the phylogenetic name “*Eurystachys* Y. Salmaki & M. Bendiksby” for the clade including all genera attributed to Stachydeae except *Melittis*. Although the plastid DNA markers provided well-supported backbone resolution in the *Eurystachys* clade, the nrITS phylogenetic tree recovered several groups with relatively poorly supported and short branches [29]. Therefore, detailed conclusions on the phylogenetic relationships in the *Eurystachys* clade needed using additional nuclear markers.

Recently, phylogenetic relationships in the *Eurystachys* clade utilizing two additional nuclear ribosomal DNA sequences (nrETS and 5S-NTS) provided high resolution allowing recognition of 12 well-supported clades within the *Eurystachys* clade, which also were recovered in the previous phylogenetic analyses using plastid DNA sequences [186]. The 12 clades were formally named in the *Eurystachys* clade following a PhyloCode nomenclature [187] and provided the basis for a future rank-based classification of Stachydeae with two options: (1) splitting the *Eurystachys* clade into 12 individual genera, each based on a pre-existing genus name and redefined to encompass additional taxa, but without clear morphological apomorphies; or (2) lumping of all these formal clades into a broadly defined *Stachys*, including widely recognized and morphologically well-defined segregates such as *Prasium* and *Sideritis* [186]. Clearly, more studies using various sources of evidence are needed to clarify the taxonomic borders in this tribe. A micro-morphological approach [153, 159–161, 188–192] at a global scale may provide a promising supplement to the more traditionally applied macro-morphological approaches.

#### Tribe Paraphlomideae Bendiksby

Paraphlomideae were established by Bendiksby et al. [53] to accommodate *Matsumurella* Makino (5 spp.), *Ajugoides* Makino (1 sp.), and *Paraphlomis* (Prain) Prain (ca. 25 spp.), together which have been found to form a distinct lineage within Lamioideae. Though the tribe has no clear synapomorphy, it can be distinguished from other tribes of Lamioideae by the following set of characters: herbs or subshrubs, indumentum of simple hairs, actinomorphic calyx, corolla (1/3) with hairy upper lip but scarcely bearded along the margin, included stamens, and an apically truncate ovary [1, 53, 193]. Most species of the tribe are restricted to East Asia (south China and Japan), with some species of *Paraphlomis* extending to Southeast Asia [1, 193].

#### Tribe Phlomideae Mathiesen

Based on the most recent molecular phylogenetic study of Phlomideae [28], the tribe now consist of only two genera: *Phlomis* L. (ca. 50–90 spp.) and *Phlomoides* (ca. 150–170 spp.).

Phlomideae were established by Mathiesen in Scheen et al. [52], in which six genera were recognized in the tribe: *Eremostachys*, *Lamiophlomis*, *Notochaete*, *Phlomis*, *Phlomoides*, and *Pseuderemostachys*. Phlomideae are usually characterized by having calyx lobes abruptly narrowed to a narrow apex and expanded at the corolla margins that are bearded and densely pubescent outside and have branched hairs [52]. Mathiesen et al. [142] later reduced *Pseuderemostachys*, *Lamiophlomis*, and one species of *Notochaete* (*N. hamosa* Benth.) to synonyms of *Phlomoides*. Combining multilocus molecular phylogenetic analyses and morphological evidence, Salmaki et al. [28] continued to show that *Eremostachys*, *Notochaete*, and *Paraeremostachys* Adylov, Kamelin & Makhm should all be transferred to *Phlomoides*. Thus, the number of recognized genera in Phlomideae was reduced to two, i.e., *Phlomis* and *Phlomoides*. Species of *Phlomis* are shrubs or subshrubs with simple leaves, laterally compressed, flattened, sickle-shaped, but not fringed or incised upper corolla lips, and with nutlet pericarps possessing a sclerenchyma region (indistinct in a few species). In contrast, *Phlomoides* are herbaceous with simple or laciniate to pinnatisect leaves and with upper corolla lips that are arch-shaped, and always hairy or fringed-incised, but not laterally compressed or flattened, and have pericarps lacking a sclerenchyma region [194]. *Phlomis* have a mostly circum-Mediterranean distribution, while the centers of diversification of *Phlomoides* include Central Asia, the Iranian highlands, and China [28, 142, 195].

#### Tribe Leonureae Dumort.

Leonureae were recircumscribed by Scheen et al. [52] and Bendiksby et al. [53] based on phylogenetic and morphologic data. They are comprised of 80 species in six genera: *Chaiturus* Willd. (1 sp.), *Lagochilus* Bunge ex Benth. (45 spp.), *Leonurus* L. (24 spp.), *Panzerina* Soják (2 spp.), *Loxocalyx* Hemsl. (3 spp.), and *Lagopsis* (Bunge ex Benth.) Bunge (5 spp.). The tribe is distributed primarily in Central Asia. Phylogenetic studies have shown that *Lagopsis* and *Leonurus* are poly- or paraphyletic [53]. Possible morphological synapomorphies for the tribe are short stamens included in the corolla tube and more or less palmate venation and lobing of the leaves. The genus *Loxocalyx* lacks these characters but shares zygomorphic calyces with longer abaxial lobes with many Leonureae.

#### Tribe Marrubieae Vis.

Marrubieae, with about 91 species, consist mostly of non-aromatic herbs or subshrubs, with thyrsoid inflorescences, few- to many-flowered cymes, widely campanulate to rotate calyces often with secondary calyx lobes, zygomorphic and 2-lipped corollas, and included or shortly exserted stamens [1]. The tribe is distributed from Europe to west and central Asia as well as North and South Africa with the highest number of species in southern Europe and North Africa [33].

The taxonomy and generic delimitations within Marrubieae have been controversial [33, 52, 53, 196–198]. Marrubieae contained three genera, *Ballota* L., *Marrubium* L., and *Moluccella* L. based on Scheen et al. [52]. Later, Bendiksby et al. [53] showed that the two species of *B.* sect. *Acanthoprasium* Benth. (*B. integrifolia* Benth., *B. frutescens* (L.) Woods) form a clade separate from the remaining species of *Ballota*. Therefore, Bendiksby et al. [53] resurrected the genus *Acanthoprasium* as proposed (but not formalized) by Scheen et al. [52]. The monotypic *Sulaimania* Hedge & Rech. f. was recovered as a member of the *Moluccella* clade and reduced to synonymy of *Moluccella* [53]. In a recent phylogenetic study of tribe Marrubieae using four plastid and one nuclear DNA locus (ITS), *B.* sect. *Beringeria* (Neck.) Benth. was raised to generic rank, as *Pseudodictamnus* Fabr. [33]. Therefore, the tribe now comprises five genera: *Acanthoprasium* (2 spp.), *Ballota* (3 spp.), *Marrubium* (ca. 50) spp., *Moluccella* (8 spp.), and *Pseudodictamnus* (28 spp.) [33].

Members of the genus *Acanthoprasium* are shrubby and woody, have long spiny bracteoles, and occur in Europe, while species of *Pseudodictamnus* are herbaceous, have leafy bracteoles, and are predominantly Mediterranean-African in distribution [33, 91]. *Ballota* as now circumscribed includes herbaceous species covered by simple trichomes and are distributed from Europe to West Asia (including also the Mediterranean) [33]*. Marrubium* was also recircumscribed recently to include *B. deserti* (de Noé) Jury, Rejdali & A.J.K. Griffiths. There are around 50 species assigned to this genus, which are characterized by a bifid upper corolla lip and distributed from Macaronesia to temperate Eurasia.

#### Tribe Leucadeae Scheen & Ryding

Leucadeae were established by Scheen et al. [52] and include ca. 134 species in six genera: *Acrotome* Benth. ex Endl. (8 spp.), *Isoleucas* O. Schwartz (2 spp.), *Leonotis* (Pers.) R. Br. (9 spp.), *Leucas* R. Br. (ca. 100 spp.), *Otostegia* Benth. (ca. 8 spp.), and *Rydingia* (4 spp.). These genera are distributed from Africa through the Indian subcontinent to Queensland, Australia [199]. With a few exceptions, members of Leucadeae have a calyx that is distinctly zygomorphic with secondary lobes and a bearded margin of the upper lip of the corolla [52]. The latter character is also found in the genus *Phlomoide*s [52]. The monophyly of Leucadeae has been corroborated using low-copy nuclear data [69], although only a small but representative selection of species was included in this study.

One molecular phylogeny has included a wide representation of species from all six genera, but only cpDNA markers were analyzed [199]. The large genus *Leucas*, with more than 100 species occurring on dry or disturbed ground in tropical to southern Africa and tropical and subtropical parts of Asia [1], was shown to be paraphyletic with respect to *Acrotome* and *Leonotis*, *Isoleucas*, and *Otostegia* [199]. Only a few of the Asian species of *Leucas* were included, but they formed a clade separate from the remaining *Leucas* [199]. More data are needed, including low-copy nuclear markers, before taxonomic changes can be proposed.

The genus *Otostegia*, as traditionally circumscribed, was clearly polyphyletic [199]. To make *Otostegia* monophyletic, the genus *Rydingia* was described to accommodate four Asian species, one species was transferred to *Isoleucas*, and one species was transferred to *Moluccella* [141]. Since then, an additional four species of *Otostegia* have also been transferred to *Moluccella* [53] (see also the discussion on tribe Marrubieae). Thus, the recircumscribed *Otostegia* is reduced to ca. eight species, most of which are endemic to Africa [53], with *O. fruticosa* (Forssk.) Schweinf. ex Penz. extending to the Arabian Peninsula [200].

Molecular phylogenies have resolved *Rydingia* as sister to the rest of Leucadeae, with this relationship recovered based on cpDNA [52, 53] and low-copy nuclear DNA [69]. However, more data are still needed to resolve the generic boundaries of the paraphyletic genus *Leucas* in relation to *Acrotome*, *Isoleucas*, *Leonotis*, and *Otostegia*.

#### Tribe Lamieae Coss. & Germ.

Lamieae are comprised of four genera: *Lamium* (including *Wiedemannia* Fisch. & C.A. Mey and *Lamiastrum*; ca. 25 spp.), *Eriophyton* Benth. (including *Alajja*; ca. 8 spp.), *Stachyopsis* (4 spp.), and possibly *Menitskia* (Krestovsk.) Krestovsk. (1 sp.). These genera are widely distributed in the temperate and subtropical regions of Europe, Asia, and Northern Africa. Five East Asian species of *Galeobdolon* and *Lamium chinense* Benth. were transferred into the genus *Matsumurella* in tribe Paraphlomideae by Bendiksby et al. [53]. Possible morphological synapomorphies for the tribe are hairy anthers (except for *Lamium galeobdolon* L., *L. flexuosum* Ten., *L. orvala* L. and some other species in the genus *Eriophyton*) and nutlets subtruncate or truncate at apex.

Ryding [201] included *Wiedemannia* within *Lamium*, and Harley et al. [1] included *Lamiastrum* (syn. *Galeobdolon*) in *Lamium*. Scheen et al. [52] defined Lamieae to consist of a single genus *Lamium* (including *Lamiastrum* and *Wiedemannia*). Scheen et al. [52] and Bendiksby et al. [202] both found a clade comprised of *Lamium*, *Lamiastrum*, and *Wiedemannia*, but did not have sufficient sampling to assess monophyly of *Lamium*, e.g., if *Lamiastrum* and *Wiedemannia* were excluded. Subsequent studies, with more complete sampling of *Lamium*, found *Lamiastrum* to be nested within *Lamium* [203, 204].

Bendiksby et al. [53] also determined that two other genera, *Eriophyton* (including *Alajja* and three species of *Lamium*) and *Stachyopsis*, should be included in tribe Lamieae. Bendiksby et al. [143] found that *Stachys tibetica* Vatke (= *Menitskia tibetica* (Vatke) Krestovsk.) did not belong in *Stachys* (tribe Stachydeae), but was most closely related to *Stachyopsis* in Lamieae. Morphologically, however, *S. tibetica* has an intermediate position between *Stachyopsis* and *Eriophyton*. They expanded *Eriophyton* to include *Stachyopsis* and *S*. *tibetica*, in order to make *Eriophyton* monophyletic. Lazkov and Sennikov [176] stated that the genus *Stachyopsis* is similar to *Eriophyton* but differs in the habit, shape of leaves (oblong-ovate vs. broadly rhomboid-ovate), and shorter flower tube which is enclosed within the calyx; therefore, they suggested that the genus *Stachyopsis* should retain its generic status. At the same time, they resurrected *Menitskia* to accommodate *S. tibetica* as *Menitskia tibetica*. The genus *Menitskia* differs from *Eriophyton* and *Stachyopsis* by its narrower posterior corolla lip, stiffer bracteoles, and often deeply crenate to lobed leaves [143, 205].

### Taxonomic treatment

#### Colquhounieae

C.L. Xiang, Bo Li & R.G. Olmstead, **trib. nov.** Type: *Colquhounia* Wall.

Shrubs erect or ascending. Stems and branches terete, with simple and/or branched hairs. Leaves toothed, petiolate; inflorescence thyrsoid, pedunculate to subsessile; cymes 1–5-flowered. Calyx tubular-campanulate, 10-veined, 5-lobed, lobes often equal. Corolla strongly 2-lipped, 4-lobed (1/3), often purple, sometimes spotted; posterior lip moderately long, hooded with upcurved margins, anterior lip slightly subequally 3-lobed, corolla tube strongly dilated distally; stamens 4, not exserted from corolla, thecae ± confluent; stigma lobes unequally 2-cleft. Nutlets narrowly obovoid-oblong, winged at apex.

Colquhounieae consist of one genus and approximately five species, occurring from Nepal, across north India to southwest and central China and Vietnam.

#### Rotheceae

C.L. Xiang, Bo Li & R.G. Olmstead, **trib. nov.** Type: *Rotheca* Raf.

Shrubs, subshrubs, and perennial herbs. Leaves simple, opposite, or whorled with 3–4 leaves per node, often toothed. Flowers often in terminal and/or axillary cymes. Calyx actinomorphic, 5-lobed or truncate. Corolla ± zygomorphic, expanding abruptly on lower side only; 5 lobes ± unequal, anterior corolla lobe frequently much larger than the other four, limb in bud asymmetrical. Stamens 4, didynamous to subequal, long-exserted; anthers usually basifixed (occasionally approaching versatile). Ovary unlobed in flower but becoming imperfectly 4-lobed during fruit development. Style terminal, stigma lobes frequently unequal. Drupes (2–) 4-lobed, mesocarp ± fleshy, endocarp separated into 4 stones or 2 pairs of stones.

The tribe contain four genera, *Rotheca* (60 spp.), *Glossocarya* (13 spp.), *Discretitheca* (1 sp.), and *Karomia* (9 spp.), and are distributed in tropical southern Asia to southern Africa, and Australia (Queensland).

#### Betoniceae

Bendiksby & Salmaki, **trib. nov.** Type: *Betonica* L.

Perennial herbs. Leaves deeply crenate-dentate. Flowering stems unbranched, lateral to rootstock, verticillasters condensed (rarely remote), 16–20-flowered. Bracteoles scarious or herbaceous, apex spinescent, base broad and hardened. Flowers sessile, median lobe of lower corolla lip emarginate. Calyx sessile, ± regular. Anther cells subparallel to parallel.

Betoniceae are monotypic comprising the genus *Betonica* with about 10 species distributed in western Eurasia.

## Conclusions

This is the first study to use plastome data to estimate family-wide relationships within Lamiaceae. We demonstrate that increased taxon sampling in concert with phylogenomic analyses based on plastome sequence data provides superior support and resolution at both deep and shallow nodes relative to previous studies and offers new insights into phylogenetic relationships among and between tribes and subfamilies of Lamiaceae. The monophyly of all 12 subfamilies is corroborated, and we recognize a total of 22 tribes within Lamiaceae, three of which are newly established here (i.e. Colquhounieae, Rotheceae, and Betoniceae). This study provides a detailed summary of the taxonomic history, generic and species diversity, morphology, synapomorphies, and distribution for each tribe and subfamily, representing the most comprehensive overview of Lamiaceae since Harley et al. [1]. The classification presented herein is the most definitive tribal-level taxonomy of the mint family to date, and the robust phylogenetic backbone of Lamiaceae reconstructed here provides an extendable dataset for future studies on mint family classification, biogeography, character evolution, and diversification.

## Materials and methods

### Taxon sampling

In this study, plastomes of 50 taxa were newly sequenced and 61 taxa were reassembled from the sequence read archive (SRA) database; others were acquired from previous studies [66, 67, 206, 207] or downloaded from NCBI (https://www.ncbi.nlm.nih.gov; Additional file 8: Table S3). In total, the ingroup sampling included 170 taxa (175 accessions), 79 genera, and represented all 15 currently recognized tribes and all 12 subfamilies within Lamiaceae [19, 51]. Twenty-two species from five families of Lamiales (Mazaceae, Orobanchaceae, Phrymaceae, Paulowniaceae, and Wightiaceae) were selected as outgroups based on phylogenetic results of previous studies [18, 22, 208]. Voucher specimens of the newly sequenced taxa (Table 1) were deposited at the Herbarium of Kunming Institute of Botany, Chinese Academy of Sciences (KUN).

### DNA isolation and sequencing

DNA was extracted from healthy and fresh leaves frozen in liquid nitrogen or dried in silica gel using the CTAB protocol of Doyle and Doyle [209] and sheared into ca. 300 bp fragments using a Covaris M220 Focused-ultrasonicator. Libraries for paired-end (PE) Illumina sequencing were constructed from fragmented genomic DNA following the standard protocol of manufacture (NEBNext® Ultra II™DNA Library Prep Kit for Illumina®) and sequenced from both ends of 150 bp fragments on the Illumina HiSeq 2000 platform (Illumina, San Diego, CA, USA) at BGI Genomics (BGO-Shenzhen, China). Approximately 2–10 GB of raw data was generated with 150 bp paired-end read lengths.

### Plastome assembly and annotation

Quality control of raw sequence reads was carried out using FastQC toolkit [210] (http://www.bioinformatics.babraham.ac.uk/projects/fastqc) with the parameter set as Q ≥ 25 to acquire high-quality clean reads. The de novo assembling of the plastome was implemented in the GetOrganelle pipeline [68], in which plastome reads were extracted from total genomic reads and then SPAdes v.3.10 [211] was used for assembly. For those plastomes we can acquire complete sequences, genome annotation was performed using Geneious v.11.0.3 [212], and the start and stop codons were manually adjusted by comparison with the plastome of *Salvia miltiorrhiza* Bunge [213] (HF586694). The online tRNAscan-SE web servers [214] were used to confirm the tRNA genes. Circular plastome maps were drawn using the OrganellarGenomeDRAW tool [215]. For 19 species, the plastomes were assembled from RNA-seq data and only contigs were obtained. Bowtie2 [216] was then used to map contigs to the reference sequences extracted from *S*. *miltiorrhiza* [213].

### Sequence alignment and dataset generation

Since noncoding regions can be variable even among species and are often difficult to align across a family as large as Lamiaceae, only 79 protein-coding genes were used for phylogenetic analyses. Alignments of individual loci were performed using the MAFFT v.7.308 [217] plugin in Geneious v.11.0.3 [212] with G-INS-I algorithm, and the final alignments were manually adjusted in PhyDE v.0.9971 [218].

Since the plastome is uniparentally inherited in most angiosperms and generally does not undergo recombination, sequences of the 79 coding genes were concatenated in our study to generate a supermatrix of all coding regions (CR). Removal of problematic aligned regions may result in a better resolved phylogeny [219]; therefore, ambiguously aligned positions (e.g., characters of uncertain homology among taxa and single-taxon insertions; see [31, 46]) were removed manually in our analyses to construct the “Coding region manual” dataset (CRM, Additional file 3: Table S2).

Additional matrices for the 79 genes were constructed based on (1) the 1st and 2nd codon positions (CR12); (2) only the 3rd codon positions (CR3); and (3) the degenerated coded sequences (dePCS) generated using Degen v.1.4 (http://www.phylotools.com/). Thus, a total of five datasets (CR, CRM, CR12, CR3, dePCS) were used in subsequent analyses.

### Phylogenetic analyses

Phylogenetic trees based on all datasets were built by two approaches including Bayesian inference (BI) analysis and maximum likelihood (ML) analysis. jModelTest v.2.1.4 [220] was used to determine the best-fit models for nucleotide sequences for BI analyses.

Bayesian analyses were executed using MrBayes v.3.2.2 [221]. Four iterations of 50,000,000 generations were run on four chains, sampling every 1000 generations on the Cyberinfrastructure for Phylogenetic Research Science (CIPRES) Gateway v.3.3 server [222] (http://www.phylo.org/). Default priors, unlinked parameter estimates, and best-fit models suggested by jModelTest v.2.1.4 [220] for each dataset were used for each iteration. Convergence of runs was accepted when the average standard deviation of split frequencies (ASDSF) dropped below 0.01. Tracer v.1.6.0 [223] was used to inspect the convergence of model parameters and check whether the values of effective sample size (ESS) were ≥ 200. A majority-rule consensus tree was created from the runs, after a 25% burn-in. All resulting trees with nodal support values were visualized and edited in FigTree v.1.4.2 [224].

ML analyses were performed using RAxML v.8.2.9 [225] as implemented in the XSEDE interface of CIPRES [222]. The GTRCAT model was used for analyses and bootstrapping; bootstrap iterations (–#|–N) were set to 1000, and other parameters used the CIPRES default settings.

We defined branches with posterior probabilities (PP) < 0.90 and bootstrap values (BS) < 70% as weakly supported, PP = 0.90–0.95 and BS = 70%–80% as moderately supported, and PP ≥ 0.95 and BS ≥ 80% as strongly supported [107]. The alignments and ML tree are deposited at TreeBase with study #S26639 (http://treebase.org/treebase-web/phylows/study/TB2:S26639?x-access-code=bb02a4c5bc226f4604690ea0f21ccd41&format=html) [226].

## Supplementary Information

**Additional file 1: Figure S1.** Gene maps of the complete chloroplast genomes newly sequenced in this study. Genes inside and outside of the circle are transcribed in the clockwise and counterclockwise directions, respectively. Genes belonging to different functional categories are color-coded.

**Additional file 2: Table S1.** Features of newly sequenced plastomes.

**Additional file 3: Table S2.** Excluded ambiguous sites for 79 genes of coding regions (dataset CRM).

**Additional file 4: Figure S2.** Phylograms inferred from ML analysis of concatenated nucleotide sequences of 79 protein-coding genes (dataset CR). A, phylogram showing branch lengths, where tips names are absent follow the same order as shown in B. Scale bar represents the mean number of nucleotide substitutions per site. B, maximum likelihood bootstrap support values and Bayesian inference posterior probabilities are shown above and below the branches, respectively.

**Additional file 5: Figure S3.** Phylograms inferred from ML analysis of concatenated nucleotide sequences of the 3^rd^ codon positions (dataset CR3). A, phylogram showing branch lengths, where tip names are absent follow the same order as shown in B. Scale bar represents the mean number of nucleotide substitutions per site. B, maximum likelihood bootstrap support values and Bayesian inference posterior probabilities are shown above and below the branches, respectively.

**Additional file 6: Figure S4.** Phylograms inferred from ML analysis of concatenated nucleotide sequences of the 1^st^ and 2^nd^ codon positions (dataset CR12). A, phylogram showing branch lengths, where tips names are absent follow the same order as shown in B. Scale bar represents the mean number of nucleotide substitutions per site. B, maximum likelihood bootstrap support values and Bayesian inference posterior probabilities are shown above and below the branches, respectively.

**Additional file 7: Figure S5.** Phylograms inferred from ML analysis of concatenated nucleotide sequences of the degeneracy nucleotide sequence (dataset dePCS). A, phylogram showing branch lengths, where tip names are absent follow the same order as shown in B. Scale bar represents the mean number of nucleotide substitutions per site. B, maximum likelihood bootstrap support values are shown above the branches.

**Additional file 8: Table S3.** List of taxa sampled with information related to taxonomy, GenBank accession numbers, references, and vouchers. Herbarium acronyms follow Index Herbariorum [227].

## Data Availability

All the newly sequenced and annotated plastomes in the present study were submitted to the National Center for Biotechnology Information (NCBI) database with accession numbers MT473738–MT473786 (Table 1). Other plastomes analyzed were acquired from previous studies [66, 67, 206, 207] or downloaded from NCBI (https://www.ncbi.nlm.nih.gov; Additional file 8; Table S3). The alignments and ML tree are deposited at TreeBase with study #S26639 (http://treebase.org/treebase-web/phylows/study/TB2:S26639?x-access-code=bb02a4c5bc226f4604690ea0f21ccd41&format=html) [226].
