# Supplementary material for: An updated tribal classification of Lamiaceae based on plastome phylogenomics
Source: BMC Biol. 2021 Jan 8;19:2. doi: 10.1186/s12915-020-00931-z (PMC7796571; doi:10.1186/s12915-020-00931-z)
Supplement: Supplementary file 1 — Additional file 1: Figure S1. Gene maps of the complete chloroplast genomes newly sequenced in this study. Genes inside and outside of the circle are transcribed in the clockwise and counterclockwise directions, respectively. Genes belonging to different functional categories are color-coded. [file 12915_2020_931_MOESM1_ESM.pdf]

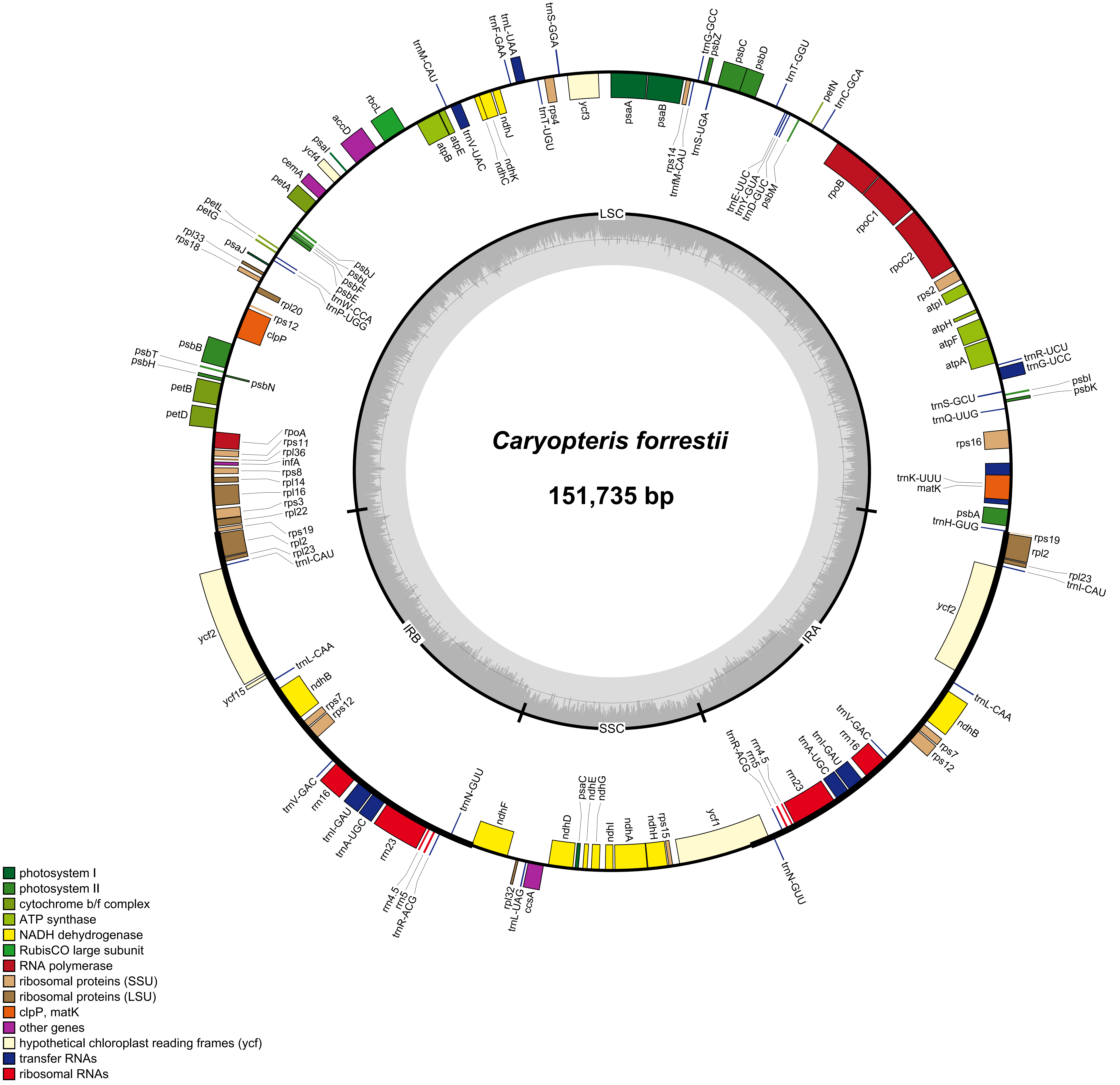
Caryopteris forrestii
151,735 bp
LSC
IRA
IRB
SSC
trnH-GUG
psbA
matK
trnK-UUU
rps16
trnQ-UUG
trnS-GCU
psbK
psbI
trnG-UCC
trnR-UCU
atpA
atpF
atpH
atpI
rps2
rpoC2
rpoC1
rpoB
trnC-GCA
petN
psbM
trnD-GUC
trnY-GUA
trnE-UUC
trnT-GGU
psbD
psbC
trnS-UGA
psbZ
trnG-GCC
trnfM-CAU
rps14
psaB
psaA
ycf3
trnS-GGA
rps4
trnT-UGU
trnL-UAA
trnF-GAA
ndhJ
ndhK
ndhC
trnV-UAC
trnM-CAU
atpE
atpB
rbcL
accD
psaI
ycf4
cemA
petA
psbJ
psbL
psbF
psbE
petL
petG
trnW-CCA
trnP-UGG
psaJ
rpl33
rps18
rpl20
rps12
clpP
psbB
psbT
psbN
psbH
petB
petD
rpoA
rps11
rpl36
infA
rps8
rpl14
rpl16
rps3
rpl22
rps19
rpl2
rpl23
trnI-CAU
ycf2
trnL-CAA
ndhB
rps7
ycf15
trnV-GAC
rrn16
trnI-GAU
trnA-UGC
rrn23
rrn4.5
rrn5
trnR-ACG
trnN-GUU
ndhF
rpl32
trnL-UAG
ccsA
ndhD
psaC
ndhE
ndhG
ndhI
ndhA
ndhH
rps15
ycf1
photosystem I
photosystem II
cytochrome b/f complex
ATP synthase
NADH dehydrogenase
RubisCO large subunit
RNA polymerase
ribosomal proteins (SSU)
ribosomal proteins (LSU)
clpP, matK
other genes
hypothetical chloroplast reading frames (ycf)
transfer RNAs
ribosomal RNAs

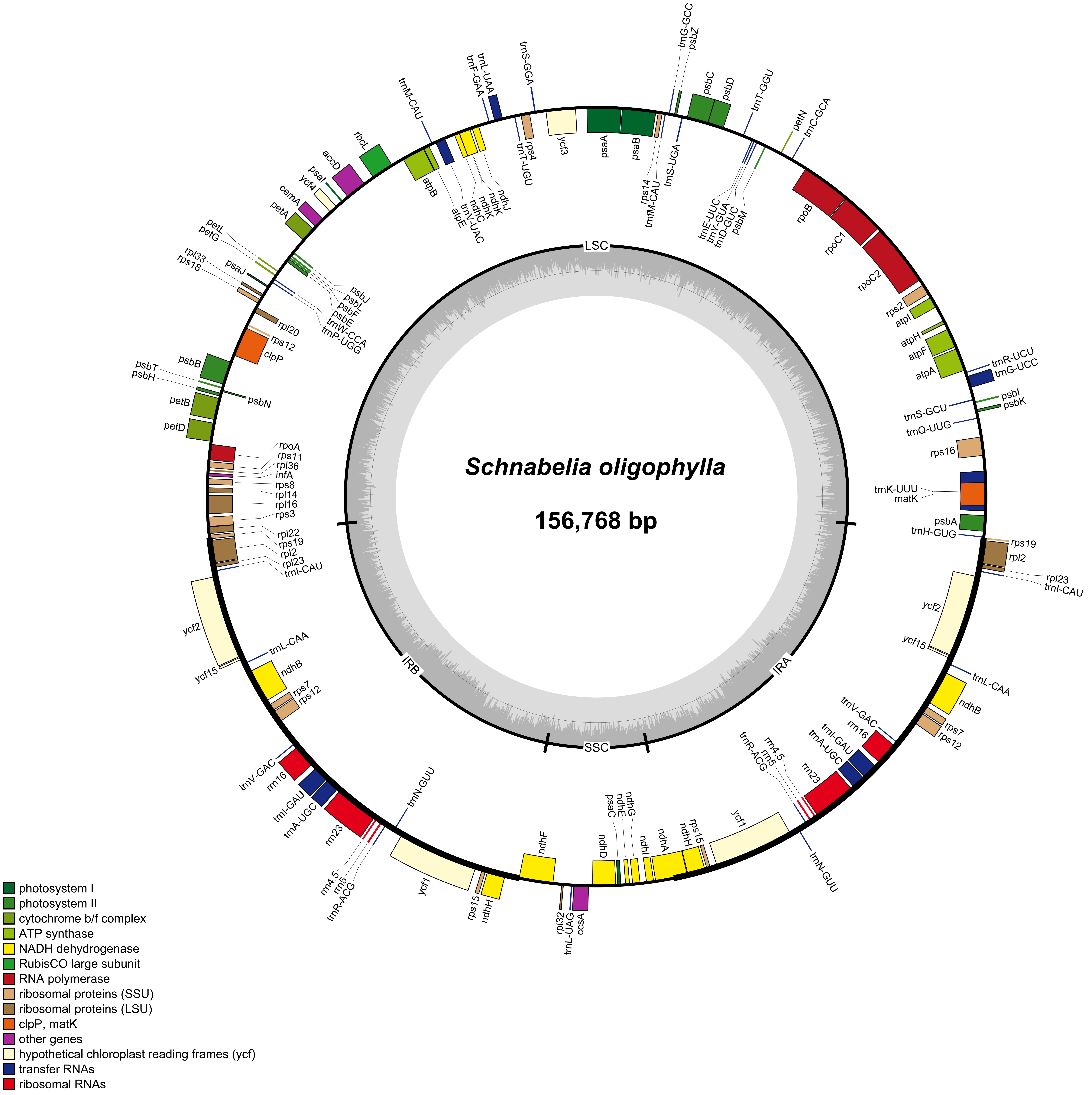

Schnabelia oligophylla
156,768 bp
LSC
SSC
IRA
IRB
trnG-GCC
psbZ
psbC
psbD
trnT-GGU
petN
trnC-GCA
rpoB
rpoC1
rpoC2
rps2
atpI
atpH
atpF
atpA
trnR-UCU
trnG-UCC
psbI
psbK
trnS-GCU
trnQ-UUG
rps16
trnK-UUU
matK
psbA
trnH-GUG
rps19
rpl2
rpl23
trnI-CAU
ycf2
ycf15
trnL-CAA
ndhB
rps7
rps12
trnV-GAC
rrn16
trnI-GAU
trnA-UGC
rrn23
rrn4.5
rrn5
trnR-ACG
trnN-GUU
ycf1
rps15
ndhH
ndhA
ndhI
ndhG
ndhE
psaC
ndhD
ccsA
trnL-UAG
rpl32
ndhF
trnS-GGA
trnL-UAA
trnF-GAA
trnM-CAU
rbcL
accD
psaI
ycf4
cemA
petA
petL
petG
rpl33
rps18
psaJ
psbJ
psbL
psbF
psbE
trnW-CCA
trnP-UGG
rpl20
rps12
clpP
psbB
psbT
psbH
psbN
petB
petD
rpoA
rps11
rpl36
infA
rps8
rpl14
rpl16
rps3
rpl22
rps19
rpl2
rpl23
trnI-CAU
atpB
atpE
trnV-UAC
ndhC
ndhK
ndhJ
trnT-UGU
rps4
ycf3
psaA
psaB
rps14
trnfM-CAU
trnS-UGA
trnE-UUC
trnY-GUA
trnD-GUC
psbM
photosystem I
photosystem II
cytochrome b/f complex
ATP synthase
NADH dehydrogenase
RubisCO large subunit
RNA polymerase
ribosomal proteins (SSU)
ribosomal proteins (LSU)
clpP, matK
other genes
hypothetical chloroplast reading frames (ycf)
transfer RNAs
ribosomal RNAs

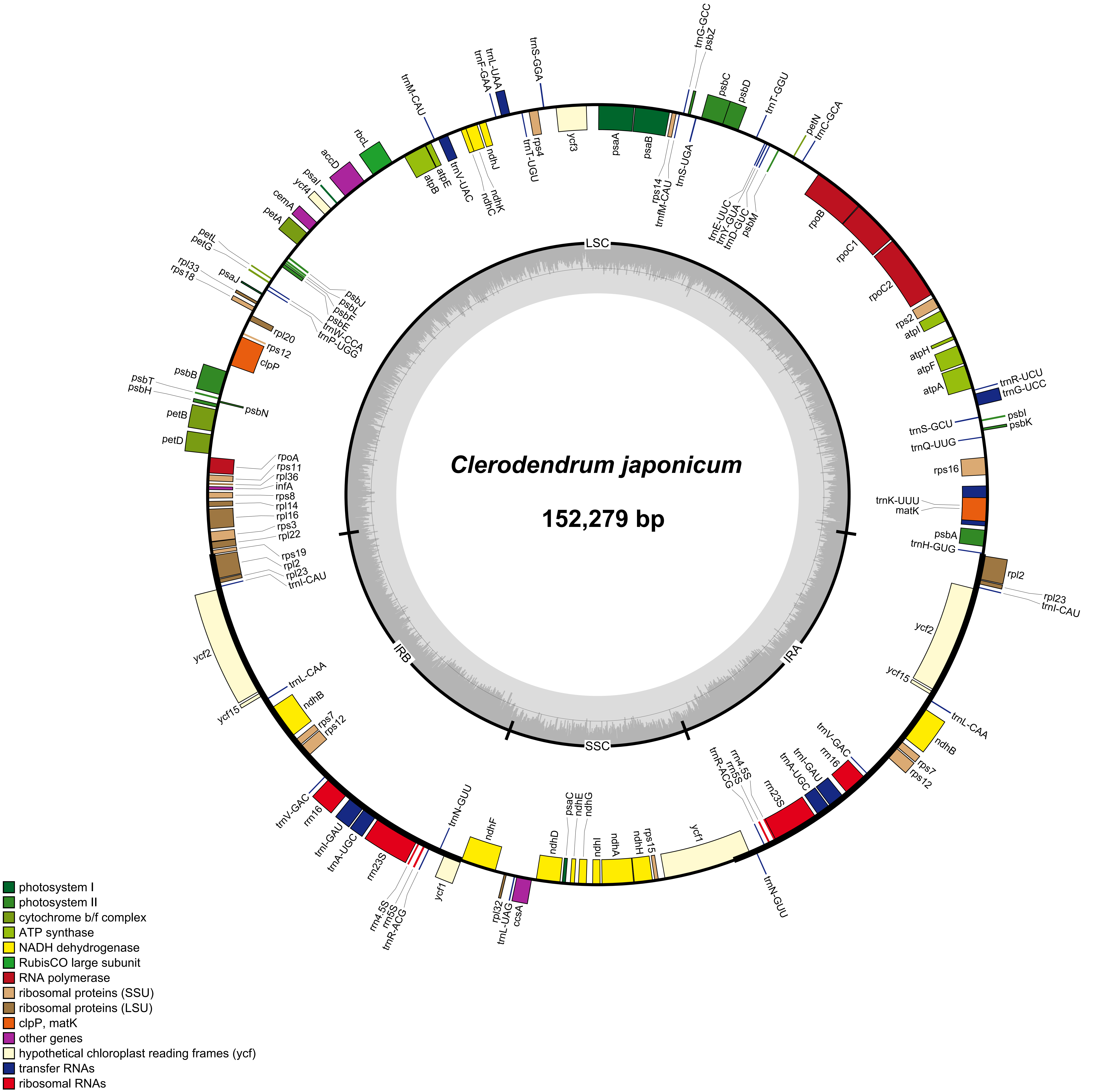

Clerodendrum japonicum
152,279 bp
LSC
SSC
IRA
IRB
photosystem I
photosystem II
cytochrome b/f complex
ATP synthase
NADH dehydrogenase
RubisCO large subunit
RNA polymerase
ribosomal proteins (SSU)
ribosomal proteins (LSU)
clpP, matK
other genes
hypothetical chloroplast reading frames (ycf)
transfer RNAs
ribosomal RNAs

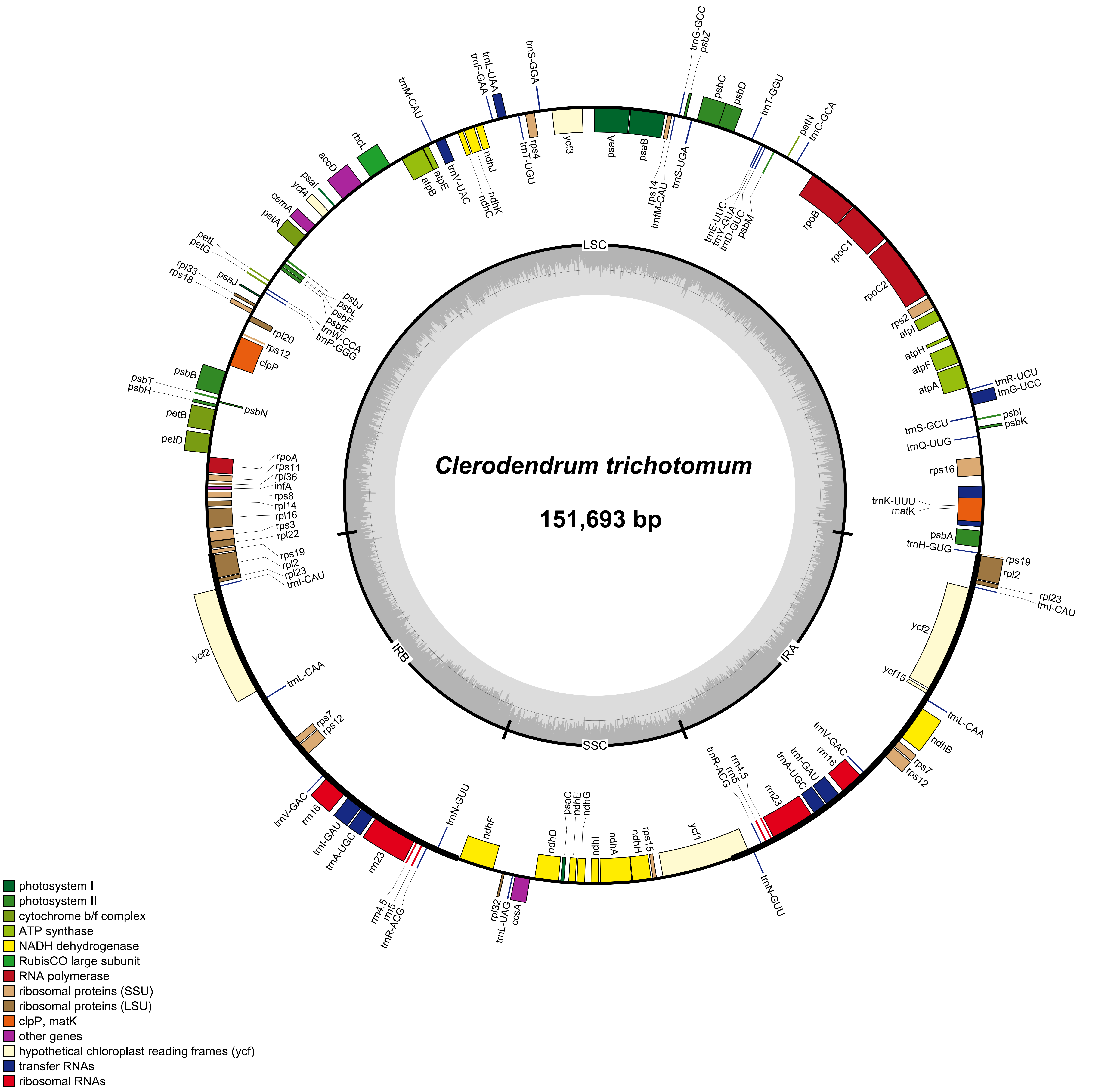
Clerodendrum trichotomum
151,693 bp
LSC
IRA
SSC
IRB
trnG-GCC
psbZ
psbC
psbD
trnT-GGU
petN
trnC-GCA
psaA
psaB
rps14
trnfM-CAU
trnS-UGA
trnE-UUC
trnY-GUA
trnD-GUC
psbM
rpoB
rpoC1
rpoC2
rps2
atpI
atpH
atpF
atpA
trnR-UCU
trnG-UCC
psbI
psbK
trnS-GCU
trnQ-UUG
rps16
trnK-UUU
matK
psbA
trnH-GUG
rps19
rpl2
rpl23
trnI-CAU
ycf2
ycf15
trnL-CAA
ndhB
rps7
rps12
trnV-GAC
rrn16
trnI-GAU
trnA-UGC
rrn23
rrn4.5
rrn5
trnR-ACG
trnN-GUU
ycf1
rps15
ndhH
ndhA
ndhI
ndhG
ndhE
psaC
ndhD
ccsA
trnL-UAG
rpl32
ndhF
trnS-GGA
rps4
trnT-UGU
ycf3
trnL-UAA
trnF-GAA
ndhJ
ndhK
ndhC
trnV-UAC
trnM-CAU
atpE
atpB
rbcL
accD
psaI
ycf4
cemA
petA
petL
petG
psbJ
psbL
psbF
psbE
trnW-CCA
trnP-GGG
psaJ
rpl33
rps18
rpl20
clpP
psbB
psbT
psbH
psbN
petB
petD
rpoA
rps11
rpl36
infA
rps8
rpl14
rpl16
rps3
rpl22
photosystem I
photosystem II
cytochrome b/f complex
ATP synthase
NADH dehydrogenase
RubisCO large subunit
RNA polymerase
ribosomal proteins (SSU)
ribosomal proteins (LSU)
clpP, matK
other genes
hypothetical chloroplast reading frames (ycf)
transfer RNAs
ribosomal RNAs

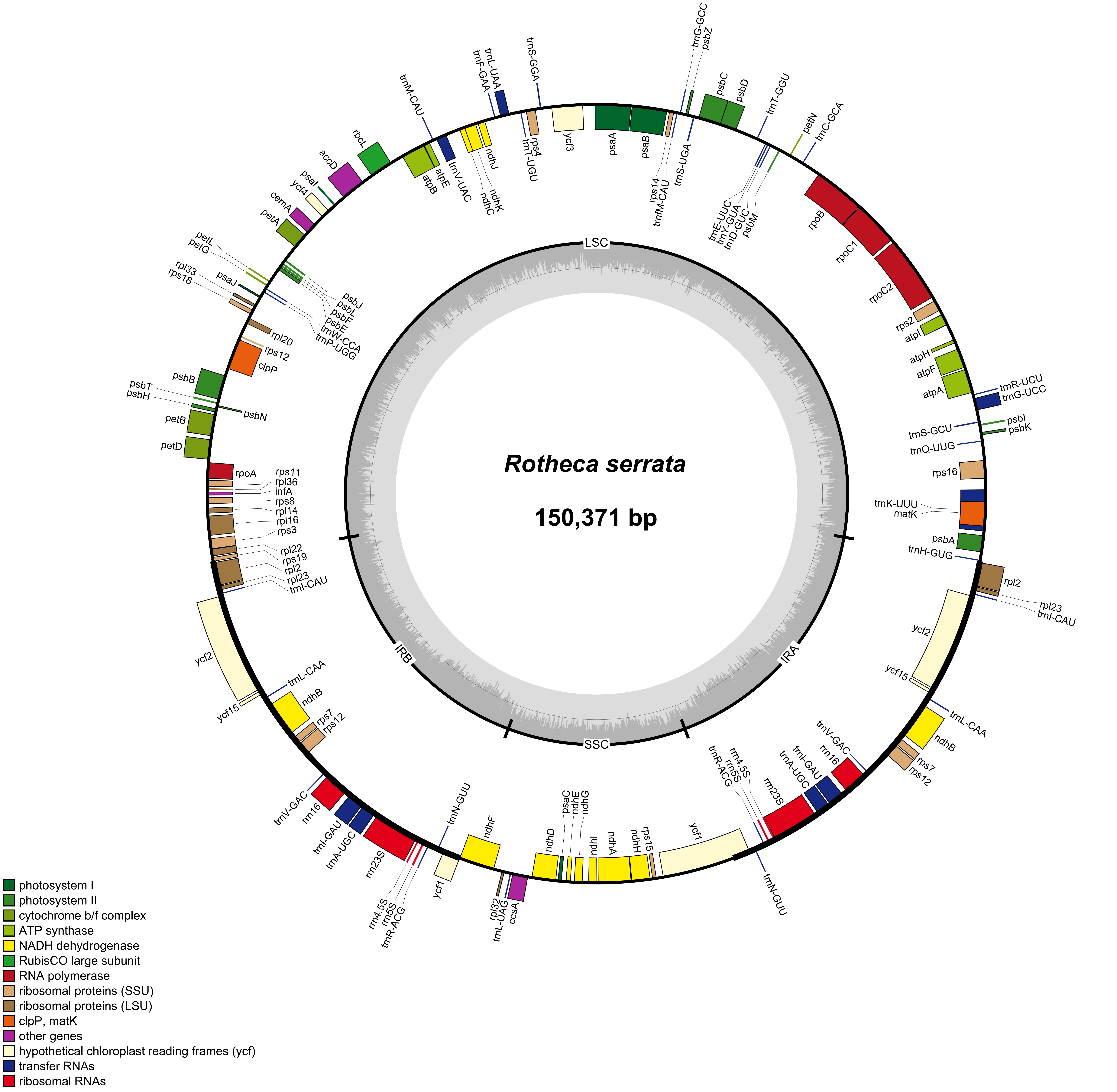

Rotheca serrata
150,371 bp
LSC
SSC
IRA
IRB
trnG-GCC
psbZ
psbC
psbD
trnT-GGU
petN
trnC-GCA
trnS-GGA
trnL-UAA
trnF-GAA
trnM-CAU
rbcL
accD
psaI
ycf4
cemA
petA
petL
petG
rpl33
rps18
psaJ
psbJ
psbL
psbF
psbE
trnW-CCA
trnP-UGG
rpl20
rps12
clpP
psbB
psbT
psbH
psbN
petB
petD
rpoA
rps11
rpl36
infA
rps8
rpl14
rpl16
rps3
rpl22
rps19
rpl2
rpl23
trnI-CAU
ycf2
ycf15
trnL-CAA
ndhB
rps7
trnV-GAC
rrn16
trnI-GAU
trnA-UGC
rrn23S
rrn4.5S
rrn5S
trnR-ACG
trnN-GUU
ndhF
ycf1
rpl32
trnL-UAG
ccsA
ndhD
psaC
ndhE
ndhG
ndhI
ndhA
ndhH
rps15
atpB
atpE
trnV-UAC
ndhC
ndhK
ndhJ
rps4
trnT-UGU
ycf3
psaA
psaB
rps14
trnfM-CAU
trnS-UGA
trnE-UUC
trnY-GUA
trnD-GUC
psbM
rpoB
rpoC1
rpoC2
rps2
atpI
atpH
atpF
atpA
trnR-UCU
trnG-UCC
psbI
psbK
trnS-GCU
trnQ-UUG
rps16
trnK-UUU
matK
psbA
trnH-GUG
photosystem I
photosystem II
cytochrome b/f complex
ATP synthase
NADH dehydrogenase
RubisCO large subunit
RNA polymerase
ribosomal proteins (SSU)
ribosomal proteins (LSU)
clpP, matK
other genes
hypothetical chloroplast reading frames (ycf)
transfer RNAs
ribosomal RNAs

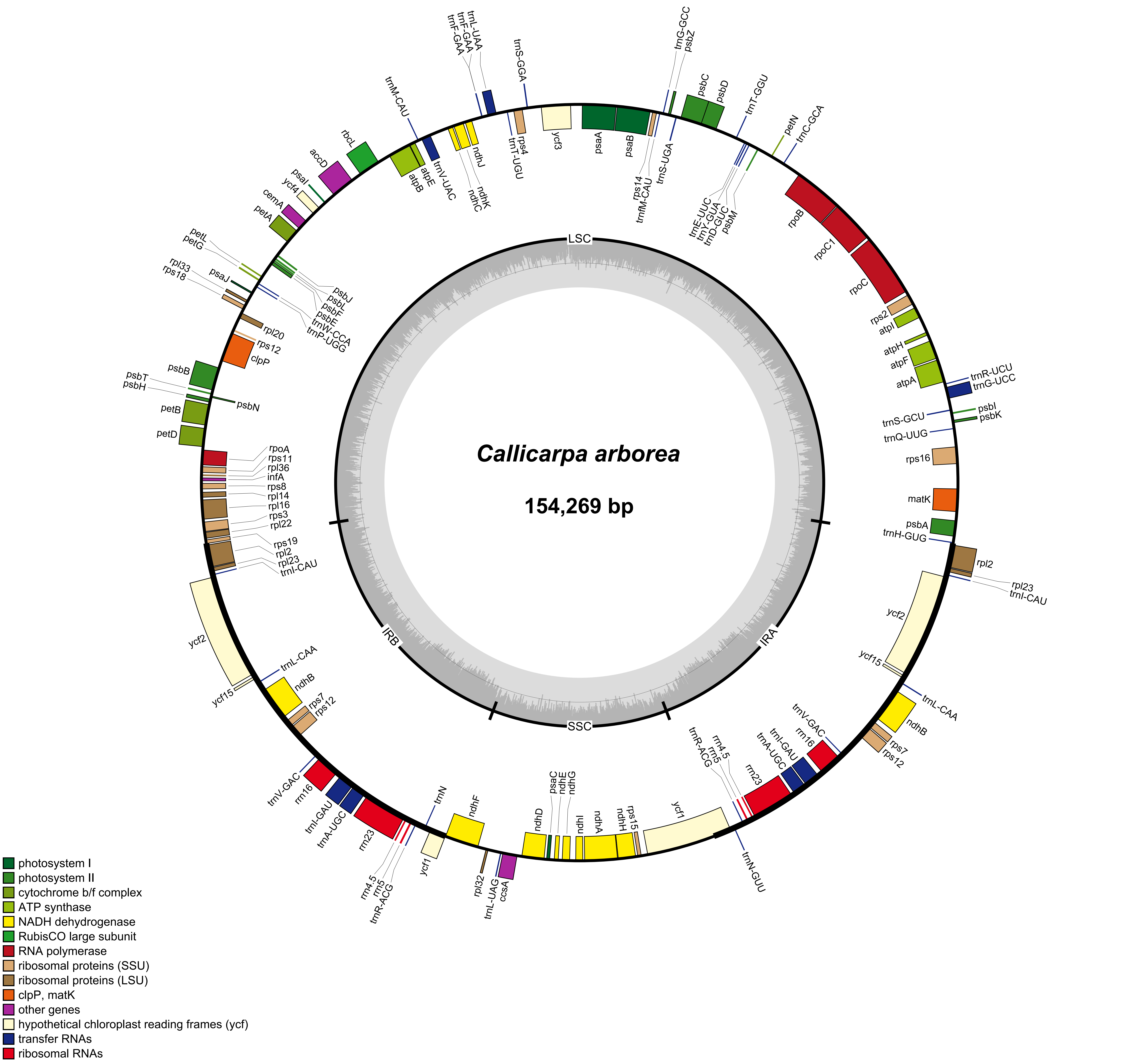
Callicarpa arborea
154,269 bp
LSC
IRA
SSC
IRB
photosystem I
photosystem II
cytochrome b/f complex
ATP synthase
NADH dehydrogenase
RubisCO large subunit
RNA polymerase
ribosomal proteins (SSU)
ribosomal proteins (LSU)
clpP, matK
other genes
hypothetical chloroplast reading frames (ycf)
transfer RNAs
ribosomal RNAs
psbA
trnH-GUG
matK
rps16
trnQ-UUG
trnS-GCU
psbI
psbK
trnG-UCC
trnR-UCU
atpA
atpF
atpH
atpI
rps2
rpoC2
rpoC1
rpoB
trnC-GCA
petN
psbM
trnD-GUC
trnY-GUA
trnE-UUC
trnT-GGU
psbD
psbC
psbZ
trnG-GCC
trnS-UGA
trnfM-CAU
rps14
psaB
psaA
ycf3
rps4
trnT-UGU
trnS-GGA
trnL-UAA
trnF-GAA
ndhJ
ndhK
ndhC
trnV-UAC
trnM-CAU
atpE
atpB
rbcL
accD
psaI
ycf4
cemA
petA
petL
petG
psbJ
psbL
psbF
psbE
trnW-CCA
trnP-UGG
psaJ
rpl33
rps18
rpl20
rps12
clpP
psbB
psbT
psbH
psbN
petB
petD
rpoA
rps11
rpl36
infA
rps8
rpl14
rpl16
rps3
rpl22
rps19
rpl2
rpl23
trnI-CAU
ycf2
ycf15
trnL-CAA
ndhB
rps7
trnV-GAC
rrn16
trnI-GAU
trnA-UGC
rrn23
rrn4.5
rrn5
trnR-ACG
trnN-GUU
ycf1
ndhF
rpl32
trnL-UAG
ccsA
ndhD
psaC
ndhE
ndhG
ndhI
ndhA
ndhH
rps15

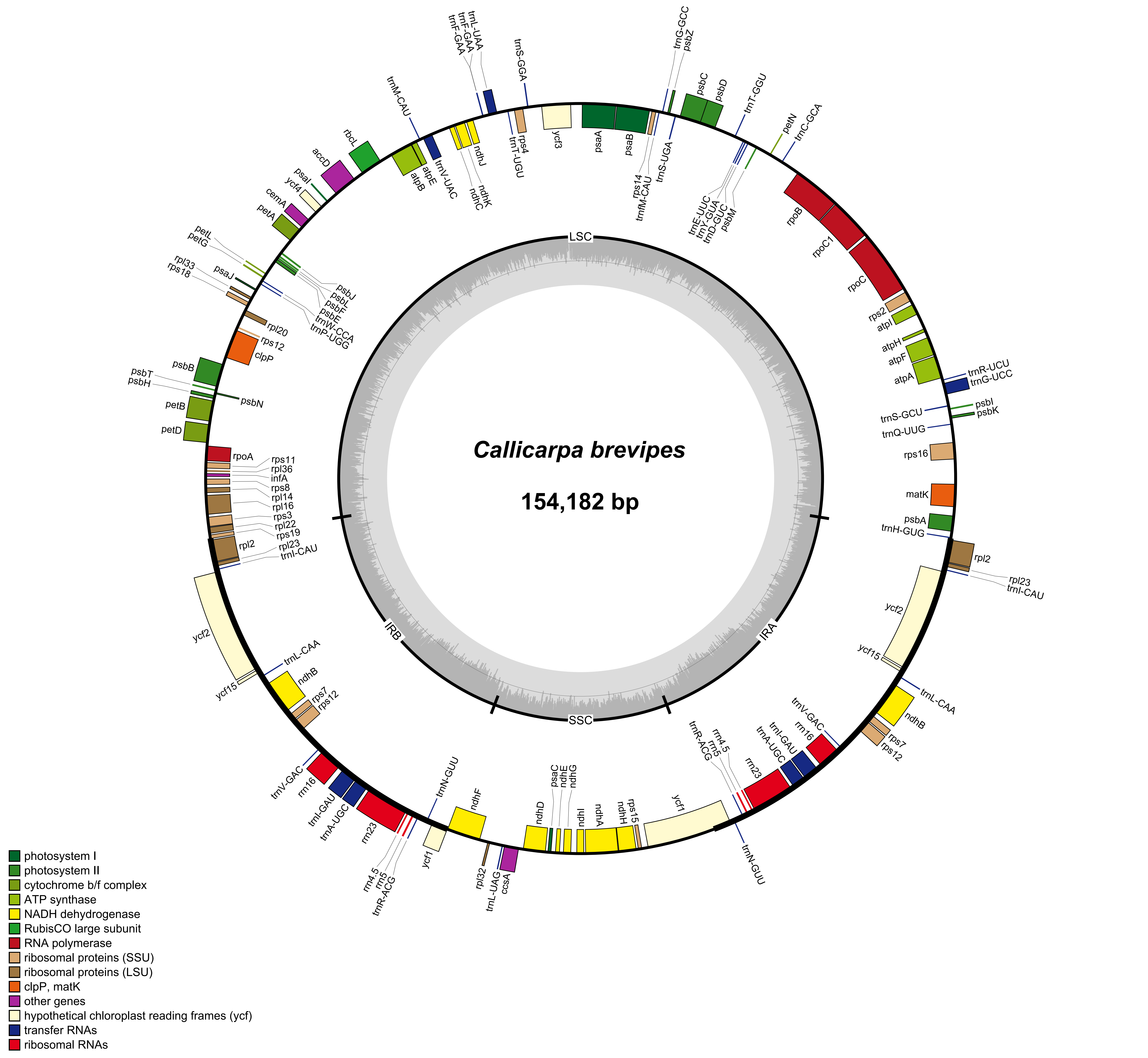

Callicarpa brevipes
154,182 bp
LSC
IRA
SSC
IRB
trnG-GCC
psbZ
psbC
psbD
trnT-GGU
petN
trnC-GCA
rpoB
rpoC1
rpoC
rps2
atpI
atpH
atpF
atpA
trnR-UCU
trnG-UCC
psbI
psbK
trnS-GCU
trnQ-UUG
rps16
matK
psbA
trnH-GUG
rpl2
rpl23
trnI-CAU
ycf2
ycf15
trnL-CAA
ndhB
rps7
rps12
trnV-GAC
rrn16
trnI-GAU
trnA-UGC
rrn23
rrn4.5
rrn5
trnR-ACG
trnN-GUU
ycf1
rps15
ndhH
ndhA
ndhI
ndhG
ndhE
psaC
ndhD
ccsA
trnL-UAG
rpl32
ndhF
rpoA
rps11
rpl36
infA
rps8
rpl14
rpl16
rps3
rpl22
rps19
petD
petB
psbH
psbT
psbB
psbN
clpP
rpl20
rps18
rpl33
psaJ
trnP-UGG
trnW-CCA
psbE
psbF
psbL
psbJ
petG
petL
petA
cemA
ycf4
psaI
accD
rbcL
atpB
atpE
trnV-UAC
trnM-CAU
ndhC
ndhK
ndhJ
trnF-GAA
trnL-UAA
trnS-GGA
trnT-UGU
rps4
ycf3
psaA
psaB
rps14
trnfM-CAU
trnS-UGA
trnE-UUC
trnY-GUA
trnD-GUC
psbM
photosystem I
photosystem II
cytochrome b/f complex
ATP synthase
NADH dehydrogenase
RubisCO large subunit
RNA polymerase
ribosomal proteins (SSU)
ribosomal proteins (LSU)
clpP, matK
other genes
hypothetical chloroplast reading frames (ycf)
transfer RNAs
ribosomal RNAs

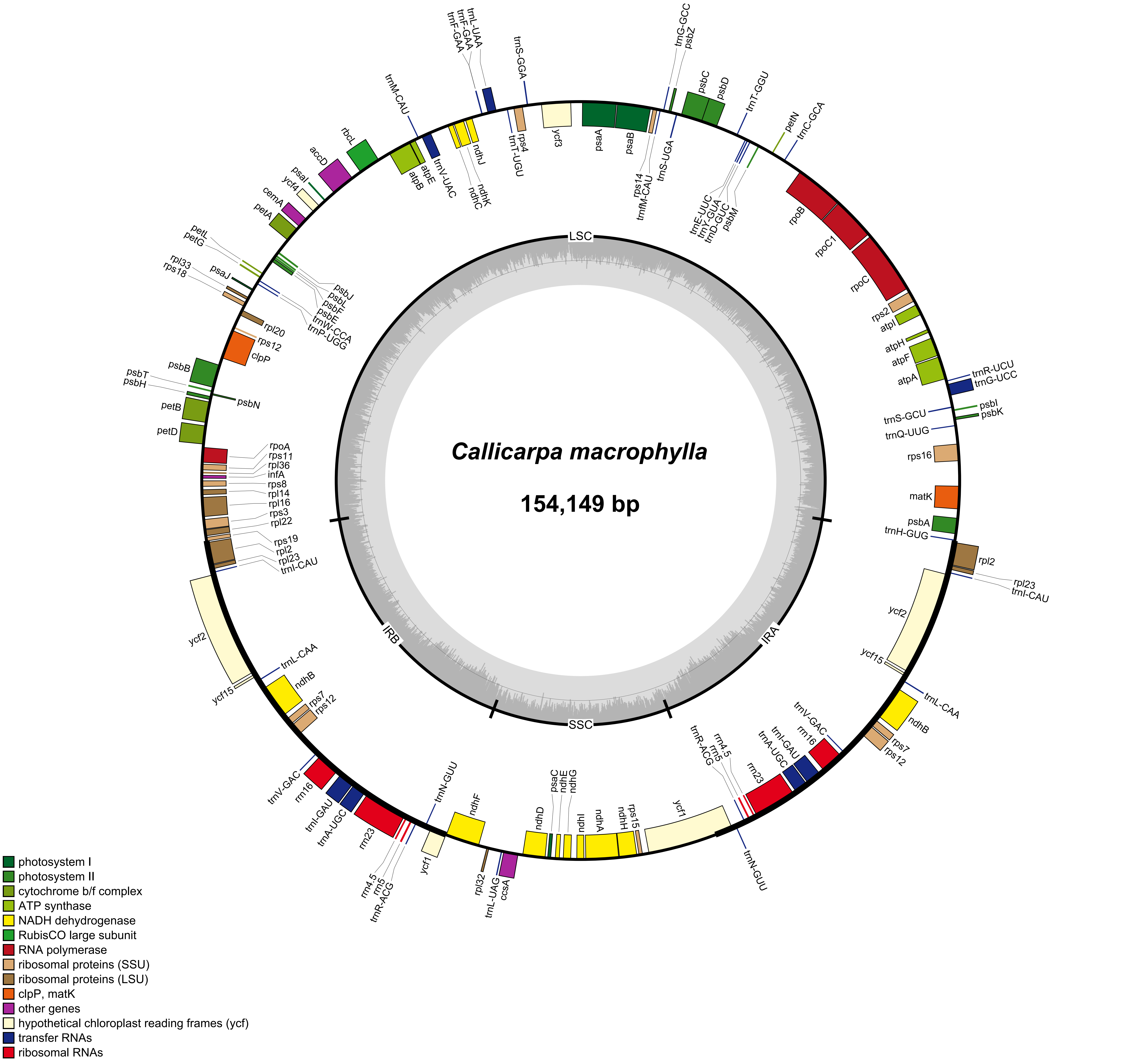
Callicarpa macrophylla
154,149 bp
LSC
IRA
SSC
IRB
trnG-GCC
psbZ
psbC
psbD
trnT-GGU
petN
trnC-GCA
trnS-GGA
trnL-UAA
trnF-GAA
trnF-GAA
trnM-CAU
rbcL
accD
psaI
ycf4
cemA
petA
petL
petG
rpl33
rps18
psaJ
rpl20
rps12
clpP
psbB
psbT
psbH
psbN
petB
petD
rpoA
rps11
rpl36
infA
rps8
rpl14
rpl16
rps3
rpl22
rps19
rpl2
rpl23
trnI-CAU
ycf2
ycf15
trnL-CAA
ndhB
rps7
rps12
trnV-GAC
rrn16
trnI-GAU
trnA-UGC
rrn23
rrn4.5
rrn5
trnR-ACG
trnN-GUU
ycf1
ndhF
rpl32
trnL-UAG
ccsA
ndhD
psaC
ndhE
ndhG
ndhI
ndhA
ndhH
rps15
ycf1
trnN-GUU
trnR-ACG
rrn5
rrn4.5
rrn23
trnA-UGC
trnI-GAU
rrn16
trnV-GAC
rps12
rps7
ndhB
trnL-CAA
ycf15
ycf2
rpl23
trnI-CAU
rpl2
trnH-GUG
psbA
matK
rps16
trnQ-UUG
trnS-GCU
psbI
psbK
trnR-UCU
trnG-UCC
atpA
atpF
atpH
atpI
rps2
rpoC
rpoC1
rpoB
trnE-UUC
trnY-GUA
trnD-GUC
psbM
trnS-UGA
trnfM-CAU
rps14
psaB
psaA
ycf3
rps4
trnT-UGU
ndhJ
ndhK
ndhC
trnV-UAC
atpE
atpB
psbJ
psbL
psbF
psbE
trnW-CCA
trnP-UGG
photosystem I
photosystem II
cytochrome b/f complex
ATP synthase
NADH dehydrogenase
RubisCO large subunit
RNA polymerase
ribosomal proteins (SSU)
ribosomal proteins (LSU)
clpP, matK
other genes
hypothetical chloroplast reading frames (ycf)
transfer RNAs
ribosomal RNAs

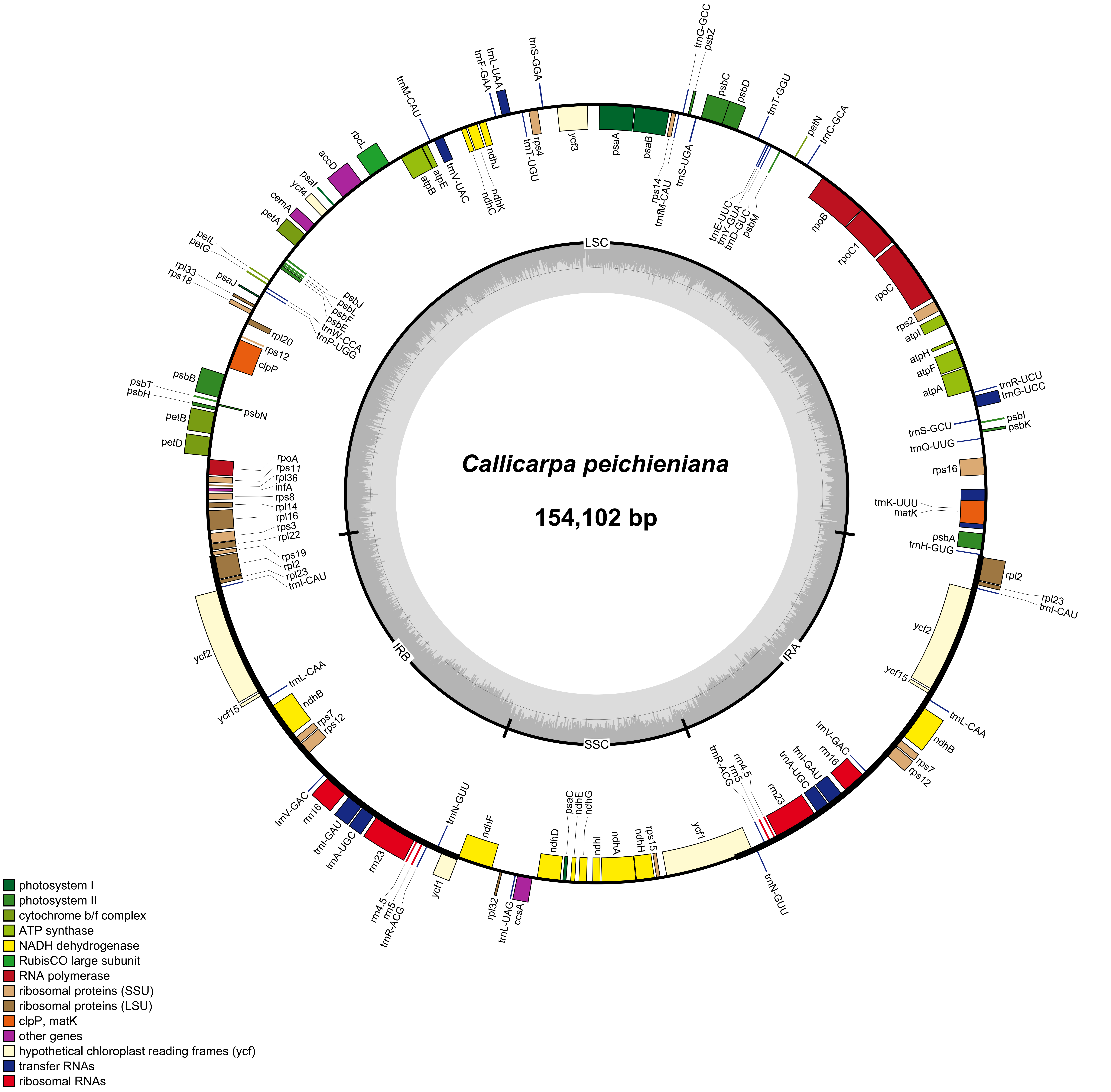
Callicarpa peichieniana
154,102 bp
LSC
IRA
IRB
SSC
trnG-GCC
psbZ
psbC
psbD
trnT-GGU
petN
trnC-GCA
trnS-GGA
trnL-UAA
trnF-GAA
trnM-CAU
rps4
trnT-UGU
ycf3
psaA
psaB
rps14
trnfM-CAU
trnS-UGA
trnE-UUC
trnY-GUA
trnD-GUC
psbM
rpoB
rpoC1
rpoC
rps2
atpI
atpH
atpF
atpA
trnR-UCU
trnG-UCC
psbI
psbK
trnS-GCU
trnQ-UUG
rps16
trnK-UUU
matK
psbA
trnH-GUG
rpl2
rpl23
trnI-CAU
ycf2
ycf15
trnL-CAA
ndhB
rps7
rps12
trnV-GAC
rrn16
trnI-GAU
trnA-UGC
rrn23
rrn4.5
rrn5
trnR-ACG
trnN-GUU
ycf1
rps15
ndhH
ndhA
ndhI
ndhG
ndhE
psaC
ndhD
ccsA
trnL-UAG
rpl32
ndhF
ndhJ
ndhK
ndhC
trnV-UAC
atpE
atpB
rbcL
accD
psaI
ycf4
cemA
petA
petL
petG
psaJ
rpl33
rps18
psbJ
psbL
psbF
psbE
trnW-CCA
trnP-UGG
rpl20
clpP
psbB
psbT
psbH
psbN
petB
petD
rpoA
rps11
rpl36
infA
rps8
rpl14
rpl16
rps3
rpl22
rps19
photosystem I
photosystem II
cytochrome b/f complex
ATP synthase
NADH dehydrogenase
RubisCO large subunit
RNA polymerase
ribosomal proteins (SSU)
ribosomal proteins (LSU)
clpP, matK
other genes
hypothetical chloroplast reading frames (ycf)
transfer RNAs
ribosomal RNAs

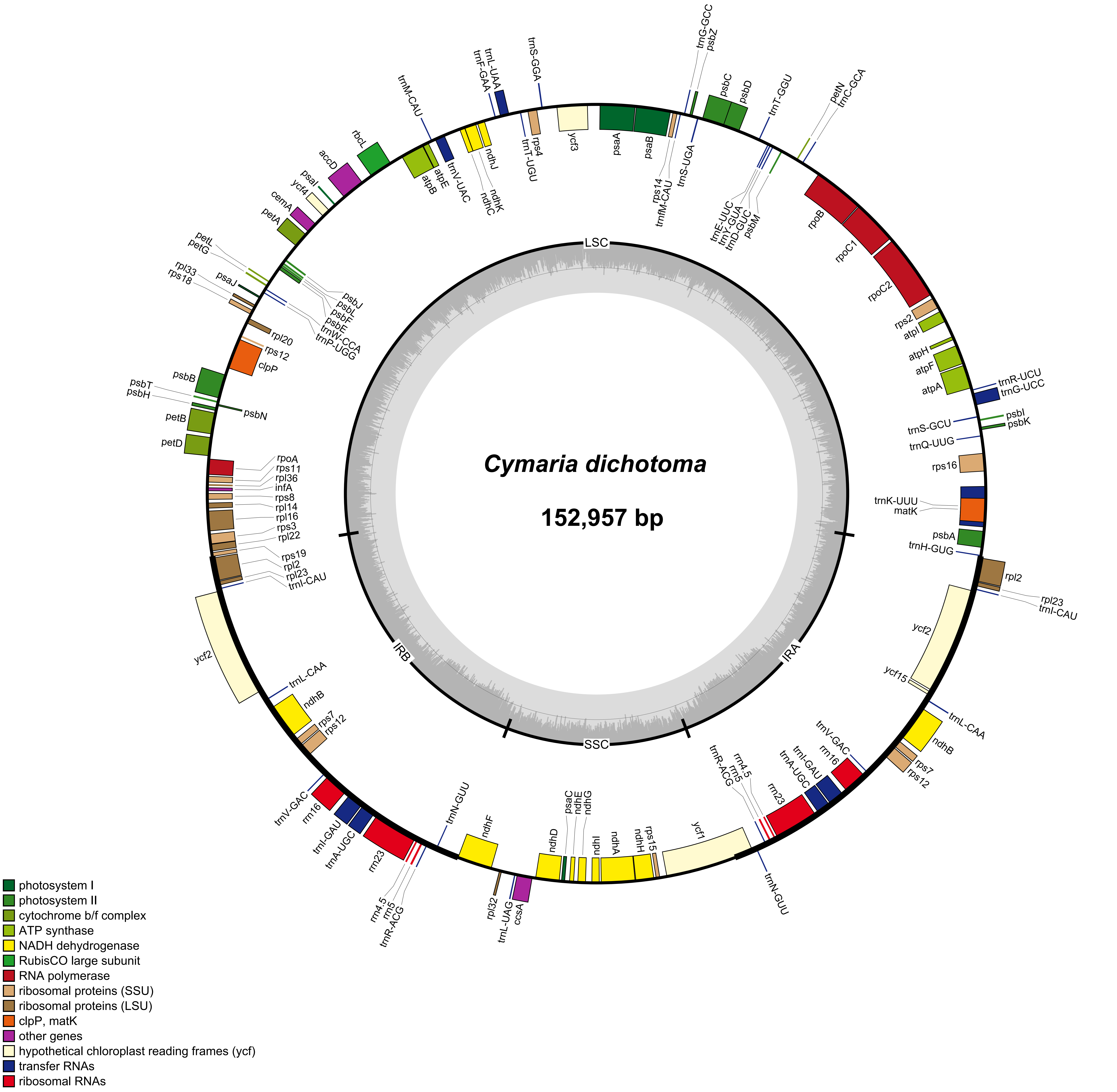
Cymaria dichotoma
152,957 bp
LSC
SSC
IRA
IRB
trnG-GCC
psbZ
psbC
psbD
trnT-GGU
petN
trnC-GCA
trnS-GGA
trnL-UAA
trnF-GAA
trnM-CAU
rps4
trnT-UGU
ycf3
psaA
psaB
rps14
trnfM-CAU
trnS-UGA
trnE-UUC
trnY-GUA
trnD-GUC
psbM
rpoB
rpoC1
rpoC2
rps2
atpI
atpH
atpF
atpA
trnR-UCU
trnG-UCC
psbI
psbK
trnS-GCU
trnQ-UUG
rps16
trnK-UUU
matK
psbA
trnH-GUG
rpl2
rpl23
trnI-CAU
ycf2
ycf15
trnL-CAA
ndhB
rps7
rps12
trnV-GAC
rrn16
trnI-GAU
trnA-UGC
rrn23
rrn4.5
rrn5
trnR-ACG
trnN-GUU
ycf1
rps15
ndhH
ndhA
ndhI
ndhG
ndhE
psaC
ndhD
ccsA
trnL-UAG
rpl32
ndhF
rbcL
atpE
atpB
trnV-UAC
ndhJ
ndhK
ndhC
accD
psaI
ycf4
cemA
petA
petL
petG
psaJ
rpl33
rps18
psbJ
psbL
psbF
psbE
trnW-CCA
trnP-UGG
rpl20
clpP
psbB
psbT
psbH
psbN
petB
petD
rpoA
rps11
rpl36
infA
rps8
rpl14
rpl16
rps3
rpl22
rps19
photosystem I
photosystem II
cytochrome b/f complex
ATP synthase
NADH dehydrogenase
RubisCO large subunit
RNA polymerase
ribosomal proteins (SSU)
ribosomal proteins (LSU)
clpP, matK
other genes
hypothetical chloroplast reading frames (ycf)
transfer RNAs
ribosomal RNAs

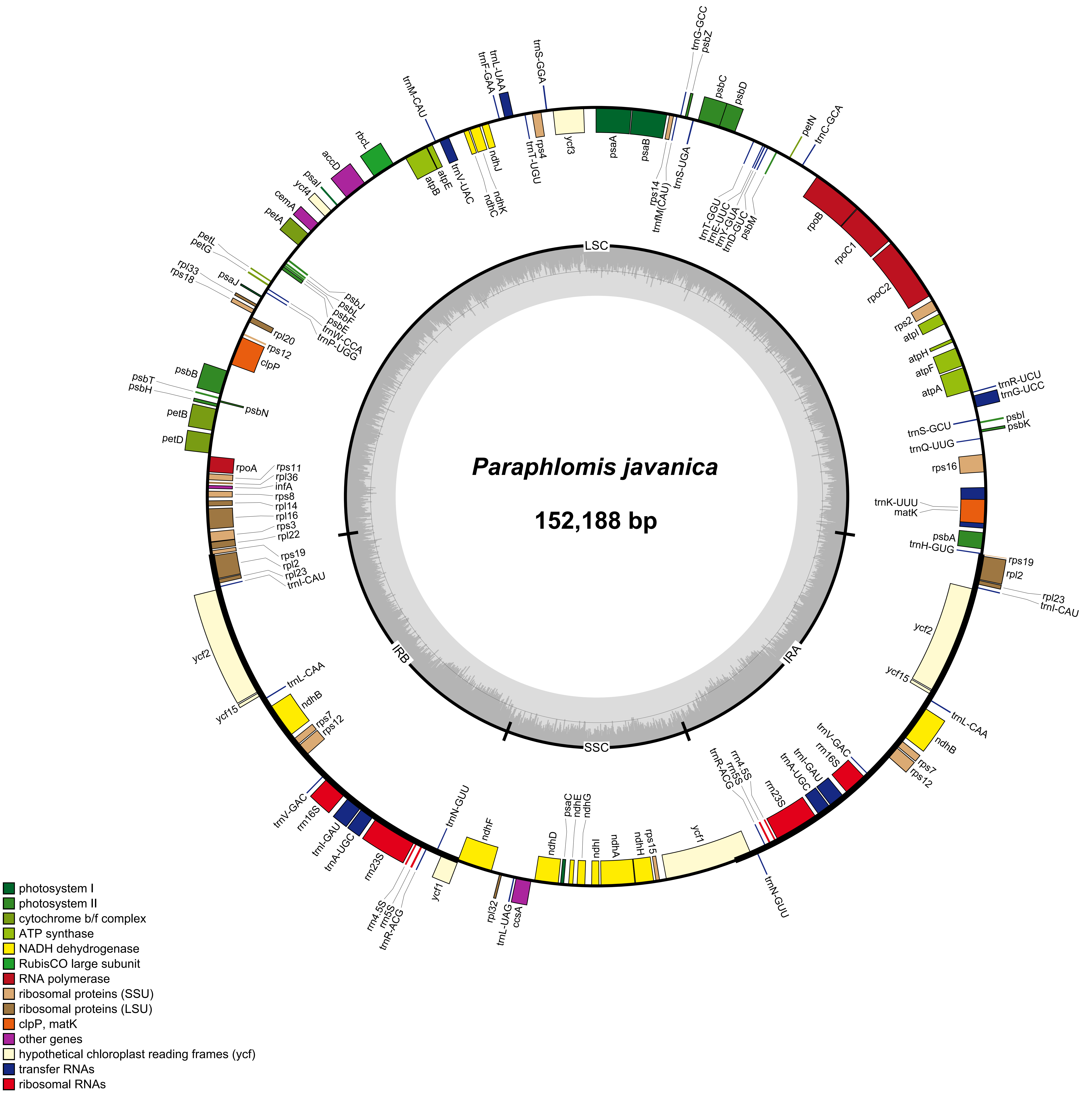
Paraphlomis javanica
152,188 bp
LSC
IRA
SSC
IRB
trnG-GCC
psbZ
psbC
psbD
petN
trnC-GCA
rpoB
rpoC1
rpoC2
rps2
atpI
atpH
atpF
atpA
trnR-UCU
trnG-UCC
psbI
psbK
trnS-GCU
trnQ-UUG
rps16
trnK-UUU
matK
psbA
trnH-GUG
rps19
rpl2
rpl23
trnI-CAU
ycf2
ycf15
trnL-CAA
ndhB
rps7
rps12
trnV-GAC
rrn16S
trnI-GAU
trnA-UGC
rrn23S
rrn4.5S
rrn5S
trnR-ACG
trnN-GUU
ycf1
rps15
ndhH
ndhA
ndhI
ndhG
ndhE
psaC
ndhD
ccsA
trnL-UAG
rpl32
ndhF
rpoA
rps11
rpl36
infA
rps8
rpl14
rpl16
rps3
rpl22
petD
petB
psbN
psbH
psbT
psbB
clpP
rpl20
rpl33
rps18
psaJ
petL
petG
psbJ
psbL
psbF
psbE
trnW-CCA
trnP-UGG
petA
cemA
ycf4
psaI
accD
rbcL
atpB
atpE
trnM-CAU
trnV-UAC
trnF-GAA
trnL-UAA
ndhC
ndhK
ndhJ
trnS-GGA
trnT-UGU
rps4
ycf3
psaA
psaB
rps14
trnfM(CAU)
trnS-UGA
trnT-GGU
trnE-UUC
trnY-GUA
trnD-GUC
psbM
photosystem I
photosystem II
cytochrome b/f complex
ATP synthase
NADH dehydrogenase
RubisCO large subunit
RNA polymerase
ribosomal proteins (SSU)
ribosomal proteins (LSU)
clpP, matK
other genes
hypothetical chloroplast reading frames (ycf)
transfer RNAs
ribosomal RNAs

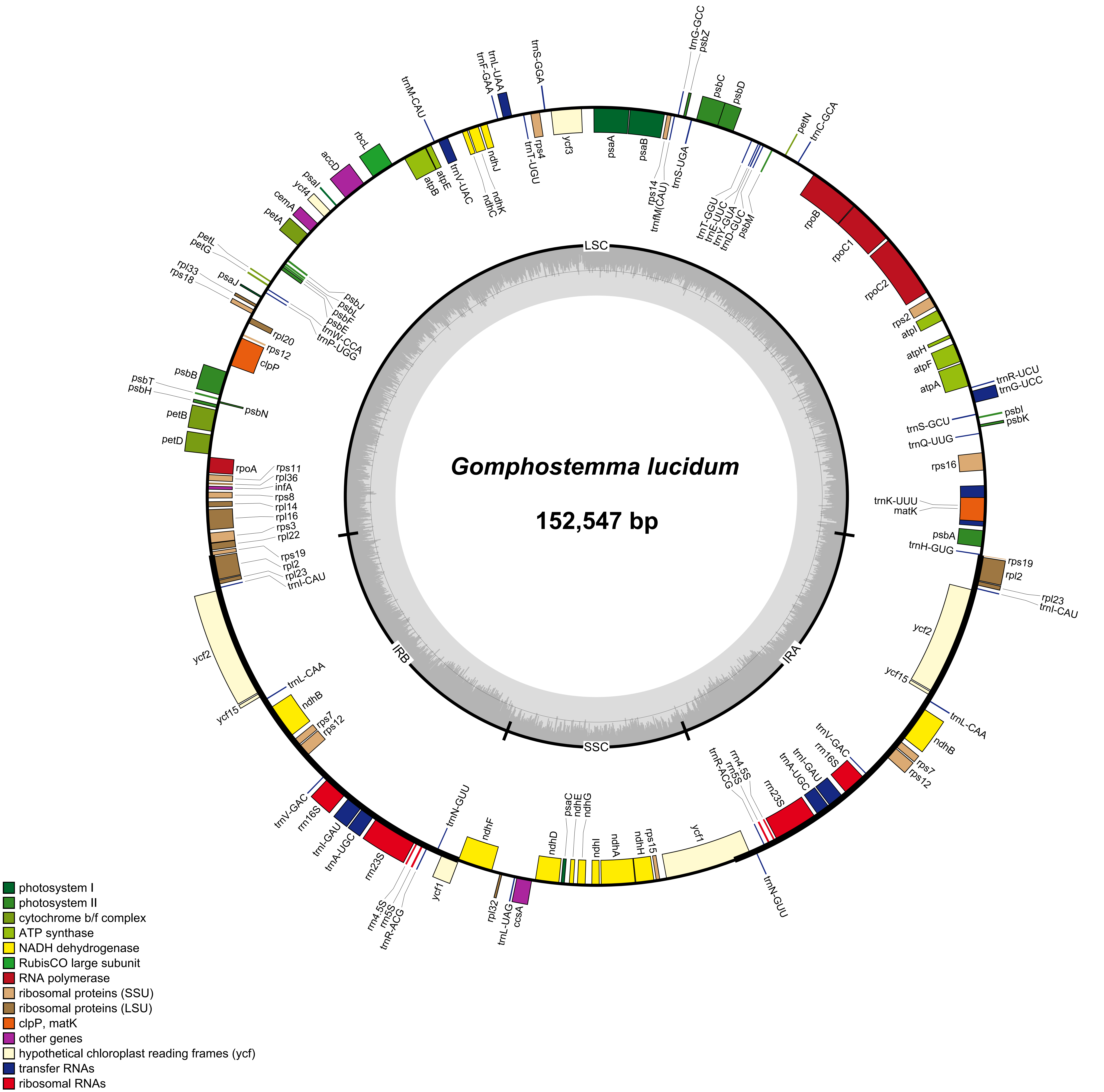
Gomphostemma lucidum
152,547 bp
LSC
SSC
IRA
IRB
photosystem I
photosystem II
cytochrome b/f complex
ATP synthase
NADH dehydrogenase
RubisCO large subunit
RNA polymerase
ribosomal proteins (SSU)
ribosomal proteins (LSU)
clpP, matK
other genes
hypothetical chloroplast reading frames (ycf)
transfer RNAs
ribosomal RNAs

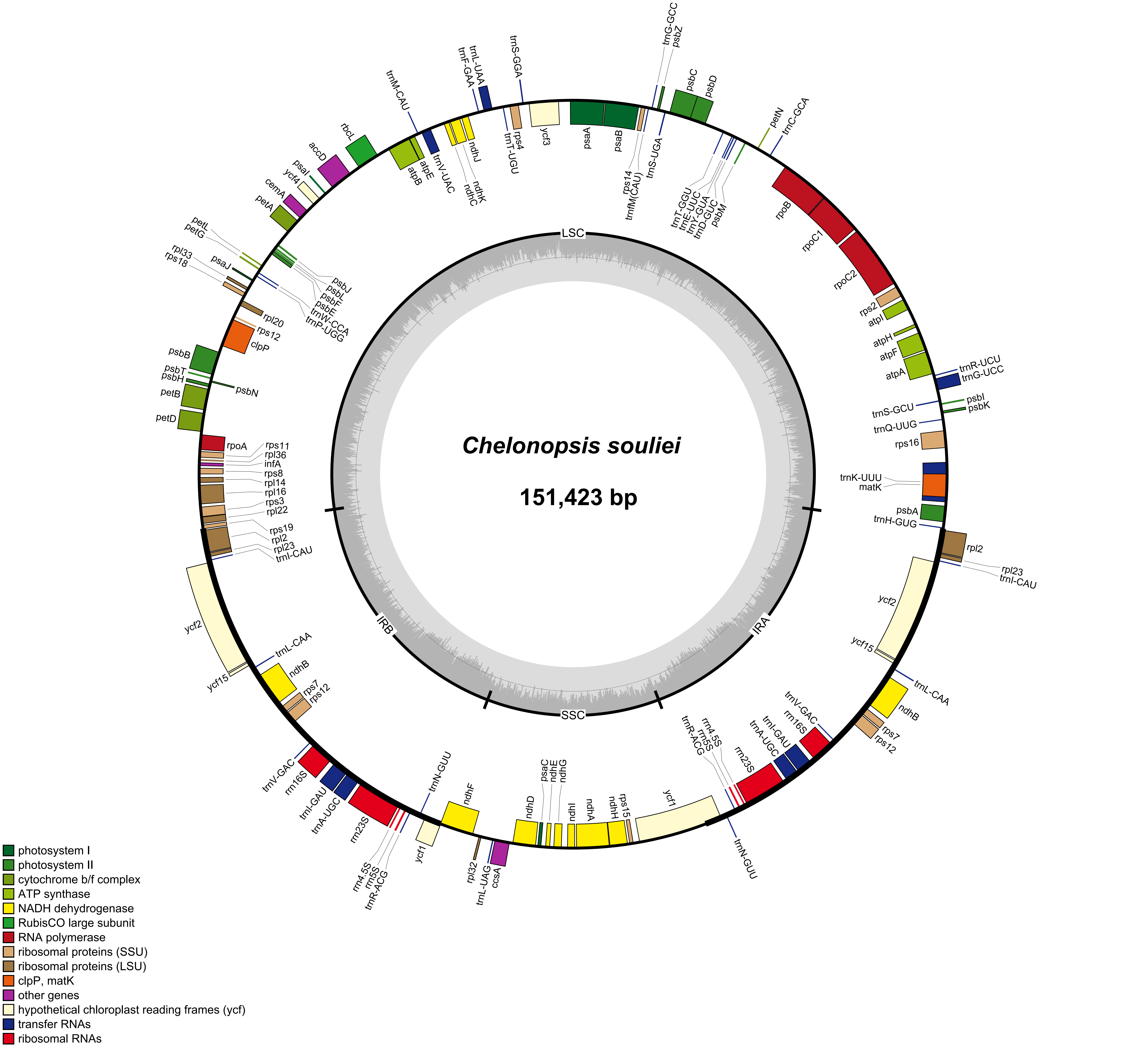
Chelonopsis souliei
151,423 bp
LSC
IRA
SSC
IRB
trnG-GCC
psbZ
psbC
psbD
petN
trnC-GCA
rpoB
rpoC1
rpoC2
rps2
atpI
atpH
atpF
atpA
trnR-UCU
trnG-UCC
psbI
psbK
trnS-GCU
trnQ-UUG
rps16
trnK-UUU
matK
psbA
trnH-GUG
rpl2
rpl23
trnI-CAU
ycf2
ycf15
trnL-CAA
ndhB
rps7
rps12
trnV-GAC
rrn16S
trnI-GAU
trnA-UGC
rrn23S
rrn4.5S
rrn5S
trnR-ACG
trnN-GUU
ycf1
rps15
ndhH
ndhA
ndhI
ndhG
ndhE
psaC
ndhD
ccsA
trnL-UAG
rpl32
ndhF
trnT-GGU
trnE-UUC
trnY-GUA
trnD-GUC
psbM
trnS-UGA
trnfM(CAU)
rps14
psaB
psaA
ycf3
rps4
trnT-UGU
trnS-GGA
trnL-UAA
trnF-GAA
ndhJ
ndhK
ndhC
trnV-UAC
trnM-CAU
atpE
atpB
rbcL
accD
psaI
ycf4
cemA
petA
petL
petG
psbJ
psbL
psbF
psbE
trnW-CCA
trnP-UGG
psaJ
rpl33
rps18
rpl20
clpP
psbB
psbT
psbH
psbN
petB
petD
rpoA
rps11
rpl36
infA
rps8
rpl14
rpl16
rps3
rpl22
rps19
photosystem I
photosystem II
cytochrome b/f complex
ATP synthase
NADH dehydrogenase
RubisCO large subunit
RNA polymerase
ribosomal proteins (SSU)
ribosomal proteins (LSU)
clpP, matK
other genes
hypothetical chloroplast reading frames (ycf)
transfer RNAs
ribosomal RNAs

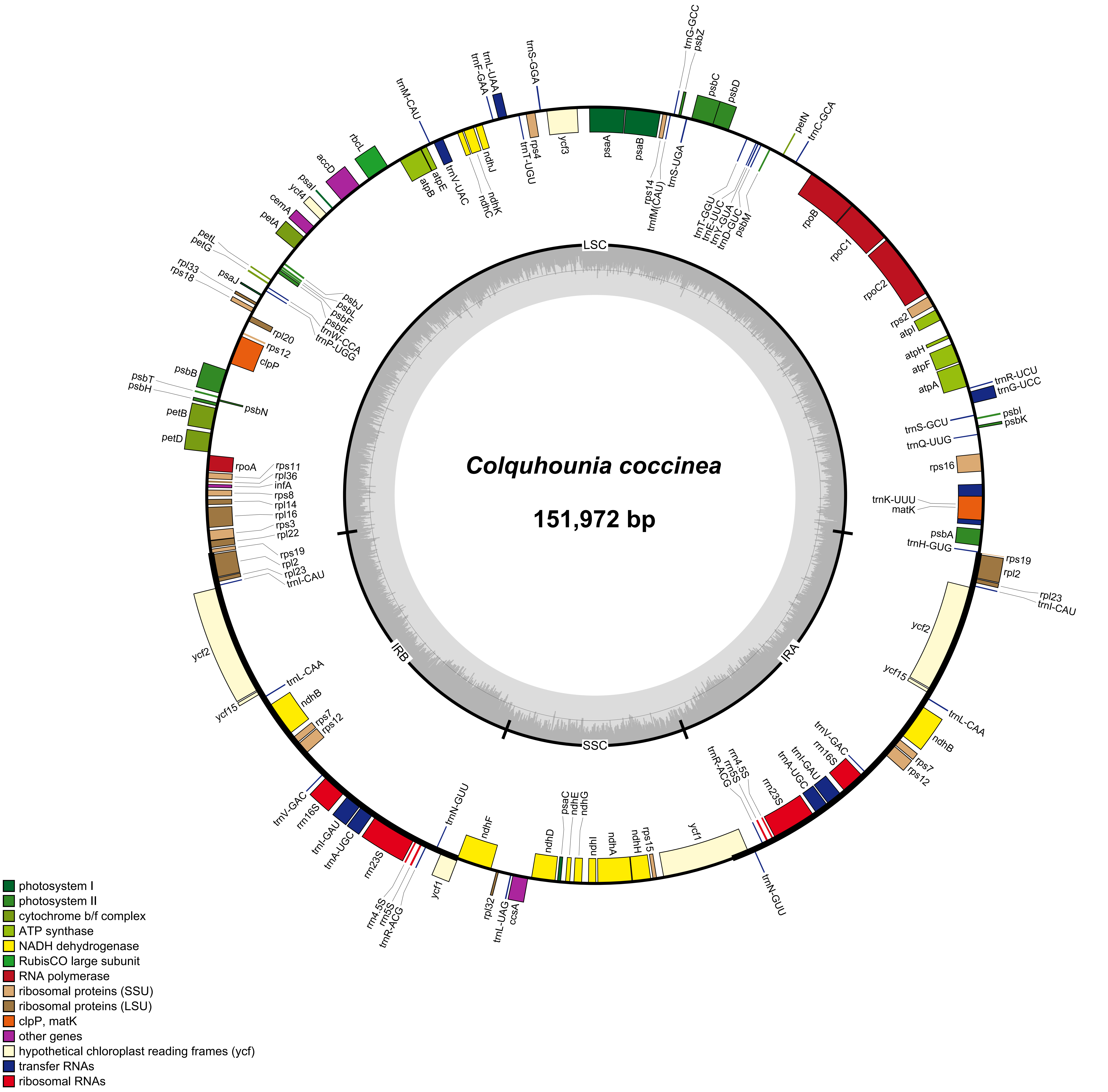

Colquhounia coccinea
151,972 bp
LSC
IRA
SSC
IRB
psaA
psaB
rps14
trnfM(CAU)
trnS-UGA
trnG-GCC
psbZ
psbC
psbD
trnT-GGU
trnE-UUC
trnY-GUA
trnD-GUC
psbM
petN
trnC-GCA
rpoB
rpoC1
rpoC2
rps2
atpI
atpH
atpF
atpA
trnR-UCU
trnG-UCC
psbI
psbK
trnS-GCU
trnQ-UUG
rps16
trnK-UUU
matK
psbA
trnH-GUG
rps19
rpl2
rpl23
trnI-CAU
ycf2
ycf15
trnL-CAA
ndhB
rps7
rps12
trnV-GAC
rrn16S
trnI-GAU
trnA-UGC
rrn23S
rrn4.5S
rrn5S
trnR-ACG
trnN-GUU
ycf1
rps15
ndhH
ndhA
ndhI
ndhG
ndhE
psaC
ndhD
ccsA
trnL-UAG
rpl32
ndhF
rpl22
rps3
rpl16
rpl14
rps8
infA
rpl36
rps11
rpoA
petD
petB
psbN
psbH
psbT
psbB
clpP
rpl20
trnP-UGG
trnW-CCA
psbE
psbF
psbL
psbJ
rps18
rpl33
psaJ
petG
petL
petA
cemA
ycf4
psaI
accD
rbcL
atpB
atpE
trnM-CAU
trnV-UAC
ndhC
ndhK
ndhJ
trnF-GAA
trnL-UAA
trnT-UGU
rps4
trnS-GGA
ycf3
photosystem I
photosystem II
cytochrome b/f complex
ATP synthase
NADH dehydrogenase
RubisCO large subunit
RNA polymerase
ribosomal proteins (SSU)
ribosomal proteins (LSU)
clpP, matK
other genes
hypothetical chloroplast reading frames (ycf)
transfer RNAs
ribosomal RNAs

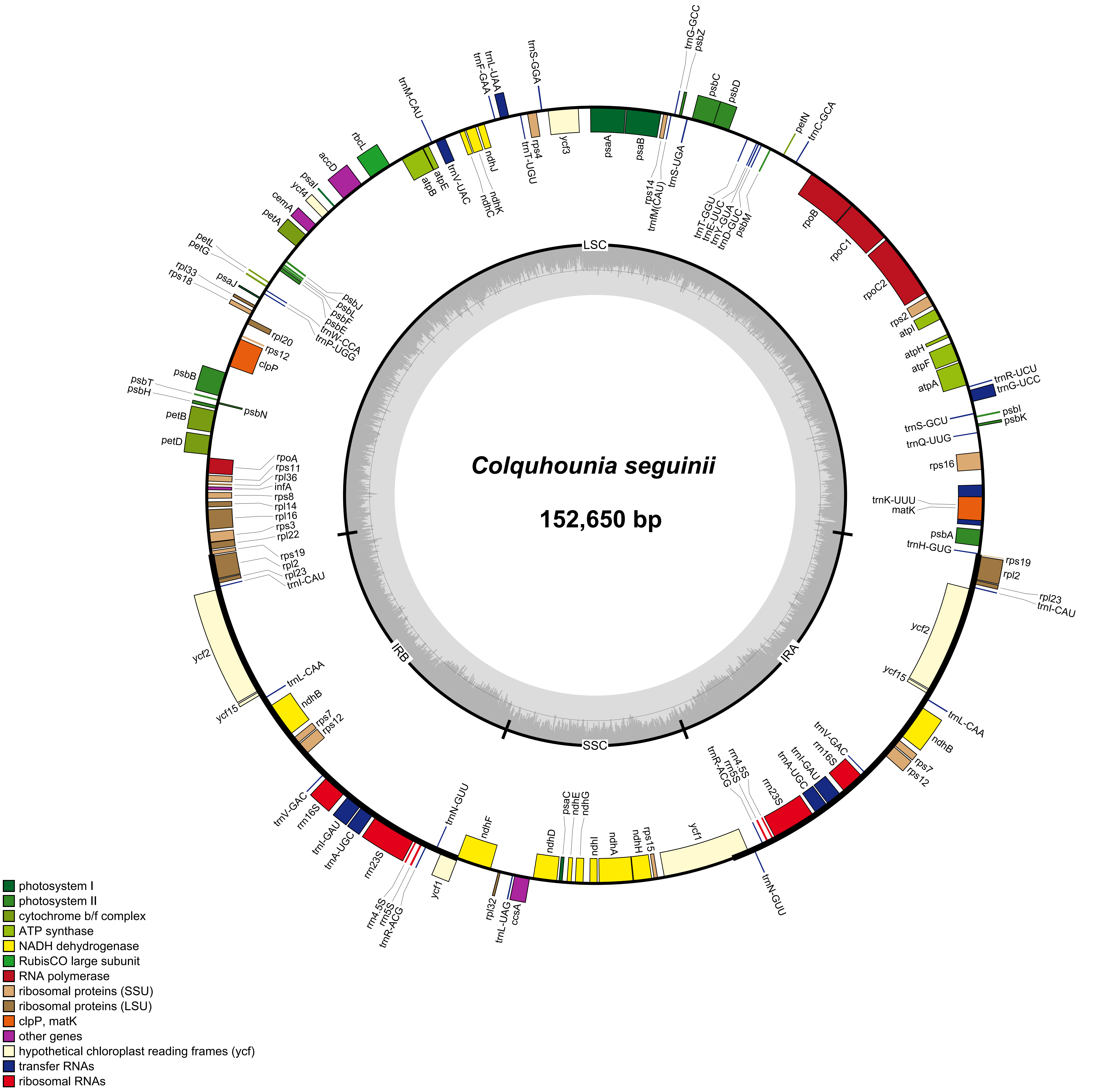

Colquhounia seguinii
152,650 bp
LSC
IRA
SSC
IRB
trnG-GCC
psbZ
psbC
psbD
petN
trnC-GCA
trnS-UGA
trnT-GGU
trnE-UUC
trnY-GUA
trnD-GUC
psbM
rpoB
rpoC1
rpoC2
rps2
atpI
atpH
atpF
atpA
trnR-UCU
trnG-UCC
psbI
psbK
trnS-GCU
trnQ-UUG
rps16
trnK-UUU
matK
psbA
trnH-GUG
rps19
rpl2
rpl23
trnI-CAU
ycf2
ycf15
trnL-CAA
ndhB
rps7
rps12
trnV-GAC
rrn16S
trnI-GAU
trnA-UGC
rrn23S
rrn4.5S
rrn5S
trnR-ACG
trnN-GUU
ycf1
rps15
ndhH
ndhA
ndhI
ndhG
ndhE
psaC
ndhD
ccsA
trnL-UAG
rpl32
ndhF
trnN-GUU
ycf1
trnR-ACG
rrn5S
rrn4.5S
rrn23S
trnA-UGC
trnI-GAU
rrn16S
trnV-GAC
rps12
rps7
ndhB
trnL-CAA
ycf15
ycf2
trnI-CAU
rpl23
rpl2
rps19
rpl22
rps3
rpl16
rpl14
rps8
infA
rpl36
rps11
rpoA
petD
petB
psbH
psbT
psbN
psbB
clpP
rps12
rpl20
rps18
rpl33
psaJ
petG
petL
psbJ
psbL
psbF
psbE
trnW-CCA
trnP-UGG
petA
cemA
ycf4
psaI
accD
rbcL
atpB
atpE
trnM-CAU
trnV-UAC
ndhC
ndhK
ndhJ
trnF-GAA
trnL-UAA
trnT-UGU
rps4
trnS-GGA
ycf3
psaA
psaB
rps14
trnfM(CAU)
photosystem I
photosystem II
cytochrome b/f complex
ATP synthase
NADH dehydrogenase
RubisCO large subunit
RNA polymerase
ribosomal proteins (SSU)
ribosomal proteins (LSU)
clpP, matK
other genes
hypothetical chloroplast reading frames (ycf)
transfer RNAs
ribosomal RNAs

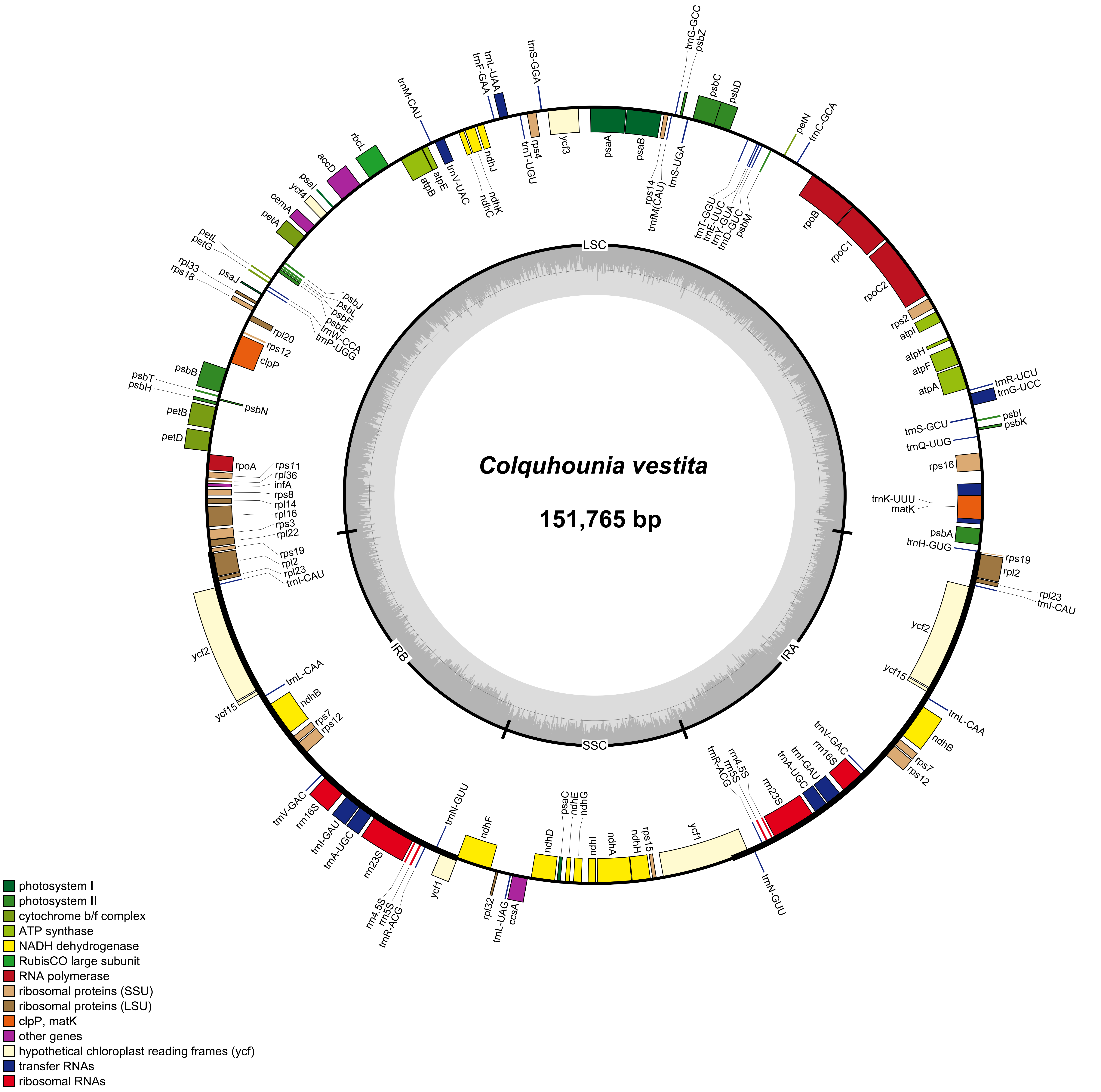

Colquhounia vestita
151,765 bp
LSC
SSC
IRA
IRB
photosystem I
photosystem II
cytochrome b/f complex
ATP synthase
NADH dehydrogenase
RubisCO large subunit
RNA polymerase
ribosomal proteins (SSU)
ribosomal proteins (LSU)
clpP, matK
other genes
hypothetical chloroplast reading frames (ycf)
transfer RNAs
ribosomal RNAs

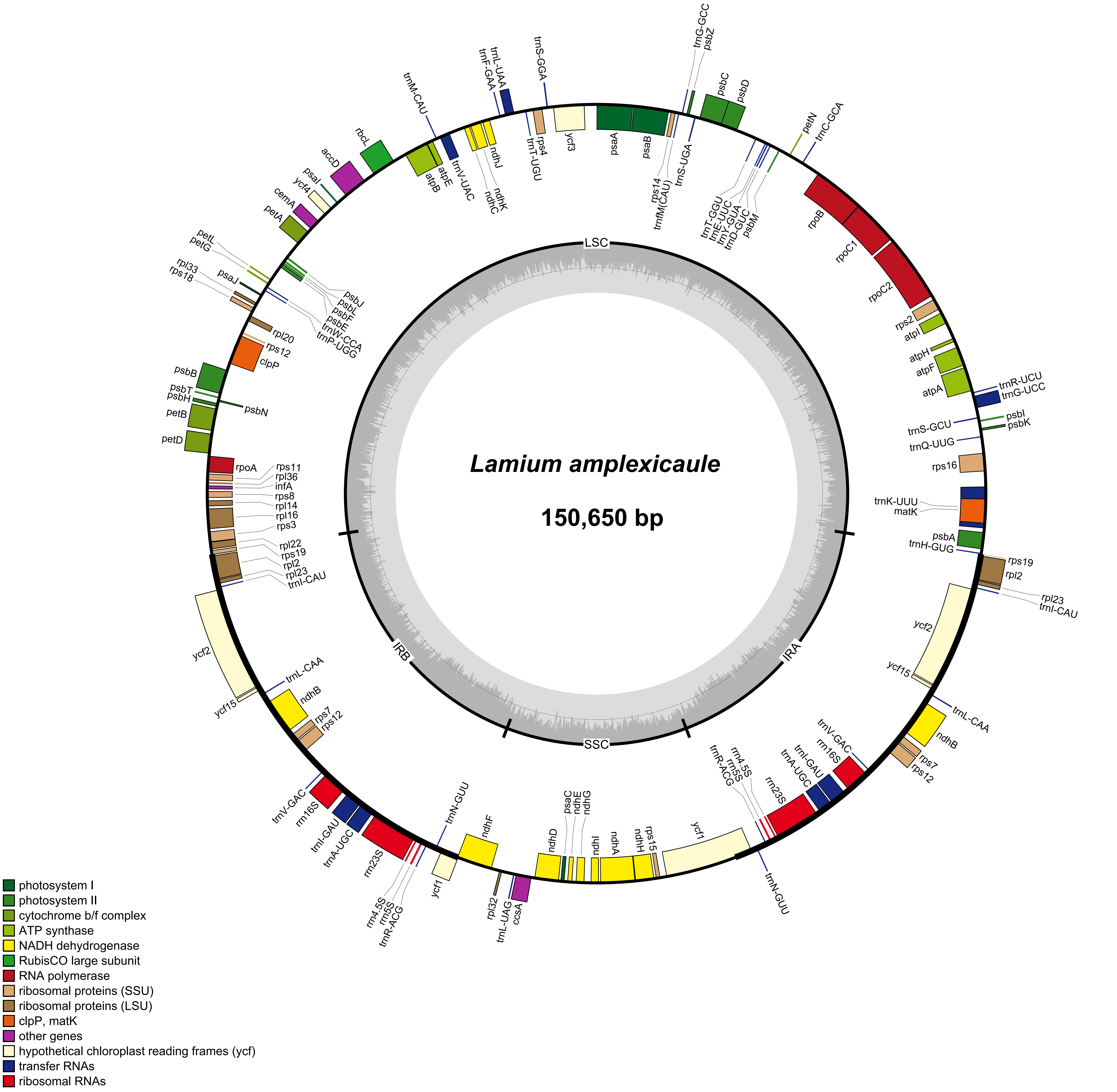

Lamium amplexicaule
150,650 bp
LSC
SSC
IRA
IRB
psaA
psaB
ycf3
rps4
trnT-UGU
trnS-GGA
trnL-UAA
trnF-GAA
ndhJ
ndhK
ndhC
trnV-UAC
trnM-CAU
atpE
atpB
rbcL
accD
psaI
ycf4
cemA
petA
petL
petG
psaJ
rpl33
rps18
psbJ
psbL
psbF
psbE
trnW-CCA
trnP-UGG
rpl20
rps12
clpP
psbB
psbT
psbH
psbN
petB
petD
rpoA
rps11
rpl36
infA
rps8
rpl14
rpl16
rps3
rpl22
rps19
rpl2
rpl23
trnI-CAU
ycf2
ycf15
trnL-CAA
ndhB
rps7
trnV-GAC
rrn16S
trnI-GAU
trnA-UGC
rrn23S
rrn4.5S
rrn5S
trnR-ACG
trnN-GUU
ycf1
ndhF
rpl32
trnL-UAG
ccsA
ndhD
psaC
ndhE
ndhG
ndhI
ndhA
ndhH
rps15
trnG-GCC
psbZ
psbC
psbD
rps14
trnfM(CAU)
trnS-UGA
trnT-GGU
trnE-UUC
trnY-GUA
trnD-GUC
psbM
petN
trnC-GCA
rpoB
rpoC1
rpoC2
rps2
atpI
atpH
atpF
atpA
trnR-UCU
trnG-UCC
psbI
psbK
trnS-GCU
trnQ-UUG
rps16
trnK-UUU
matK
psbA
trnH-GUG
photosystem I
photosystem II
cytochrome b/f complex
ATP synthase
NADH dehydrogenase
RubisCO large subunit
RNA polymerase
ribosomal proteins (SSU)
ribosomal proteins (LSU)
clpP, matK
other genes
hypothetical chloroplast reading frames (ycf)
transfer RNAs
ribosomal RNAs

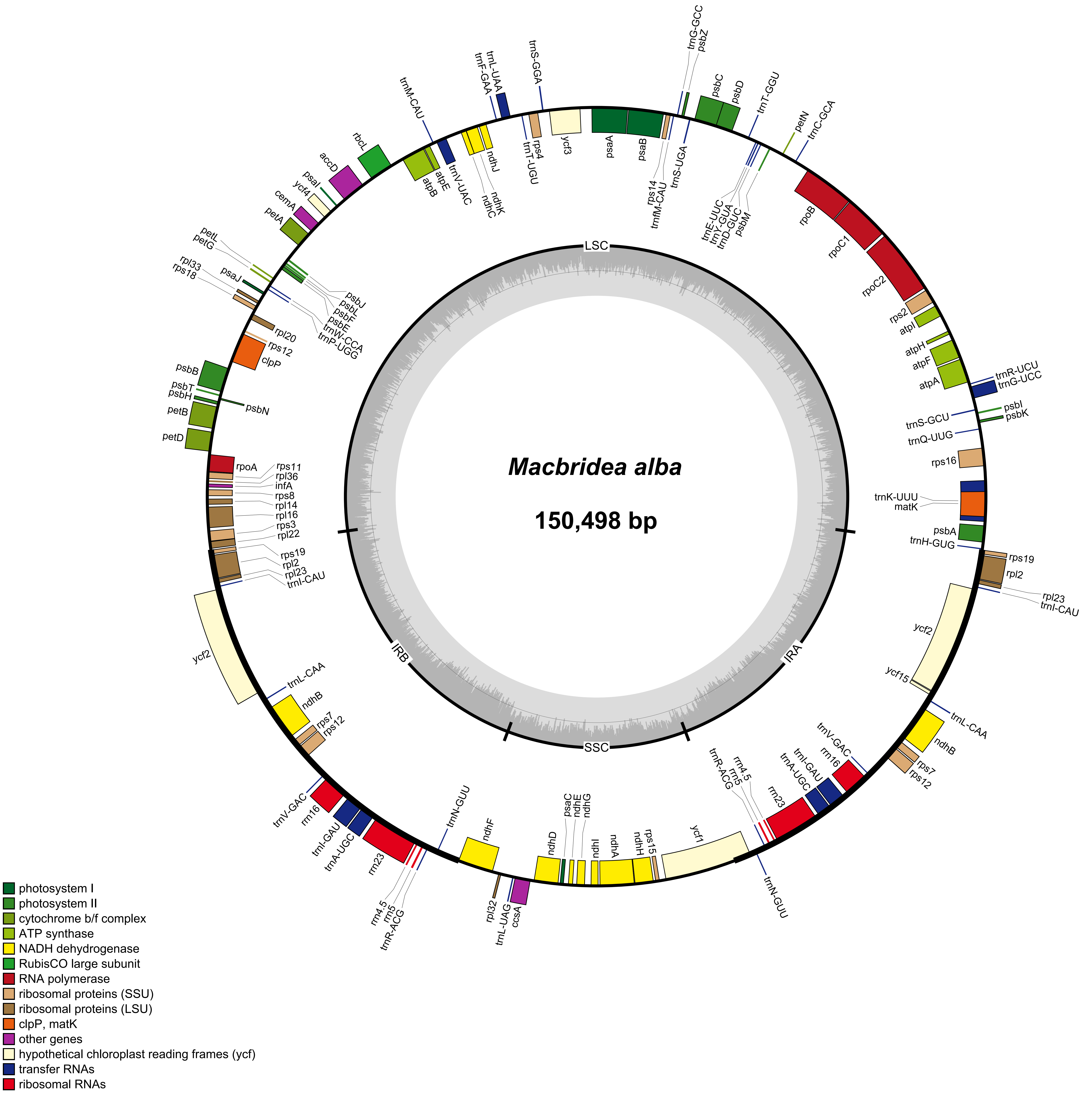

Macbridea alba
150,498 bp
LSC
SSC
IRA
IRB
trnG-GCC
psbZ
psbC
psbD
trnT-GGU
petN
trnC-GCA
trnS-GGA
trnL-UAA
trnF-GAA
trnM-CAU
rps4
ycf3
psaA
psaB
trnS-UGA
rps14
trnfM-CAU
trnT-UGU
ndhJ
ndhK
ndhC
trnV-UAC
atpE
atpB
rbcL
accD
psaI
ycf4
cemA
petA
trnE-UUC
trnY-GUA
trnD-GUC
psbM
rpoB
rpoC1
rpoC2
rps2
atpI
atpH
atpF
atpA
trnR-UCU
trnG-UCC
psbI
psbK
trnS-GCU
trnQ-UUG
rps16
trnK-UUU
matK
psbA
trnH-GUG
rps19
rpl2
rpl23
trnI-CAU
ycf2
ycf15
trnL-CAA
ndhB
rps7
rps12
trnV-GAC
rrn16
trnI-GAU
trnA-UGC
rrn23
rrn4.5
rrn5
trnR-ACG
trnN-GUU
ycf1
rps15
ndhH
ndhA
ndhI
ndhG
ndhE
psaC
ndhD
ccsA
trnL-UAG
rpl32
ndhF
petL
petG
rpl33
rps18
psaJ
psbJ
psbL
psbF
psbE
trnW-CCA
trnP-UGG
rpl20
clpP
psbB
psbT
psbH
psbN
petB
petD
rpoA
rps11
rpl36
infA
rps8
rpl14
rpl16
rps3
rpl22
photosystem I
photosystem II
cytochrome b/f complex
ATP synthase
NADH dehydrogenase
RubisCO large subunit
RNA polymerase
ribosomal proteins (SSU)
ribosomal proteins (LSU)
clpP, matK
other genes
hypothetical chloroplast reading frames (ycf)
transfer RNAs
ribosomal RNAs

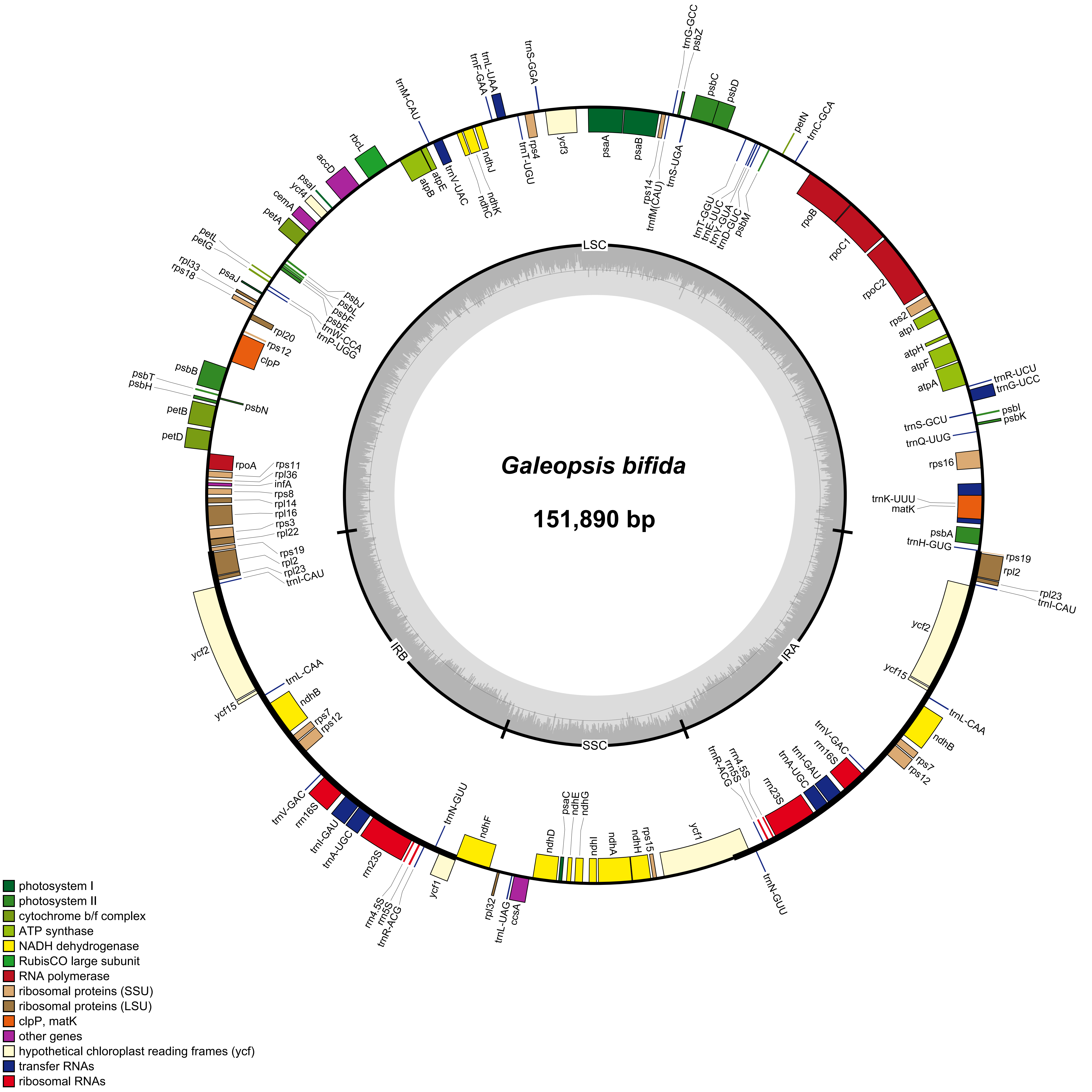
Galeopsis bifida
151,890 bp
LSC
IRA
SSC
IRB
trnG-GCC
psbZ
psbC
psbD
petN
trnC-GCA
rpoB
rpoC1
rpoC2
rps2
atpI
atpH
atpF
atpA
trnR-UCU
trnG-UCC
psbI
psbK
trnS-GCU
trnQ-UUG
rps16
trnK-UUU
matK
psbA
trnH-GUG
rps19
rpl2
rpl23
trnI-CAU
ycf2
ycf15
trnL-CAA
ndhB
rps7
rps12
trnV-GAC
rrn16S
trnI-GAU
trnA-UGC
rrn23S
rrn4.5S
rrn5S
trnR-ACG
trnN-GUU
ycf1
rps15
ndhH
ndhA
ndhI
ndhG
ndhE
psaC
ndhD
ccsA
trnL-UAG
rpl32
ndhF
rpoA
rps11
rpl36
infA
rps8
rpl14
rpl16
rps3
rpl22
petD
petB
psbN
psbH
psbT
psbB
clpP
rpl20
rpl33
rps18
psaJ
petL
petG
psbJ
psbL
psbF
psbE
trnW-CCA
trnP-UGG
petA
cemA
ycf4
psaI
accD
rbcL
atpB
atpE
trnV-UAC
trnM-CAU
ndhC
ndhK
ndhJ
trnF-GAA
trnL-UAA
trnT-UGU
rps4
trnS-GGA
ycf3
psaA
psaB
rps14
trnfM-CAU
trnS-UGA
trnT-GGU
trnE-UUC
trnY-GUA
trnD-GUC
psbM
photosystem I
photosystem II
cytochrome b/f complex
ATP synthase
NADH dehydrogenase
RubisCO large subunit
RNA polymerase
ribosomal proteins (SSU)
ribosomal proteins (LSU)
clpP, matK
other genes
hypothetical chloroplast reading frames (ycf)
transfer RNAs
ribosomal RNAs

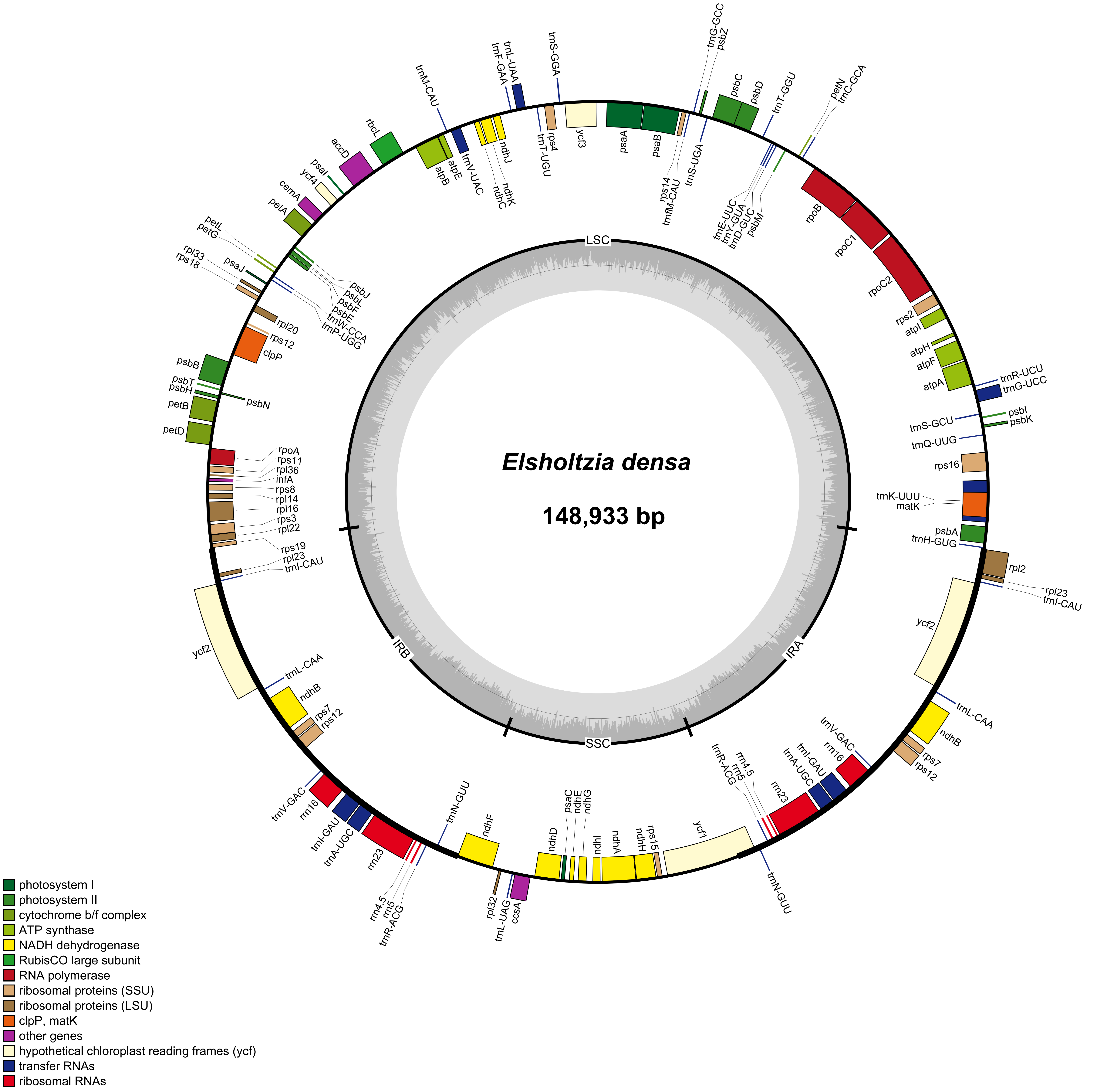

Elsholtzia densa
148,933 bp
LSC
IRA
IRB
SSC
photosystem I
photosystem II
cytochrome b/f complex
ATP synthase
NADH dehydrogenase
RubisCO large subunit
RNA polymerase
ribosomal proteins (SSU)
ribosomal proteins (LSU)
clpP, matK
other genes
hypothetical chloroplast reading frames (ycf)
transfer RNAs
ribosomal RNAs

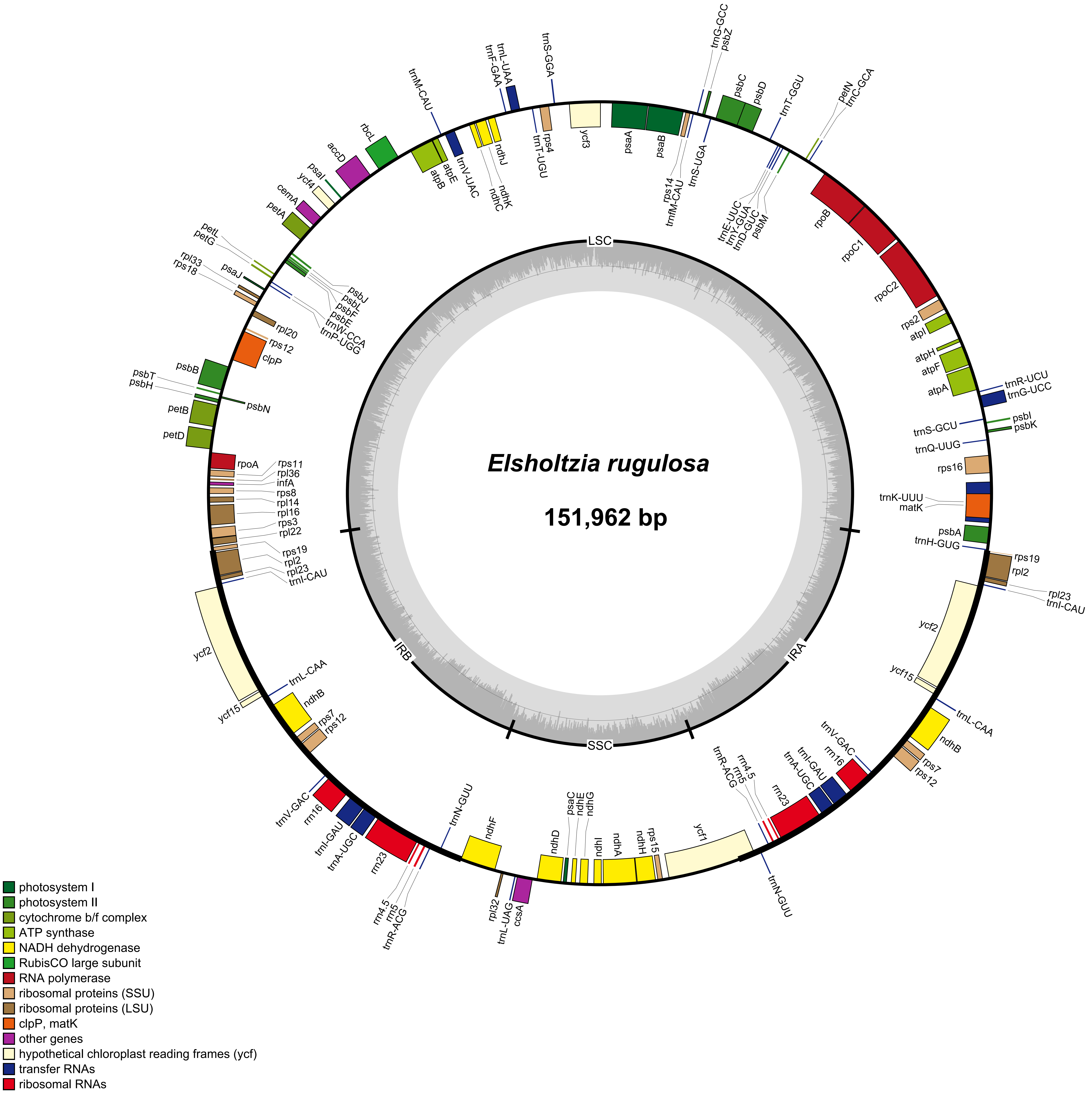
Elsholtzia rugulosa
151,962 bp
LSC
IRA
SSC
IRB
trnG-GCC
psbZ
psbC
psbD
trnT-GGU
petN
trnC-GCA
trnS-GGA
trnL-UAA
trnF-GAA
trnM-CAU
rbcL
accD
psaI
ycf4
cemA
petA
petL
petG
rpl33
rps18
psaJ
psbJ
psbL
psbF
psbE
trnW-CCA
trnP-UGG
rpl20
rps12
clpP
psbB
psbT
psbH
psbN
petB
petD
rpoA
rps11
rpl36
infA
rps8
rpl14
rpl16
rps3
rpl22
rps19
rpl2
rpl23
trnI-CAU
ycf2
ycf15
trnL-CAA
ndhB
rps7
trnV-GAC
rrn16
trnI-GAU
trnA-UGC
rrn23
rrn4.5
rrn5
trnR-ACG
trnN-GUU
ndhF
rpl32
trnL-UAG
ccsA
ndhD
psaC
ndhE
ndhG
ndhI
ndhA
ndhH
rps15
ycf1
atpE
atpB
trnV-UAC
ndhJ
ndhK
ndhC
rps4
trnT-UGU
ycf3
psaA
psaB
rps14
trnfM-CAU
trnS-UGA
trnE-UUC
trnY-GUA
trnD-GUC
psbM
rpoB
rpoC1
rpoC2
rps2
atpI
atpH
atpF
atpA
trnR-UCU
trnG-UCC
psbI
psbK
trnS-GCU
trnQ-UUG
rps16
trnK-UUU
matK
psbA
trnH-GUG
photosystem I
photosystem II
cytochrome b/f complex
ATP synthase
NADH dehydrogenase
RubisCO large subunit
RNA polymerase
ribosomal proteins (SSU)
ribosomal proteins (LSU)
clpP, matK
other genes
hypothetical chloroplast reading frames (ycf)
transfer RNAs
ribosomal RNAs

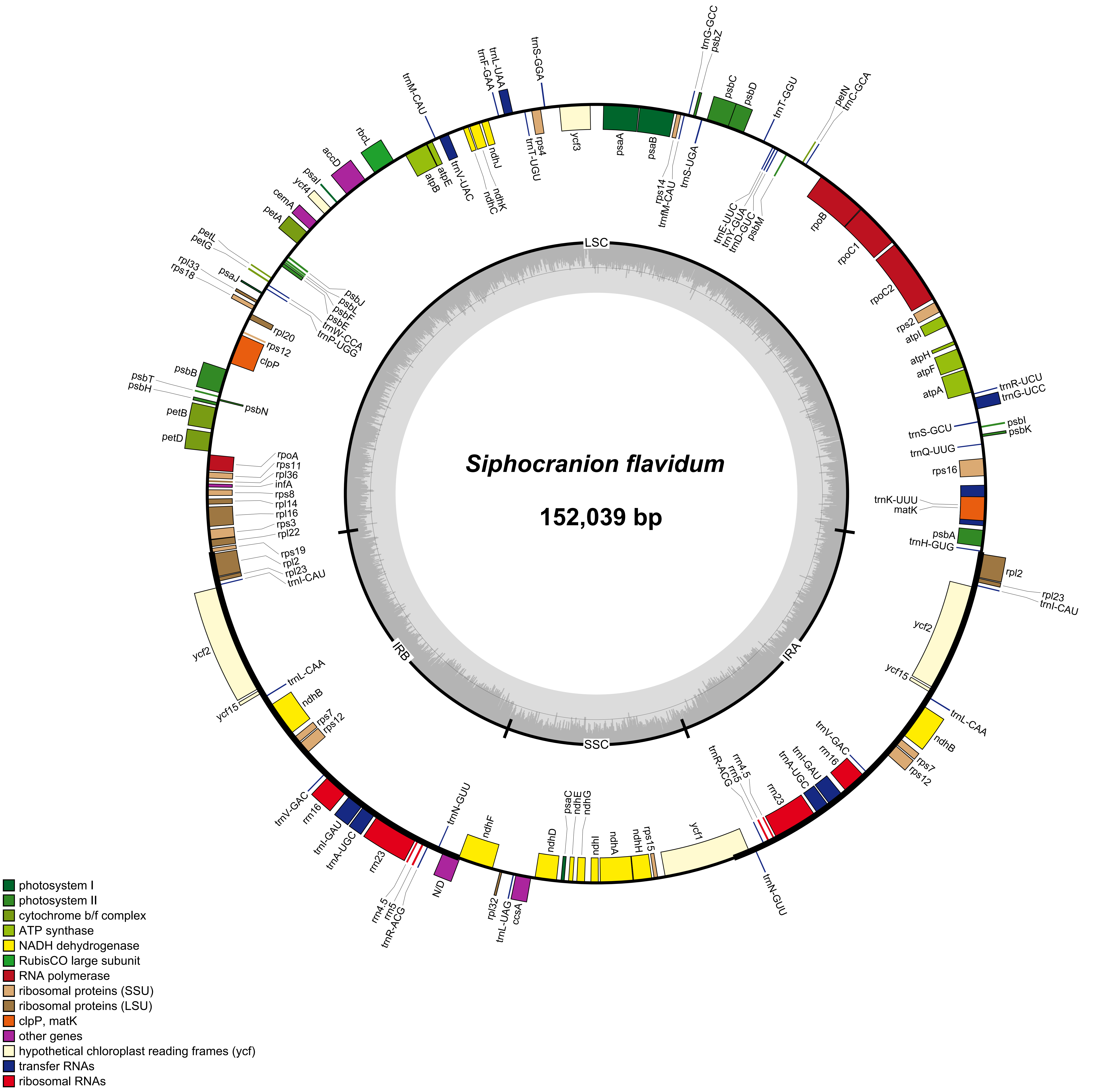

Siphocranion flavidum
152,039 bp
LSC
IRA
SSC
IRB
psbA
trnH-GUG
matK
trnK-UUU
rps16
trnQ-UUG
trnS-GCU
psbK
psbI
trnG-UCC
trnR-UCU
atpA
atpF
atpH
atpI
rps2
rpoC2
rpoC1
rpoB
trnC-GCA
petN
psbM
trnD-GUC
trnY-GUA
trnE-UUC
trnT-GGU
psbD
psbC
trnS-UGA
psbZ
trnG-GCC
trnfM-CAU
rps14
psaB
psaA
ycf3
trnS-GGA
rps4
trnT-UGU
trnL-UAA
trnF-GAA
ndhJ
ndhK
ndhC
trnV-UAC
trnM-CAU
atpE
atpB
rbcL
accD
psaI
ycf4
cemA
petA
psbJ
psbL
psbF
psbE
petL
petG
trnW-CCA
trnP-UGG
psaJ
rpl33
rps18
rpl20
rps12
clpP
psbB
psbT
psbH
psbN
petB
petD
rpoA
rps11
rpl36
infA
rps8
rpl14
rpl16
rps3
rpl22
rps19
rpl2
rpl23
trnI-CAU
ycf2
ycf15
trnL-CAA
ndhB
rps7
rps12
trnV-GAC
rrn16
trnI-GAU
trnA-UGC
rrn23
rrn4.5
rrn5
trnR-ACG
trnN-GUU
ndhF
rpl32
trnL-UAG
ccsA
ndhD
psaC
ndhE
ndhG
ndhI
ndhA
ndhH
rps15
ycf1
photosystem I
photosystem II
cytochrome b/f complex
ATP synthase
NADH dehydrogenase
RubisCO large subunit
RNA polymerase
ribosomal proteins (SSU)
ribosomal proteins (LSU)
clpP, matK
other genes
hypothetical chloroplast reading frames (ycf)
transfer RNAs
ribosomal RNAs

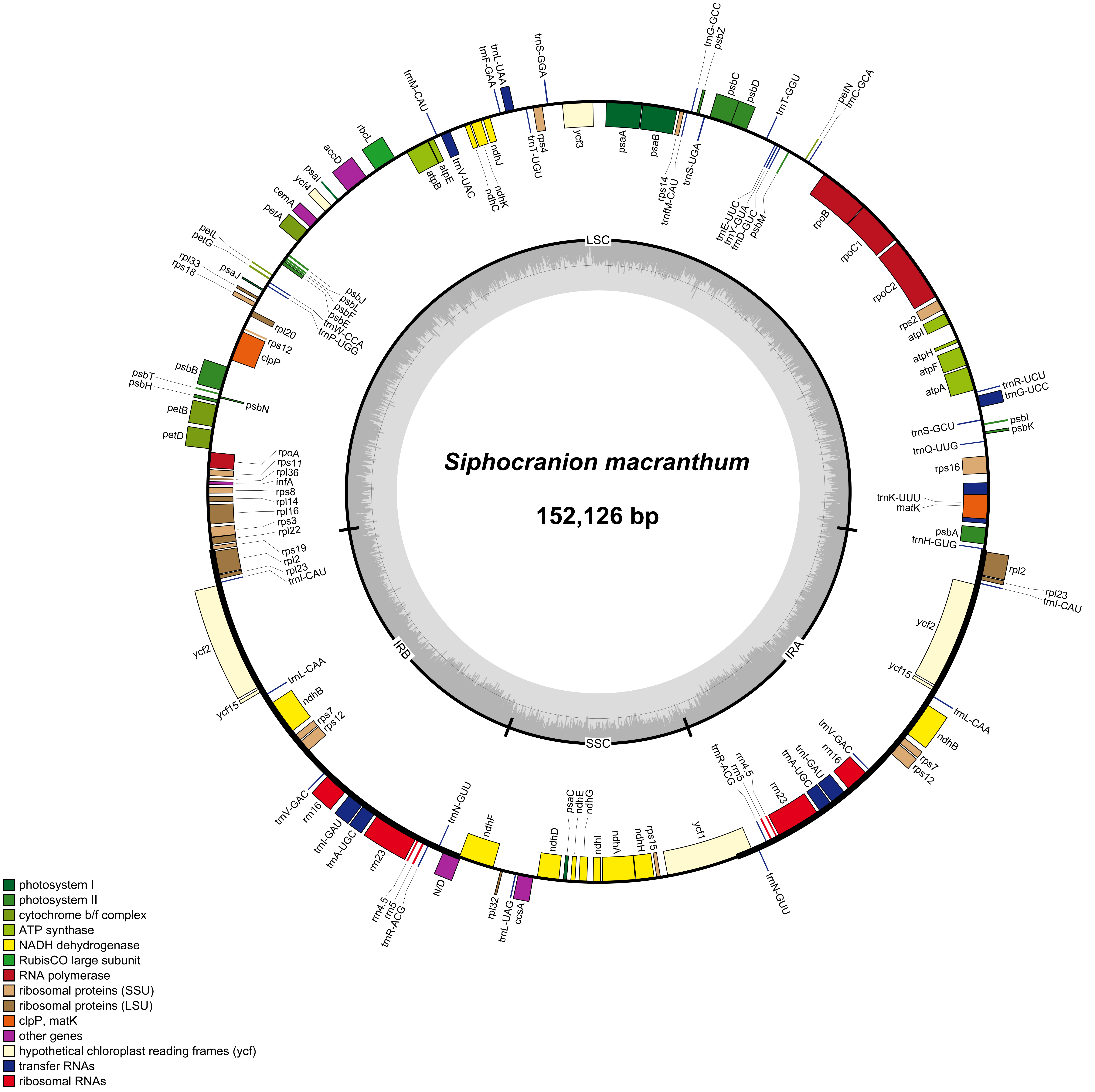
Siphocranion macranthum
152,126 bp
LSC
IRA
SSC
IRB
trnG-GCC
psbZ
psbC
psbD
trnT-GGU
petN
trnC-GCA
rpoB
rpoC1
rpoC2
rps2
atpI
atpH
atpF
atpA
trnR-UCU
trnG-UCC
psbI
psbK
trnS-GCU
trnQ-UUG
rps16
trnK-UUU
matK
psbA
trnH-GUG
rpl2
rpl23
trnI-CAU
ycf2
ycf15
trnL-CAA
ndhB
rps7
rps12
trnV-GAC
rrn16
trnI-GAU
trnA-UGC
rrn23
rrn4.5
rrn5
trnR-ACG
trnN-GUU
ycf1
rps15
ndhH
ndhA
ndhI
ndhG
ndhE
psaC
ndhD
ccsA
trnL-UAG
rpl32
ndhF
NID
trnI-CAU
rpl23
rpl2
rps19
rpl22
rps3
rpl16
rpl14
rps8
infA
rpl36
rps11
rpoA
petD
petB
psbN
psbH
psbT
psbB
clpP
rps12
rpl20
rps18
rpl33
psaJ
petG
petL
trnP-UGG
trnW-CCA
psbE
psbF
psbL
psbJ
petA
cemA
ycf4
psaI
accD
rbcL
atpB
atpE
trnV-UAC
trnM-CAU
ndhC
ndhK
ndhJ
trnF-GAA
trnL-UAA
trnT-UGU
rps4
trnS-GGA
ycf3
psaA
psaB
rps14
trnfM-CAU
trnS-UGA
trnE-UUC
trnY-GUA
trnD-GUC
psbM
photosystem I
photosystem II
cytochrome b/f complex
ATP synthase
NADH dehydrogenase
RubisCO large subunit
RNA polymerase
ribosomal proteins (SSU)
ribosomal proteins (LSU)
clpP, matK
other genes
hypothetical chloroplast reading frames (ycf)
transfer RNAs
ribosomal RNAs

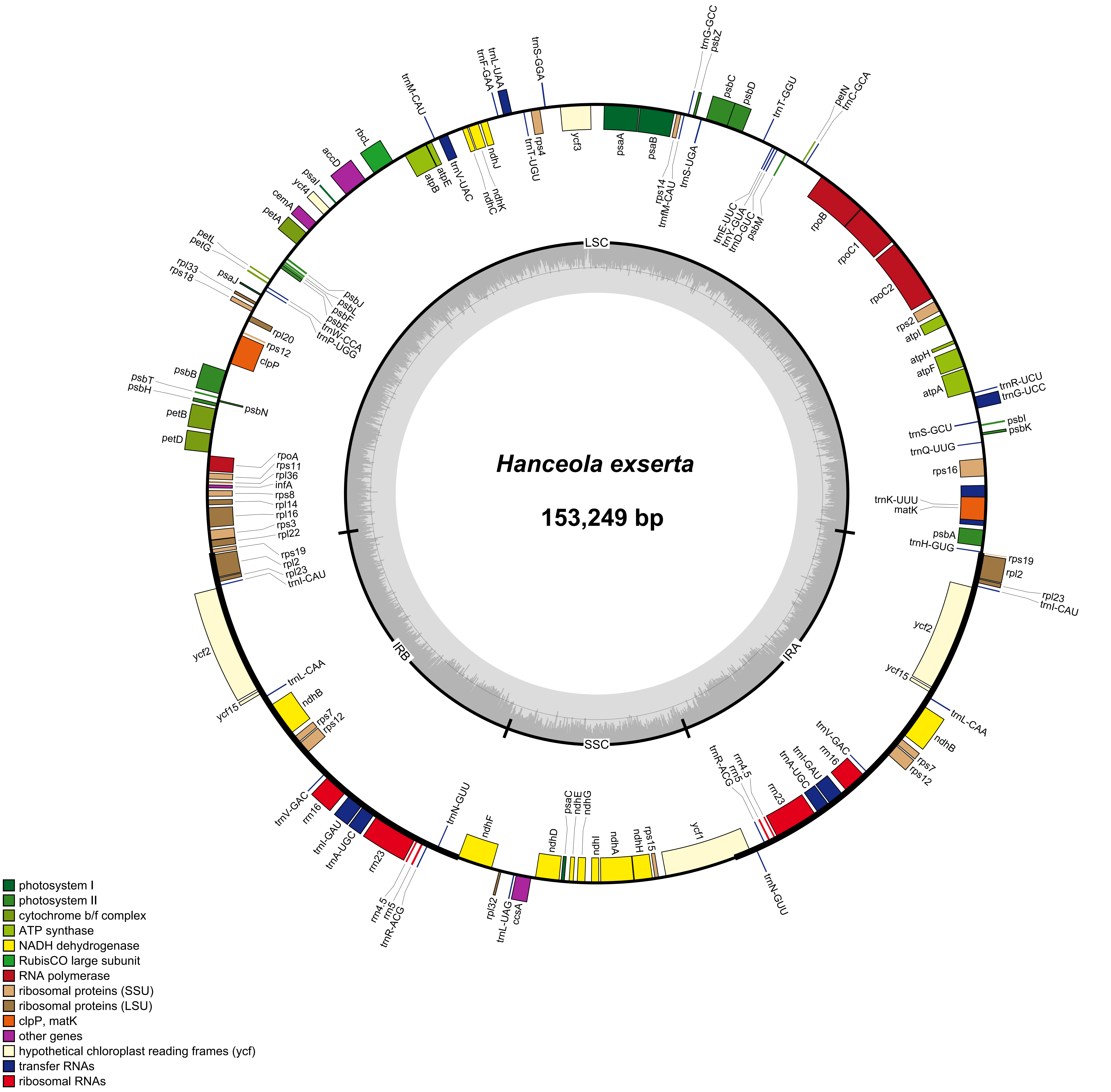

Hanceola exserta
153,249 bp
LSC
IRA
SSC
IRB
trnG-GCC
psbZ
psbC
psbD
trnT-GGU
petN
trnC-GCA
trnS-GGA
trnL-UAA
trnF-GAA
trnM-CAU
rbcL
accD
psaI
ycf4
cemA
petA
petL
petG
rpl33
rps18
psaJ
psbJ
psbL
psbF
psbE
trnW-CCA
trnP-UGG
rpl20
rps12
clpP
psbB
psbT
psbH
psbN
petB
petD
rpoA
rps11
rpl36
infA
rps8
rpl14
rpl16
rps3
rpl22
rps19
rpl2
rpl23
trnI-CAU
ycf2
trnL-CAA
ndhB
rps7
rps12
ycf15
trnV-GAC
rrn16
trnI-GAU
trnA-UGC
rrn23
rrn4.5
rrn5
trnR-ACG
trnN-GUU
ndhF
rpl32
trnL-UAG
ccsA
ndhD
psaC
ndhE
ndhG
ndhI
ndhA
ndhH
rps15
ycf1
atpB
atpE
trnV-UAC
ndhC
ndhK
ndhJ
trnT-UGU
rps4
ycf3
psaA
psaB
rps14
trnfM-CAU
trnS-UGA
trnE-UUC
trnY-GUA
trnD-GUC
psbM
rpoB
rpoC1
rpoC2
rps2
atpI
atpH
atpF
atpA
trnR-UCU
trnG-UCC
psbI
psbK
trnS-GCU
trnQ-UUG
rps16
trnK-UUU
matK
psbA
trnH-GUG
photosystem I
photosystem II
cytochrome b/f complex
ATP synthase
NADH dehydrogenase
RubisCO large subunit
RNA polymerase
ribosomal proteins (SSU)
ribosomal proteins (LSU)
clpP, matK
other genes
hypothetical chloroplast reading frames (ycf)
transfer RNAs
ribosomal RNAs

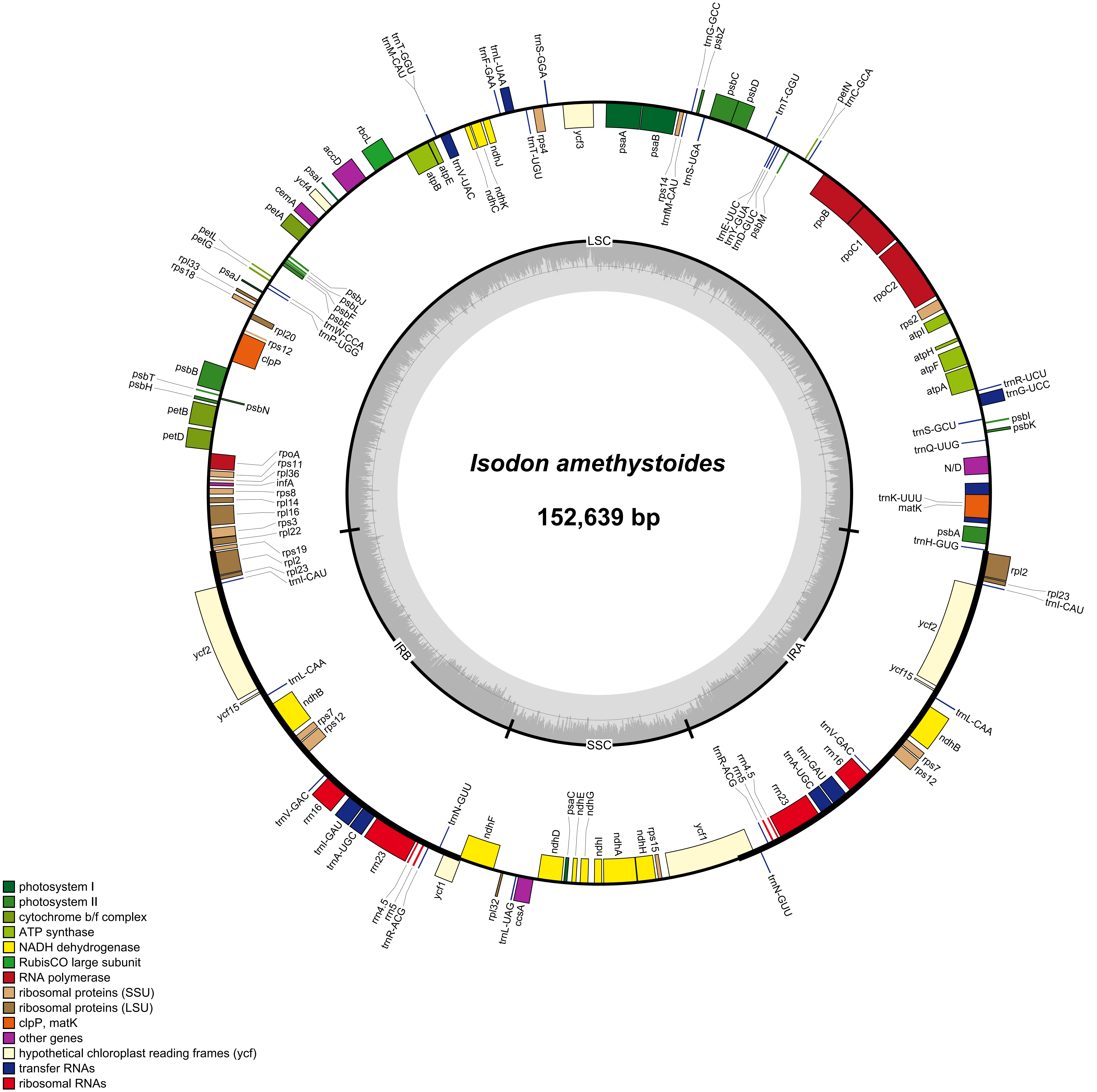

Isodon amethystoides
152,639 bp
LSC
IRA
SSC
IRB
trnT-GGU
trnM-CAU
trnL-UAA
trnF-GAA
trnS-GGA
trnG-GCC
psbZ
psbC
psbD
trnT-GGU
petN
trnC-GCA
rbcL
accD
psaI
ycf4
cemA
petA
atpE
atpB
trnV-UAC
ndhJ
ndhK
ndhC
rps4
trnT-UGU
ycf3
psaA
psaB
rps14
trnfM-CAU
trnS-UGA
trnE-UUC
trnY-GUA
trnD-GUC
psbM
rpoB
rpoC1
rpoC2
rps2
atpI
atpH
atpF
atpA
trnR-UCU
trnG-UCC
psbI
psbK
trnS-GCU
trnQ-UUG
N/D
trnK-UUU
matK
psbA
trnH-GUG
rpl2
rpl23
trnI-CAU
ycf2
ycf15
trnL-CAA
ndhB
rps7
rps12
trnV-GAC
rrn16
trnI-GAU
trnA-UGC
rrn23
rrn4.5
rrn5
trnR-ACG
trnN-GUU
ycf1
rps15
ndhH
ndhA
ndhI
ndhG
ndhE
psaC
ndhD
ccsA
trnL-UAG
rpl32
ndhF
petL
petG
psaJ
rpl33
rps18
psbJ
psbL
psbF
psbE
trnW-CCA
trnP-UGG
rpl20
rps12
clpP
psbB
psbT
psbH
psbN
petB
petD
rpoA
rps11
rpl36
infA
rps8
rpl14
rpl16
rps3
rpl22
rps19
photosystem I
photosystem II
cytochrome b/f complex
ATP synthase
NADH dehydrogenase
RubisCO large subunit
RNA polymerase
ribosomal proteins (SSU)
ribosomal proteins (LSU)
clpP, matK
other genes
hypothetical chloroplast reading frames (ycf)
transfer RNAs
ribosomal RNAs

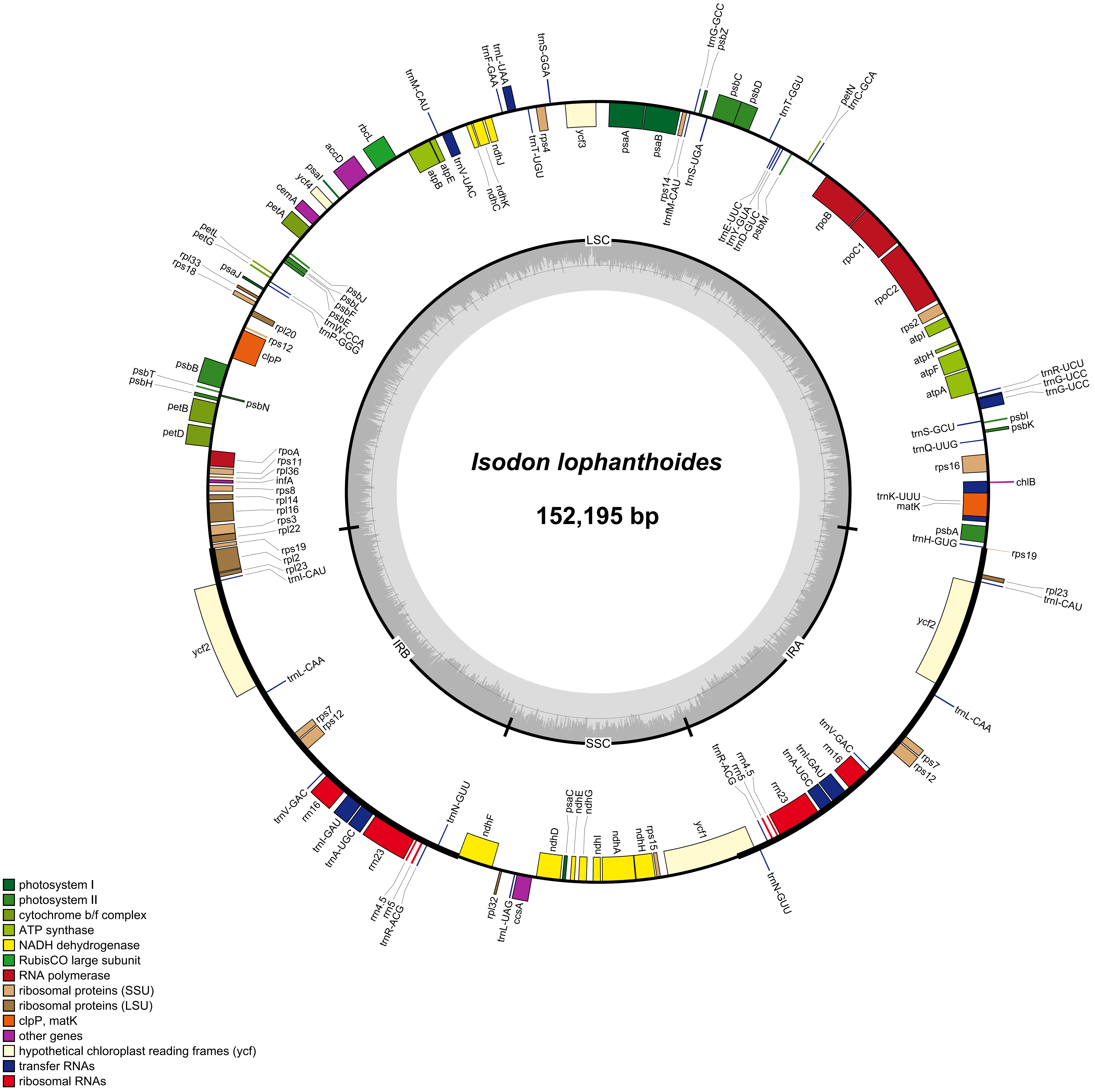

Isodon lophanthoides
152,195 bp
LSC
IRA
SSC
IRB
trnG-GCC
psbZ
psbC
psbD
trnT-GGU
petN
trnC-GCA
trnS-GGA
trnL-UAA
trnF-GAA
trnM-CAU
rbcL
accD
psaI
ycf4
cemA
petA
petL
petG
rpl33
rps18
psaJ
rpl20
rps12
clpP
psbB
psbT
psbH
psbN
petB
petD
rpoA
rps11
rpl36
infA
rps8
rpl14
rpl16
rps3
rpl22
rps19
rpl2
rpl23
trnI-CAU
ycf2
trnL-CAA
rps7
rps12
trnV-GAC
rrn16
trnI-GAU
trnA-UGC
rrn23
rrn4.5
rrn5
trnR-ACG
trnN-GUU
ndhF
rpl32
trnL-UAG
ccsA
ndhD
psaC
ndhE
ndhG
ndhI
ndhA
ndhH
rps15
ycf1
trnN-GUU
trnR-ACG
rrn5
rrn4.5
rrn23
trnA-UGC
trnI-GAU
rrn16
trnV-GAC
rps7
rps12
trnL-CAA
ycf2
rpl23
trnI-CAU
rps19
trnH-GUG
psbA
matK
trnK-UUU
chlB
rps16
trnQ-UUG
trnS-GCU
psbI
psbK
trnR-UCU
trnG-UCC
trnG-UCC
atpA
atpF
atpH
atpI
rps2
rpoC2
rpoC1
rpoB
trnE-UUC
trnY-GUA
trnD-GUC
psbM
trnS-UGA
trnfM-CAU
rps14
psaB
psaA
ycf3
rps4
trnT-UGU
ndhJ
ndhK
ndhC
trnV-UAC
atpE
atpB
psbJ
psbL
psbF
psbE
trnW-CCA
trnP-GGG
photosystem I
photosystem II
cytochrome b/f complex
ATP synthase
NADH dehydrogenase
RubisCO large subunit
RNA polymerase
ribosomal proteins (SSU)
ribosomal proteins (LSU)
clpP, matK
other genes
hypothetical chloroplast reading frames (ycf)
transfer RNAs
ribosomal RNAs

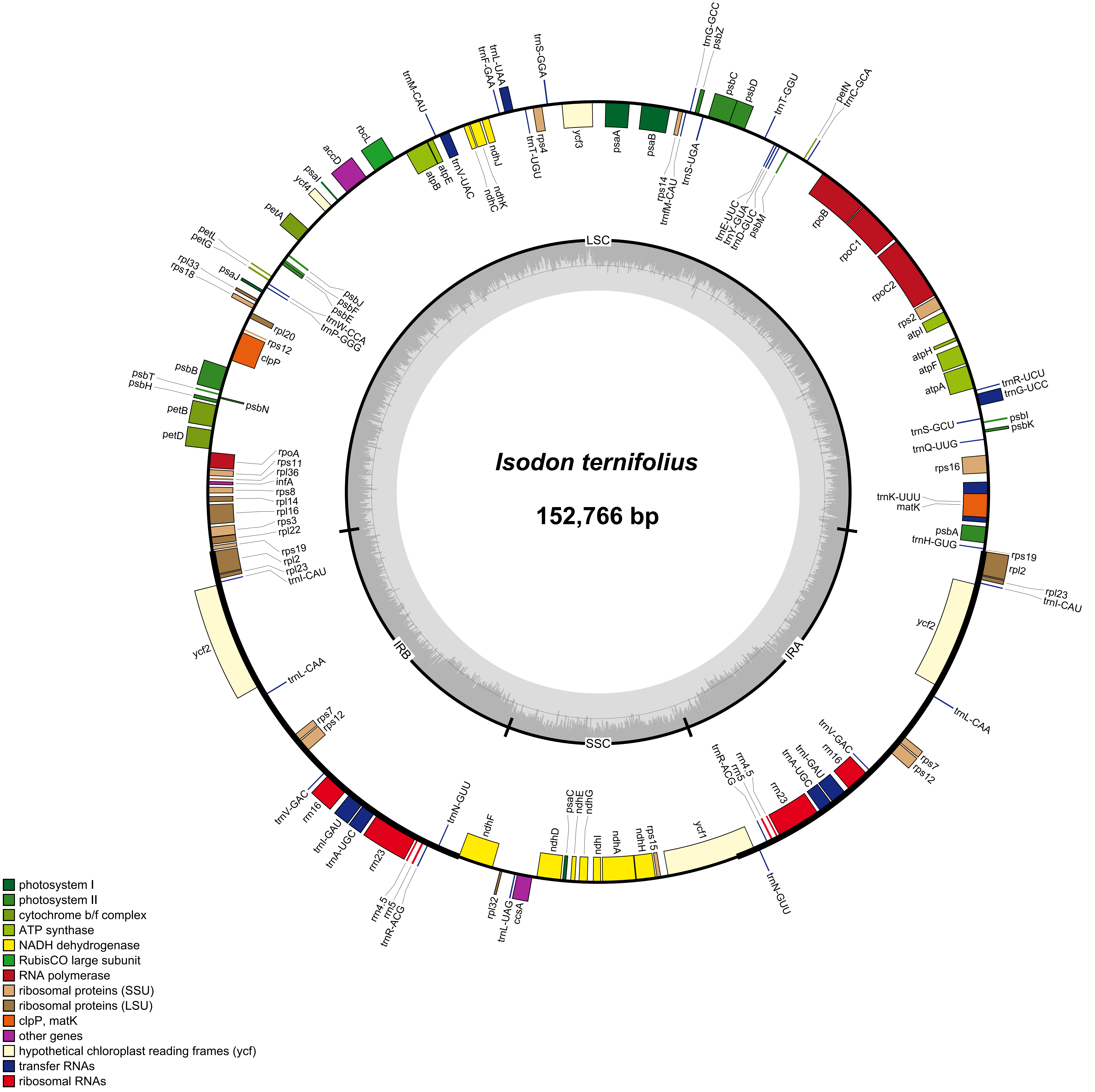

Isodon ternifolius
152,766 bp
LSC
SSC
IRA
IRB
trnG-GCC
psbZ
psbC
psbD
trnT-GGU
petN
trnC-GCA
rpoB
rpoC1
rpoC2
rps2
atpI
atpH
atpF
atpA
trnR-UCU
trnG-UCC
psbI
psbK
trnS-GCU
trnQ-UUG
rps16
trnK-UUU
matK
psbA
trnH-GUG
rps19
rpl2
rpl23
trnI-CAU
ycf2
trnL-CAA
rps7
rps12
trnV-GAC
rrn16
trnI-GAU
trnA-UGC
rrn23
rrn4.5
rrn5
trnR-ACG
trnN-GUU
ycf1
rps15
ndhH
ndhA
ndhI
ndhG
ndhE
psaC
ndhD
ccsA
trnL-UAG
rpl32
ndhF
rpl22
rps3
rpl16
rpl14
rps8
infA
rpl36
rps11
rpoA
petD
petB
psbN
psbH
psbT
psbB
clpP
rpl20
rpl33
rps18
psaJ
trnP-GGG
trnW-CCA
psbE
psbF
psbL
psbJ
petG
petL
petA
ycf4
psaI
accD
rbcL
atpB
atpE
trnV-UAC
trnM-CAU
ndhC
ndhK
ndhJ
trnF-GAA
trnL-UAA
trnT-UGU
trnS-GGA
rps4
ycf3
psaA
psaB
rps14
trnfM-CAU
trnS-UGA
trnE-UUC
trnY-GUA
trnD-GUC
psbM
photosystem I
photosystem II
cytochrome b/f complex
ATP synthase
NADH dehydrogenase
RubisCO large subunit
RNA polymerase
ribosomal proteins (SSU)
ribosomal proteins (LSU)
clpP, matK
other genes
hypothetical chloroplast reading frames (ycf)
transfer RNAs
ribosomal RNAs

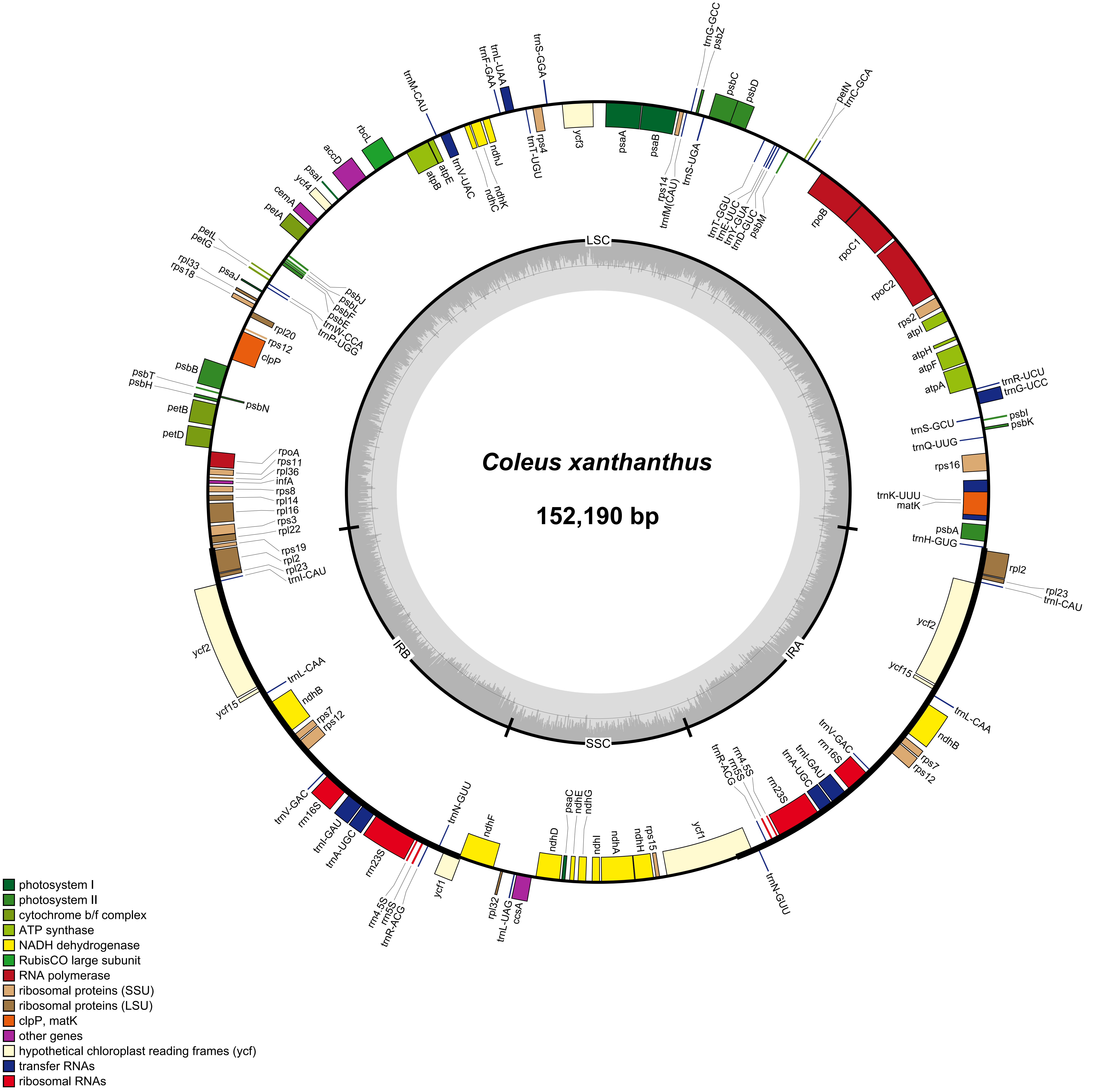

Coleus xanthanthus
152,190 bp
LSC
IRA
IRB
SSC
trnG-GCC
psbZ
psbC
psbD
petN
trnC-GCA
trnS-GGA
trnL-UAA
trnF-GAA
trnM-CAU
rbcL
accD
psaI
ycf4
cemA
petA
petL
petG
rpl33
rps18
psaJ
psbJ
psbL
psbF
psbE
trnW-CCA
trnP-UGG
rpl20
rps12
clpP
psbB
psbT
psbH
psbN
petB
petD
rpoA
rps11
rpl36
infA
rps8
rpl14
rpl16
rps3
rpl22
rps19
rpl2
rpl23
trnI-CAU
ycf2
ycf15
trnL-CAA
ndhB
rps7
trnV-GAC
rrn16S
trnI-GAU
trnA-UGC
rrn23S
rrn4.5S
rrn5S
trnR-ACG
ycf1
trnN-GUU
ndhF
rpl32
trnL-UAG
ccsA
ndhD
psaC
ndhE
ndhG
ndhI
ndhA
ndhH
rps15
atpB
atpE
trnV-UAC
ndhJ
ndhK
ndhC
trnT-UGU
rps4
ycf3
psaA
psaB
rps14
trnfM(CAU)
trnS-UGA
trnT-GGU
trnE-UUC
trnY-GUA
trnD-GUC
psbM
rpoB
rpoC1
rpoC2
rps2
atpI
atpH
atpF
atpA
trnR-UCU
trnG-UCC
psbI
psbK
trnS-GCU
trnQ-UUG
rps16
trnK-UUU
matK
psbA
trnH-GUG
photosystem I
photosystem II
cytochrome b/f complex
ATP synthase
NADH dehydrogenase
RubisCO large subunit
RNA polymerase
ribosomal proteins (SSU)
ribosomal proteins (LSU)
clpP, matK
other genes
hypothetical chloroplast reading frames (ycf)
transfer RNAs
ribosomal RNAs

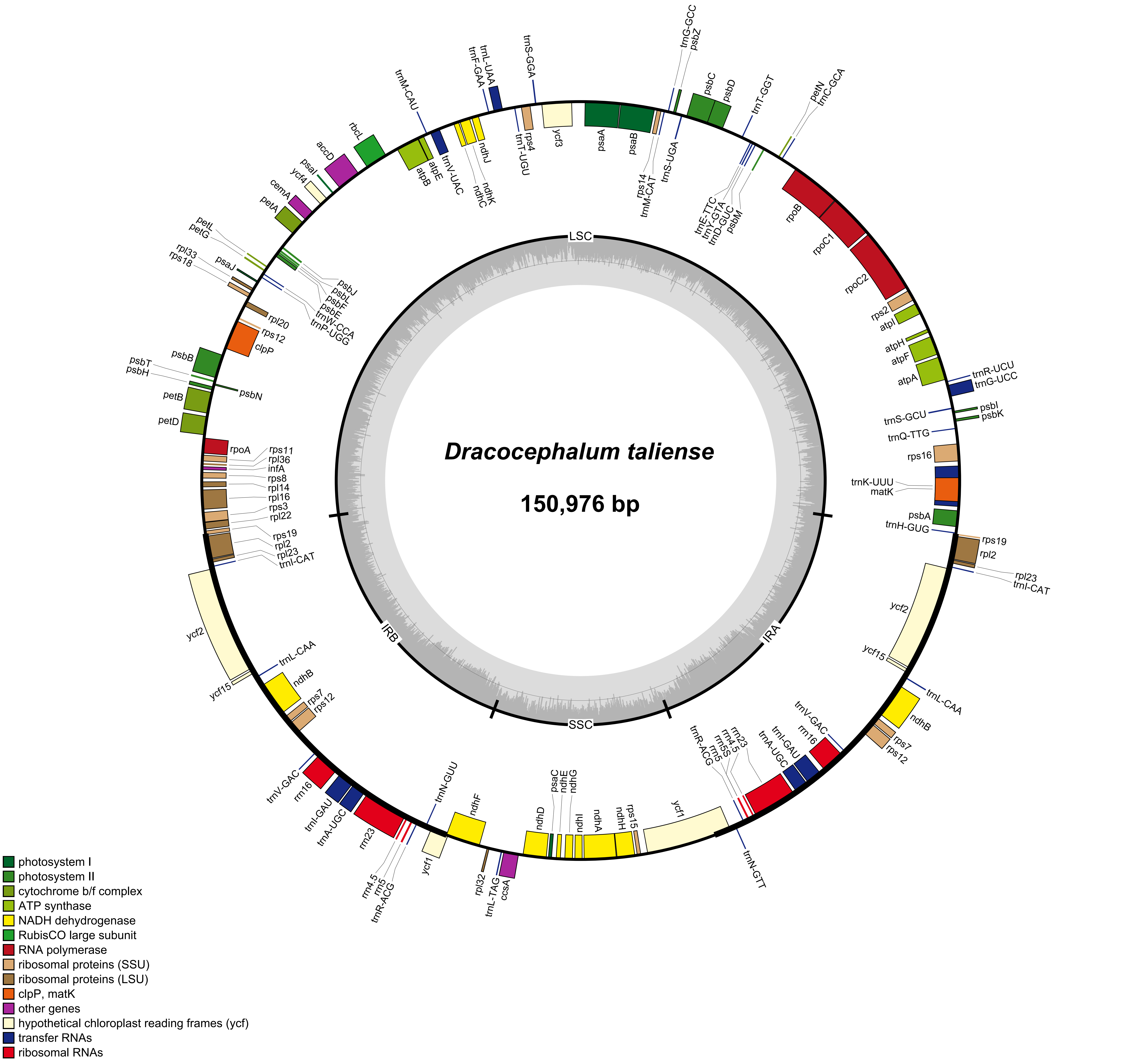

Dracocephalum taliense
150,976 bp
LSC
SSC
IRA
IRB
photosystem I
photosystem II
cytochrome b/f complex
ATP synthase
NADH dehydrogenase
RubisCO large subunit
RNA polymerase
ribosomal proteins (SSU)
ribosomal proteins (LSU)
clpP, matK
other genes
hypothetical chloroplast reading frames (ycf)
transfer RNAs
ribosomal RNAs

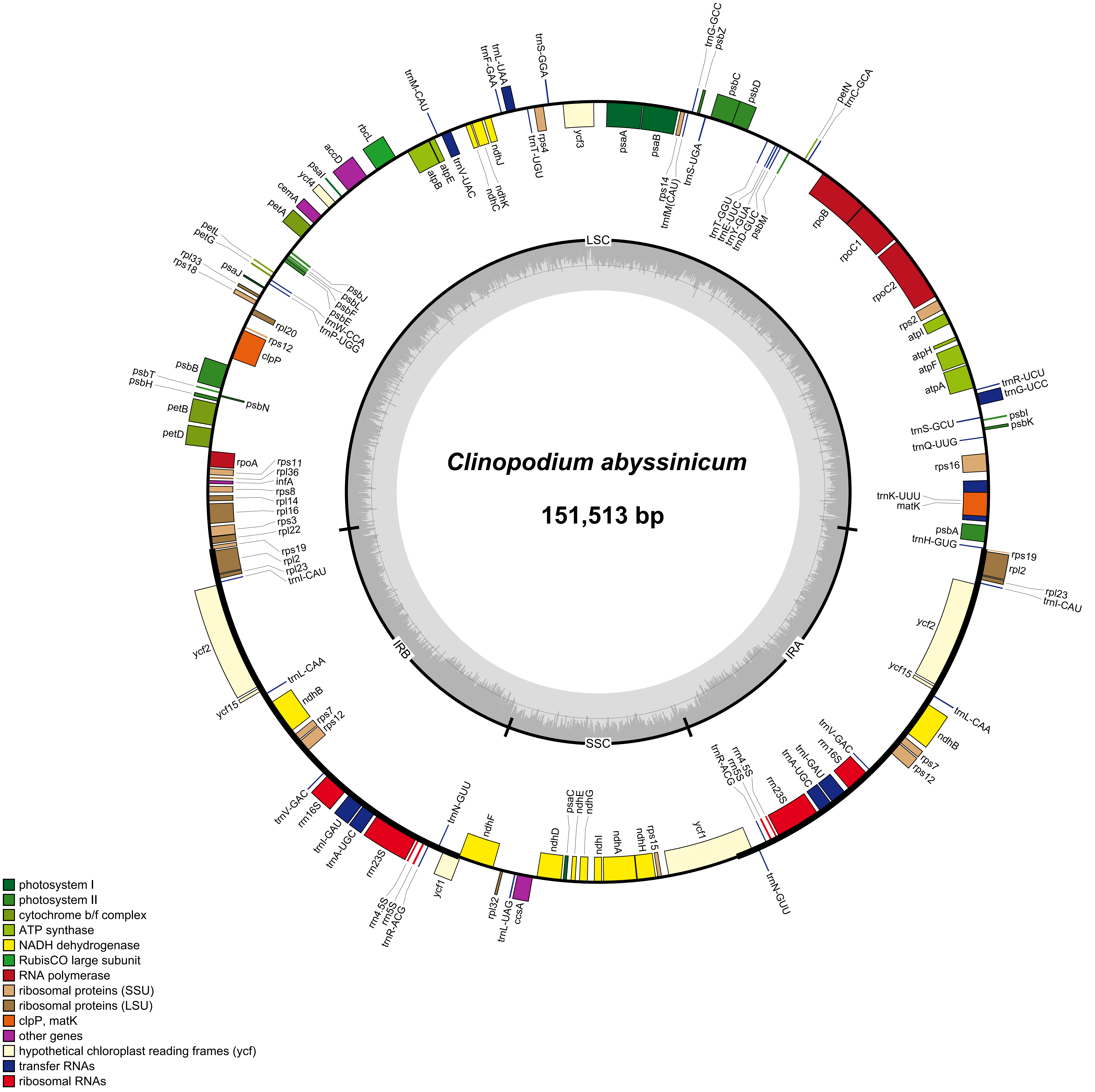
Clinopodium abyssinicum
151,513 bp
LSC
IRA
SSC
IRB
psaA
psaB
ycf3
rps4
trnT-UGU
trnS-GGA
trnL-UAA
trnF-GAA
ndhJ
ndhK
ndhC
trnV-UAC
trnM-CAU
atpE
atpB
rbcL
accD
psaI
ycf4
cemA
petA
petL
petG
psaJ
rpl33
rps18
psbJ
psbL
psbF
psbE
trnW-CCA
trnP-UGG
rpl20
rps12
clpP
psbB
psbT
psbH
psbN
petB
petD
rpoA
rps11
rpl36
infA
rps8
rpl14
rpl16
rps3
rpl22
rps19
rpl2
rpl23
trnI-CAU
ycf2
ycf15
trnL-CAA
ndhB
rps7
trnV-GAC
rrn16S
trnI-GAU
trnA-UGC
rrn23S
rrn4.5S
rrn5S
trnR-ACG
trnN-GUU
ycf1
ndhF
rpl32
trnL-UAG
ccsA
ndhD
psaC
ndhE
ndhG
ndhI
ndhA
ndhH
rps15
trnG-GCC
psbZ
psbC
psbD
rps14
trnfM(CAU)
trnS-UGA
trnT-GGU
trnE-UUC
trnY-GUA
trnD-GUC
psbM
petN
trnC-GCA
rpoB
rpoC1
rpoC2
rps2
atpI
atpH
atpF
atpA
trnR-UCU
trnG-UCC
psbI
psbK
trnS-GCU
trnQ-UUG
rps16
trnK-UUU
matK
psbA
trnH-GUG
photosystem I
photosystem II
cytochrome b/f complex
ATP synthase
NADH dehydrogenase
RubisCO large subunit
RNA polymerase
ribosomal proteins (SSU)
ribosomal proteins (LSU)
clpP, matK
other genes
hypothetical chloroplast reading frames (ycf)
transfer RNAs
ribosomal RNAs

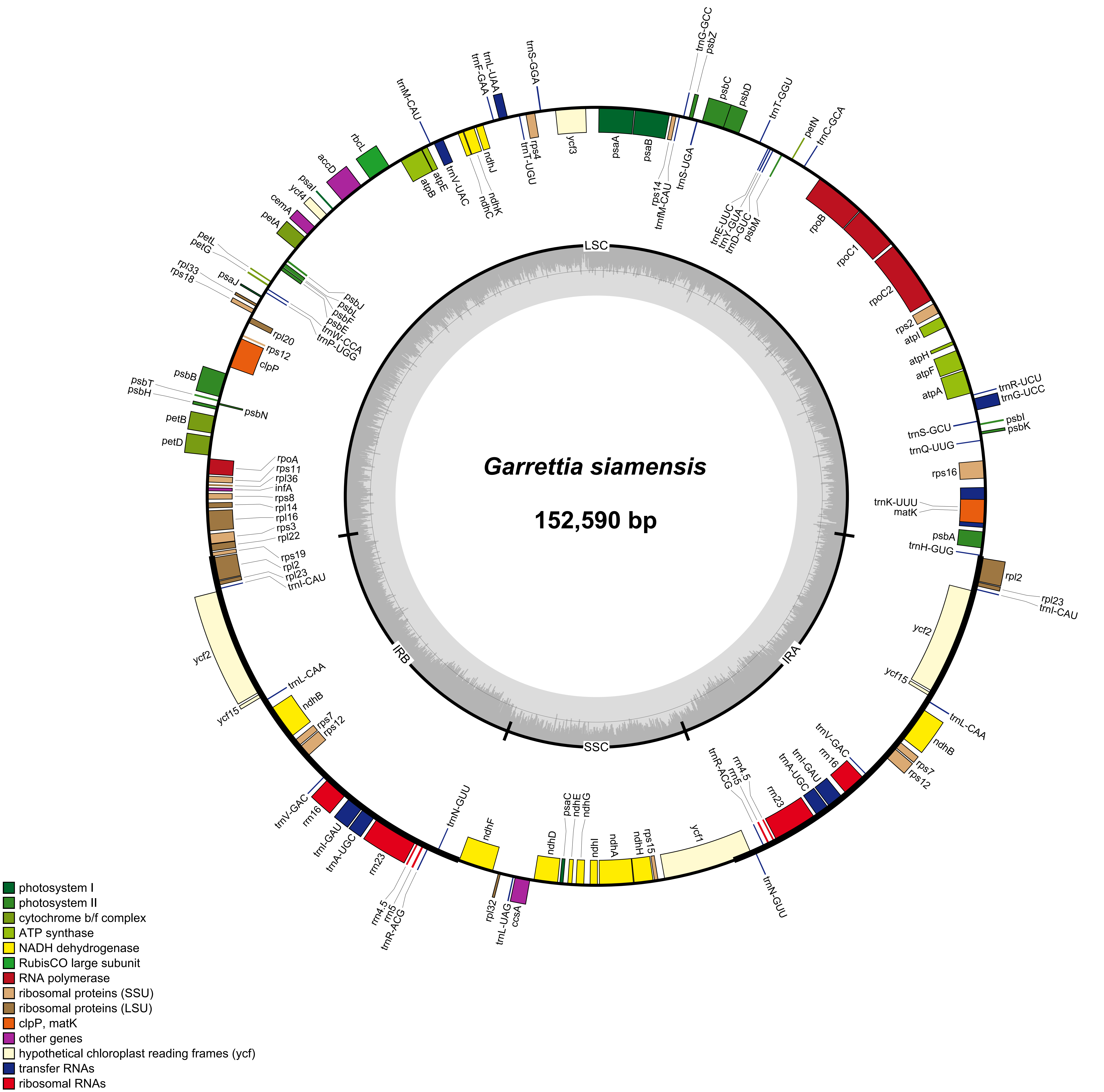

Garrettia siamensis
152,590 bp
LSC
IRA
SSC
IRB
trnG-GCC
psbZ
psbC
psbD
trnT-GGU
petN
trnC-GCA
rpoB
rpoC1
rpoC2
rps2
atpI
atpH
atpF
atpA
trnR-UCU
trnG-UCC
psbI
psbK
trnS-GCU
trnQ-UUG
rps16
trnK-UUU
matK
psbA
trnH-GUG
rpl2
rpl23
trnI-CAU
ycf2
ycf15
trnL-CAA
ndhB
rps7
rps12
trnV-GAC
rrn16
trnI-GAU
trnA-UGC
rrn23
rrn4.5
rrn5
trnR-ACG
trnN-GUU
ycf1
rps15
ndhH
ndhA
ndhI
ndhG
ndhE
psaC
ndhD
ccsA
trnL-UAG
rpl32
ndhF
trnN-GUU
rrn23
trnA-UGC
trnI-GAU
rrn16
trnV-GAC
rrn4.5
rrn5
trnR-ACG
rps12
rps7
ndhB
trnL-CAA
ycf15
ycf2
trnI-CAU
rpl23
rpl2
rps19
rpl22
rps3
rpl16
rpl14
rps8
infA
rpl36
rps11
rpoA
petD
petB
psbN
psbH
psbT
psbB
clpP
rps12
rpl20
trnP-UGG
trnW-CCA
psbE
psbF
psbL
psbJ
rps18
rpl33
psaJ
petG
petL
petA
cemA
ycf4
psaI
accD
rbcL
atpB
atpE
trnV-UAC
trnM-CAU
ndhC
ndhK
ndhJ
trnF-GAA
trnL-UAA
trnT-UGU
rps4
trnS-GGA
ycf3
psaA
psaB
rps14
trnfM-CAU
trnS-UGA
trnE-UUC
trnY-GUA
trnD-GUC
psbM
photosystem I
photosystem II
cytochrome b/f complex
ATP synthase
NADH dehydrogenase
RubisCO large subunit
RNA polymerase
ribosomal proteins (SSU)
ribosomal proteins (LSU)
clpP, matK
other genes
hypothetical chloroplast reading frames (ycf)
transfer RNAs
ribosomal RNAs

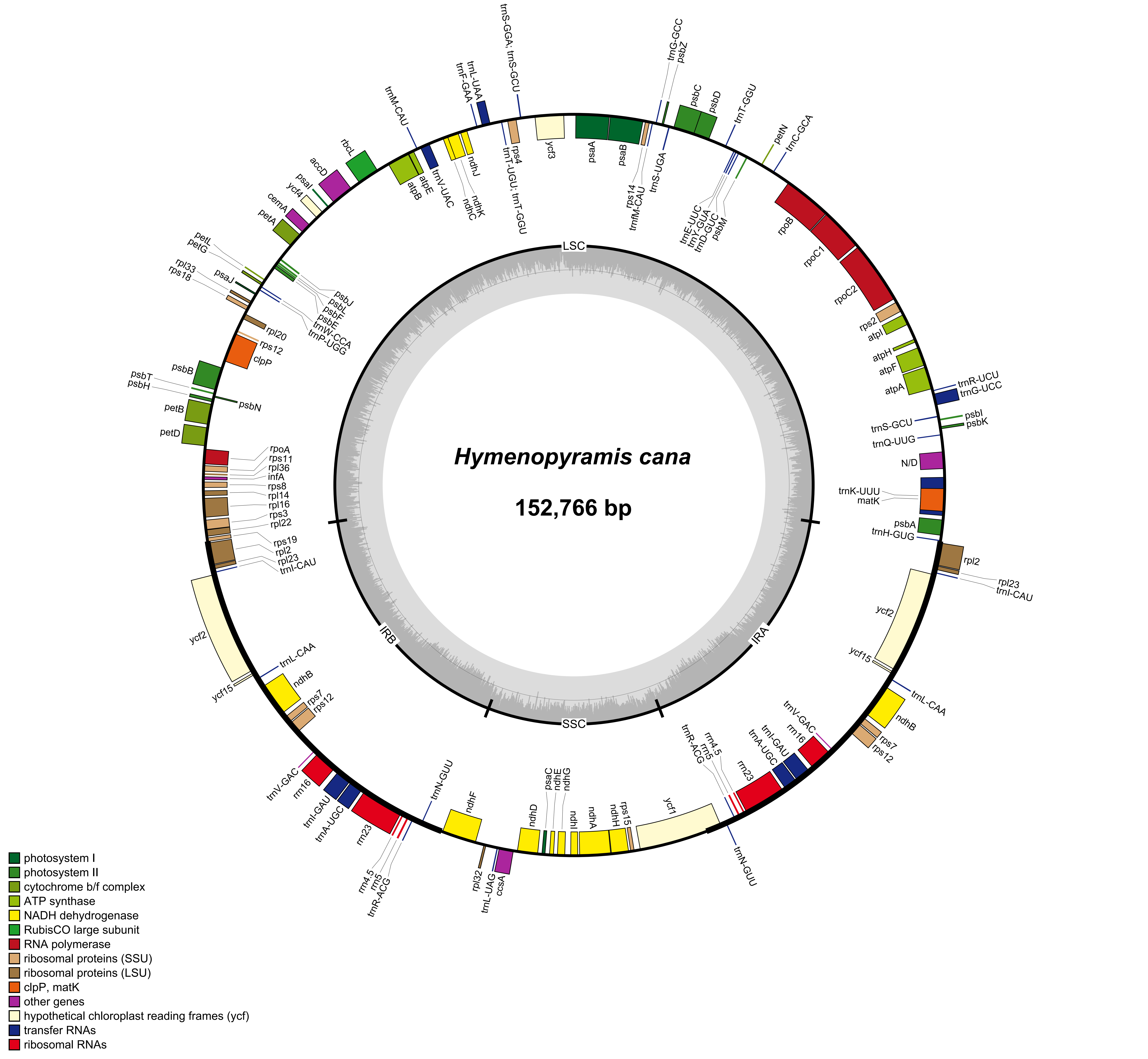

Hymenopyramis cana
152,766 bp
LSC
IRA
IRB
SSC
trnS-GGA; trnS-GCU
trnL-UAA
trnF-GAA
trnM-CAU
rbcL
accD
psaI
ycf4
cemA
petA
petL
petG
rpl33
rps18
psaJ
rpl20
rps12
clpP
psbB
psbT
psbH
psbN
petB
petD
rpoA
rps11
rpl36
infA
rps8
rpl14
rpl16
rps3
rpl22
rps19
rpl2
rpl23
trnI-CAU
ycf2
ycf15
trnL-CAA
ndhB
rps7
trnV-GAC
rrn16
trnI-GAU
trnA-UGC
rrn23
rrn4.5
rrn5
trnR-ACG
trnN-GUU
ndhF
rpl32
trnL-UAG
ccsA
ndhD
psaC
ndhE
ndhG
ndhI
ndhA
ndhH
rps15
ycf1
psbJ
psbL
psbF
psbE
trnW-CCA
trnP-UGG
atpB
atpE
trnV-UAC
ndhC
ndhK
ndhJ
trnT-UGU; trnT-GGU
rps4
ycf3
psaA
psaB
rps14
trnfM-CAU
trnS-UGA
trnG-GCC
psbZ
psbC
psbD
trnT-GGU
trnE-UUC
trnY-GUA
trnD-GUC
psbM
petN
trnC-GCA
rpoB
rpoC1
rpoC2
rps2
atpI
atpH
atpF
atpA
trnR-UCU
trnG-UCC
trnS-GCU
trnQ-UUG
psbI
psbK
N/D
trnK-UUU
matK
psbA
trnH-GUG
photosystem I
photosystem II
cytochrome b/f complex
ATP synthase
NADH dehydrogenase
RubisCO large subunit
RNA polymerase
ribosomal proteins (SSU)
ribosomal proteins (LSU)
clpP, matK
other genes
hypothetical chloroplast reading frames (ycf)
transfer RNAs
ribosomal RNAs

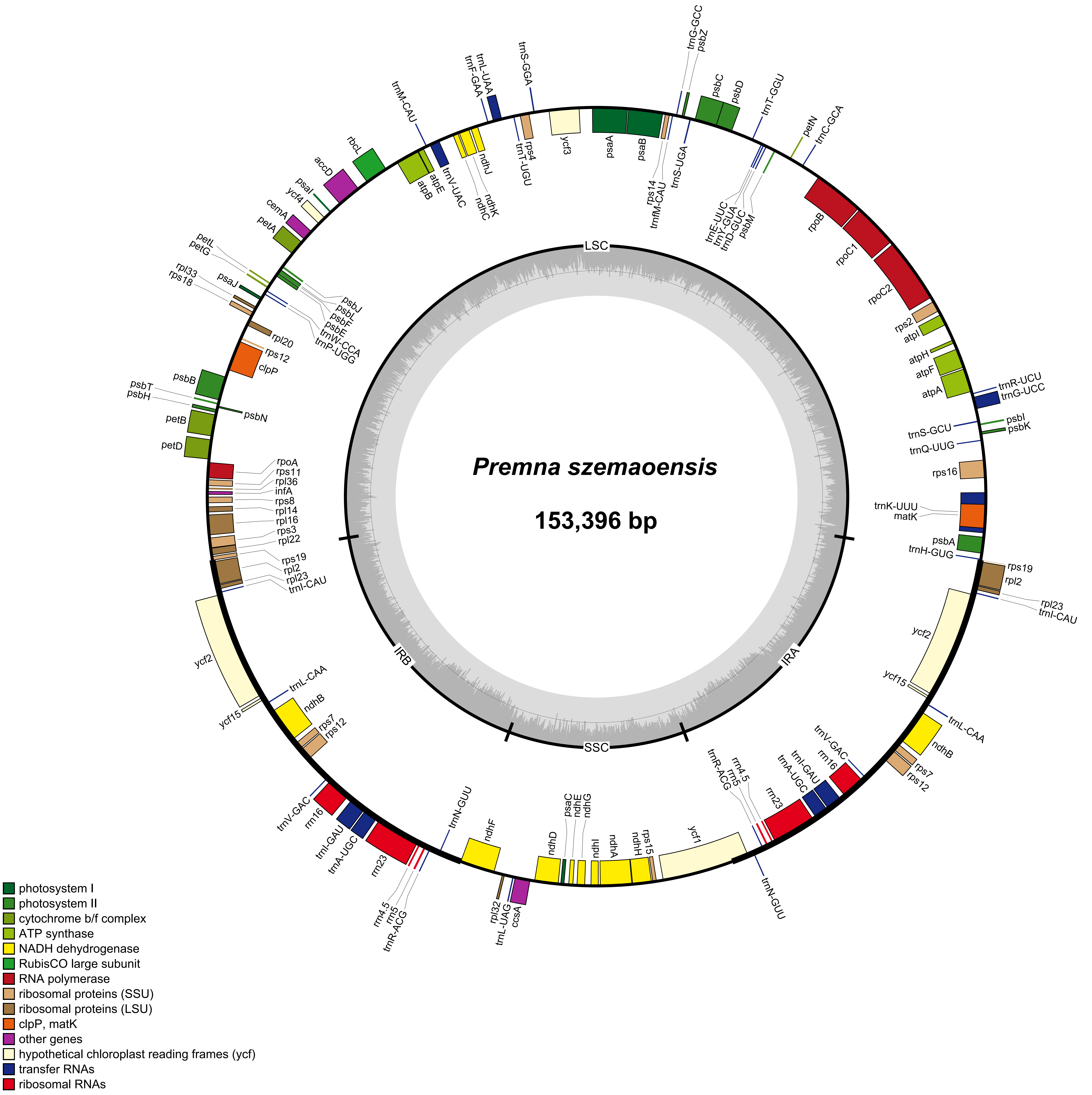
Premna szemaoensis
153,396 bp
LSC
SSC
IRA
IRB
psaA
psaB
ycf3
rps4
trnS-GGA
trnT-UGU
trnL-UAA
trnF-GAA
ndhJ
ndhK
ndhC
trnV-UAC
trnM-CAU
atpE
atpB
rbcL
accD
psaI
ycf4
cemA
petA
petL
petG
psbJ
psbL
psbF
psbE
trnW-CCA
trnP-UGG
psaJ
rpl33
rps18
rpl20
rps12
clpP
psbB
psbT
psbH
psbN
petB
petD
rpoA
rps11
rpl36
infA
rps8
rpl14
rpl16
rps3
rpl22
rps19
rpl2
rpl23
trnI-CAU
ycf2
ycf15
trnL-CAA
ndhB
rps7
trnV-GAC
rrn16
trnI-GAU
trnA-UGC
rrn23
rrn4.5
rrn5
trnR-ACG
trnN-GUU
ndhF
rpl32
trnL-UAG
ccsA
ndhD
psaC
ndhE
ndhG
ndhI
ndhA
ndhH
rps15
ycf1
trnG-GCC
psbZ
psbC
psbD
trnT-GGU
petN
trnC-GCA
rps14
trnfM-CAU
trnS-UGA
trnE-UUC
trnY-GUA
trnD-GUC
psbM
rpoB
rpoC1
rpoC2
rps2
atpI
atpH
atpF
atpA
trnR-UCU
trnG-UCC
psbI
psbK
trnS-GCU
trnQ-UUG
rps16
trnK-UUU
matK
psbA
trnH-GUG
photosystem I
photosystem II
cytochrome b/f complex
ATP synthase
NADH dehydrogenase
RubisCO large subunit
RNA polymerase
ribosomal proteins (SSU)
ribosomal proteins (LSU)
clpP, matK
other genes
hypothetical chloroplast reading frames (ycf)
transfer RNAs
ribosomal RNAs

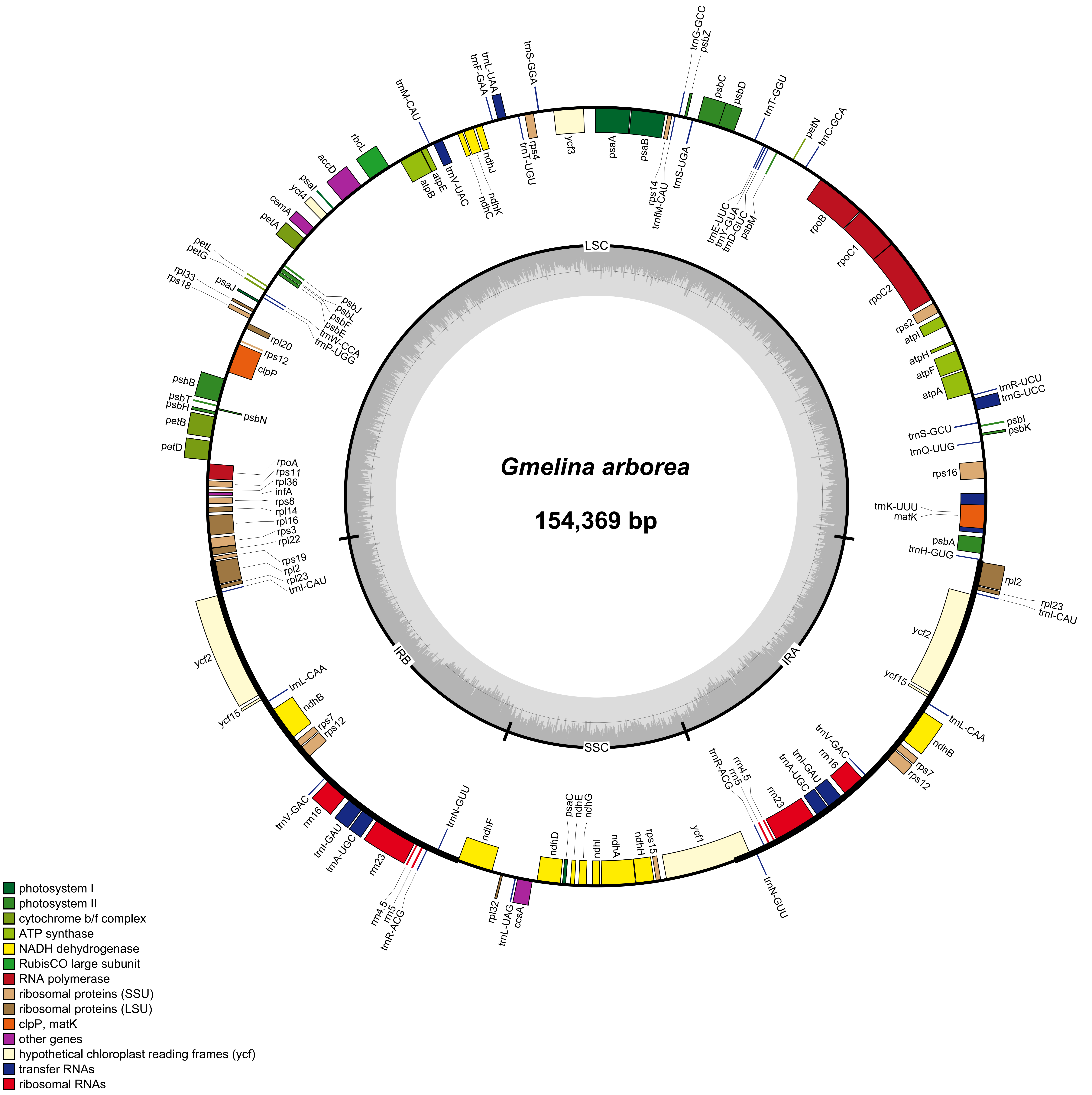

Gmelina arborea
154,369 bp
LSC
SSC
IRA
IRB
photosystem I
photosystem II
cytochrome b/f complex
ATP synthase
NADH dehydrogenase
RubisCO large subunit
RNA polymerase
ribosomal proteins (SSU)
ribosomal proteins (LSU)
clpP, matK
other genes
hypothetical chloroplast reading frames (ycf)
transfer RNAs
ribosomal RNAs

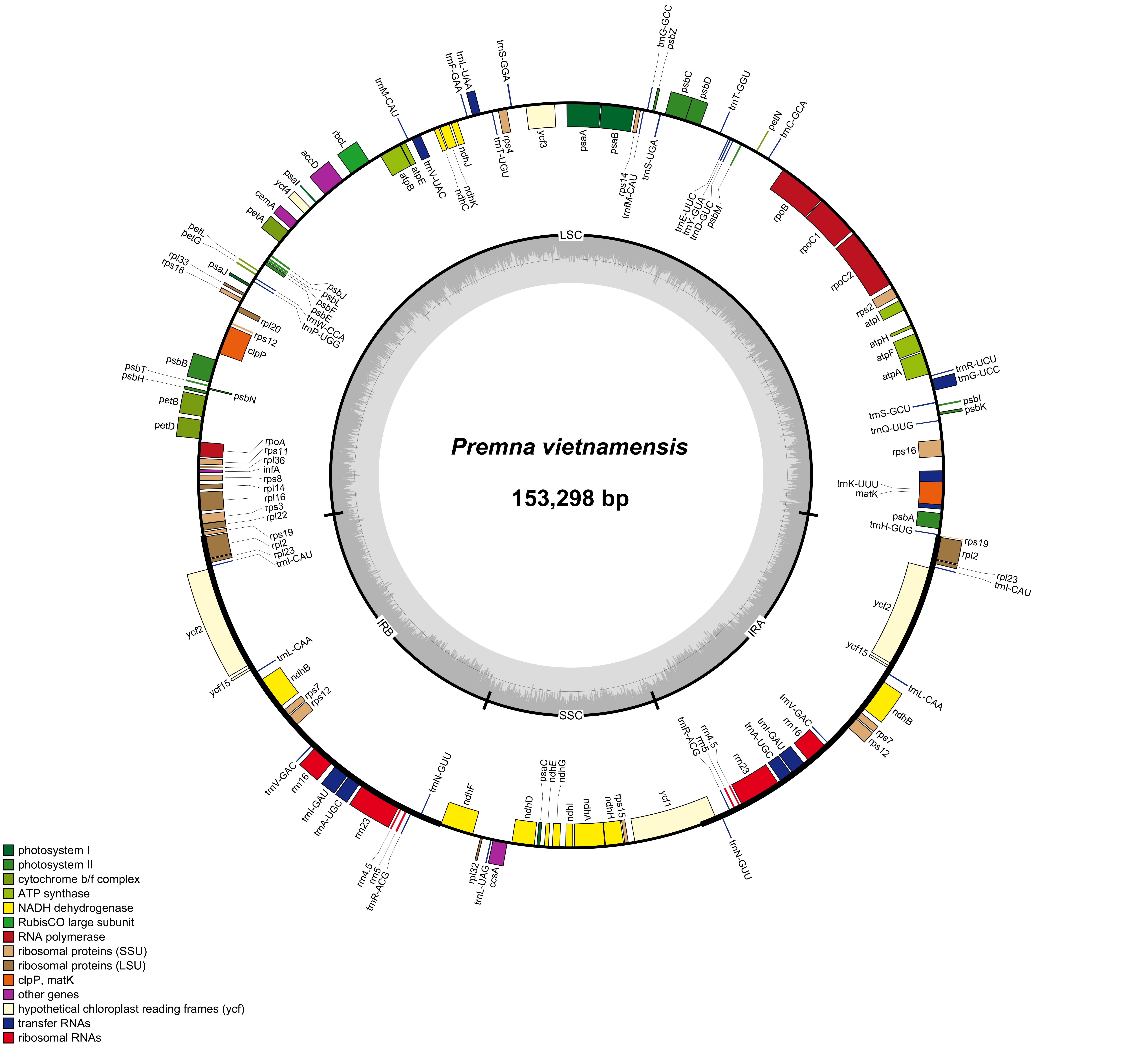
Premna vietnamensis
153,298 bp
LSC
SSC
IRA
IRB
trnG-GCC
psbZ
psbC
psbD
trnT-GGU
petN
trnC-GCA
rpoB
rpoC1
rpoC2
rps2
atpI
atpH
atpF
atpA
trnR-UCU
trnG-UCC
psbI
psbK
trnS-GCU
trnQ-UUG
rps16
trnK-UUU
matK
psbA
trnH-GUG
rps19
rpl2
rpl23
trnI-CAU
ycf2
ycf15
trnL-CAA
ndhB
rps7
rps12
trnV-GAC
rrn16
trnI-GAU
trnA-UGC
rrn23
rrn4.5
rrn5
trnR-ACG
trnN-GUU
ycf1
rps15
ndhH
ndhA
ndhI
ndhG
ndhE
psaC
ndhD
ndhF
rpl32
trnL-UAG
ccsA
trnS-GGA
trnL-UAA
trnF-GAA
trnM-CAU
rps4
trnT-UGU
ycf3
psaA
psaB
rps14
trnfM-CAU
trnS-UGA
trnE-UUC
trnY-GUA
trnD-GUC
psbM
ndhJ
ndhK
ndhC
trnV-UAC
atpE
atpB
rbcL
accD
psaI
ycf4
cemA
petA
petL
petG
psaJ
rpl33
rps18
psbJ
psbL
psbF
psbE
trnW-CCA
trnP-UGG
rpl20
rps12
clpP
psbB
psbT
psbH
psbN
petB
petD
rpoA
rps11
rpl36
infA
rps8
rpl14
rpl16
rps3
rpl22
photosystem I
photosystem II
cytochrome b/f complex
ATP synthase
NADH dehydrogenase
RubisCO large subunit
RNA polymerase
ribosomal proteins (SSU)
ribosomal proteins (LSU)
clpP, matK
other genes
hypothetical chloroplast reading frames (ycf)
transfer RNAs
ribosomal RNAs

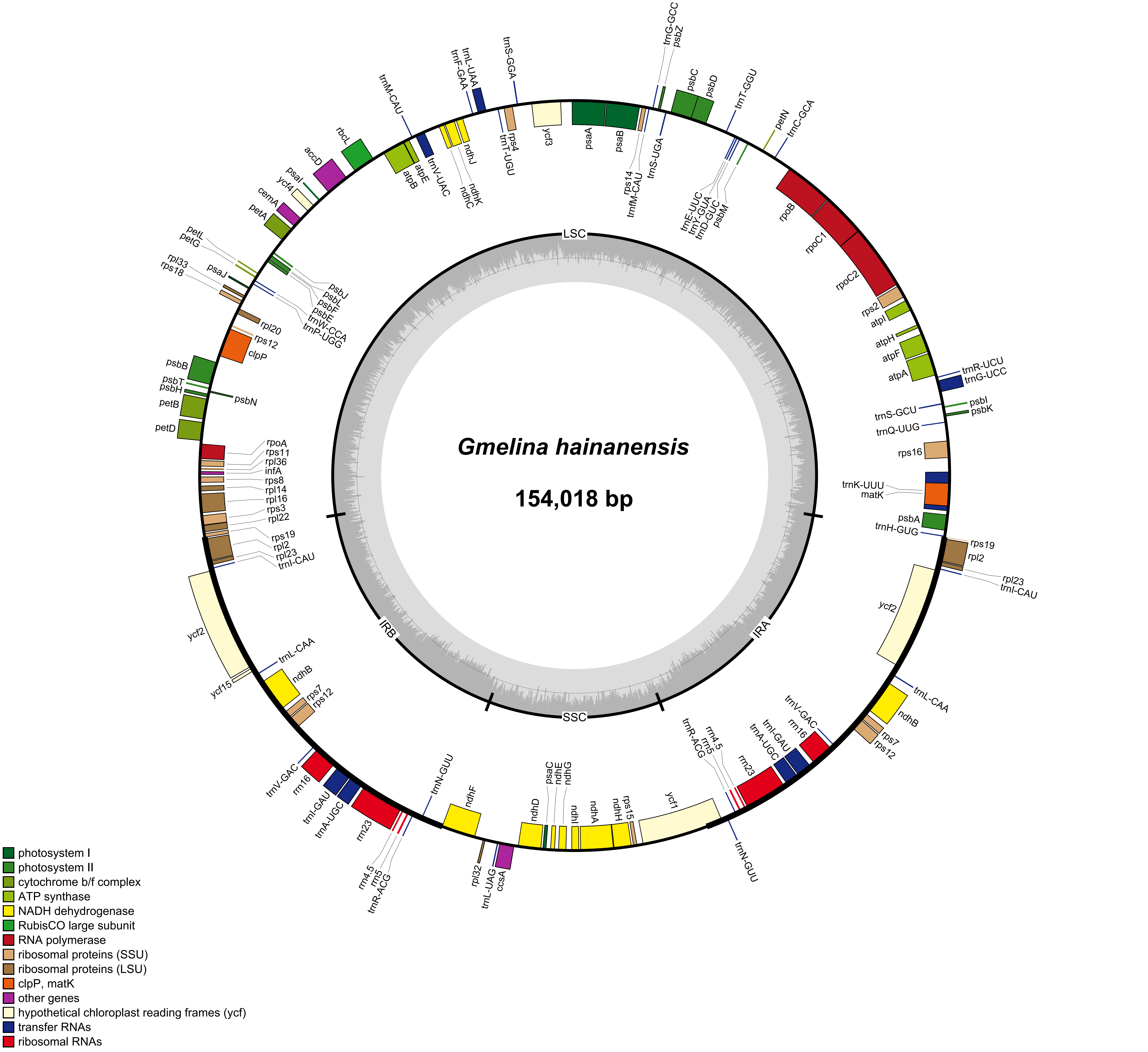

Gmelina hainanensis
154,018 bp
LSC
SSC
IRA
IRB
photosystem I
photosystem II
cytochrome b/f complex
ATP synthase
NADH dehydrogenase
RubisCO large subunit
RNA polymerase
ribosomal proteins (SSU)
ribosomal proteins (LSU)
clpP, matK
other genes
hypothetical chloroplast reading frames (ycf)
transfer RNAs
ribosomal RNAs

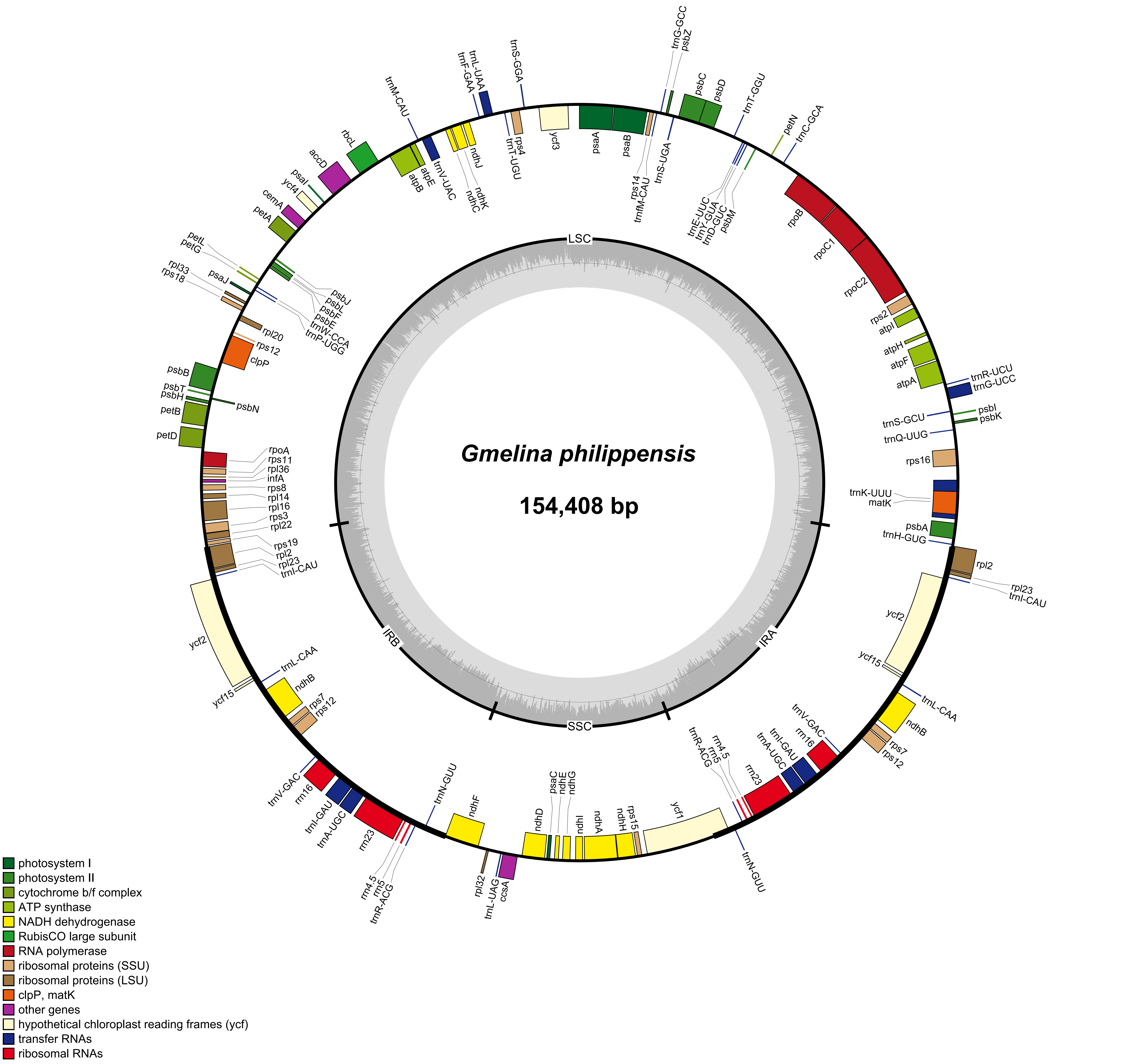

Gmelina philippensis
154,408 bp
LSC
IRA
SSC
IRB
trnG-GCC
psbZ
psbC
psbD
trnT-GGU
petN
trnC-GCA
rpoB
rpoC1
rpoC2
rps2
atpI
atpH
atpF
atpA
trnR-UCU
trnG-UCC
psbI
psbK
trnS-GCU
trnQ-UUG
rps16
trnK-UUU
matK
psbA
trnH-GUG
rpl2
rpl23
trnI-CAU
ycf2
ycf15
trnL-CAA
ndhB
rps7
rps12
trnV-GAC
rrn16
trnI-GAU
trnA-UGC
rrn23
rrn4.5
rrn5
trnR-ACG
trnN-GUU
ycf1
rps15
ndhH
ndhA
ndhI
ndhG
ndhE
psaC
ndhD
ccsA
trnL-UAG
rpl32
ndhF
rpoA
rps11
rpl36
infA
rps8
rpl14
rpl16
rps3
rpl22
rps19
petD
petB
psbH
psbT
psbN
psbB
clpP
rpl20
rpl33
rps18
psaJ
petL
petG
psbJ
psbL
psbF
psbE
trnW-CCA
trnP-UGG
petA
cemA
ycf4
psaI
accD
rbcL
atpB
atpE
trnM-CAU
trnV-UAC
ndhC
ndhK
ndhJ
trnF-GAA
trnL-UAA
trnT-UGU
rps4
trnS-GGA
ycf3
psaA
psaB
rps14
trnfM-CAU
trnS-UGA
trnE-UUC
trnY-GUA
trnD-GUC
psbM
photosystem I
photosystem II
cytochrome b/f complex
ATP synthase
NADH dehydrogenase
RubisCO large subunit
RNA polymerase
ribosomal proteins (SSU)
ribosomal proteins (LSU)
clpP, matK
other genes
hypothetical chloroplast reading frames (ycf)
transfer RNAs
ribosomal RNAs

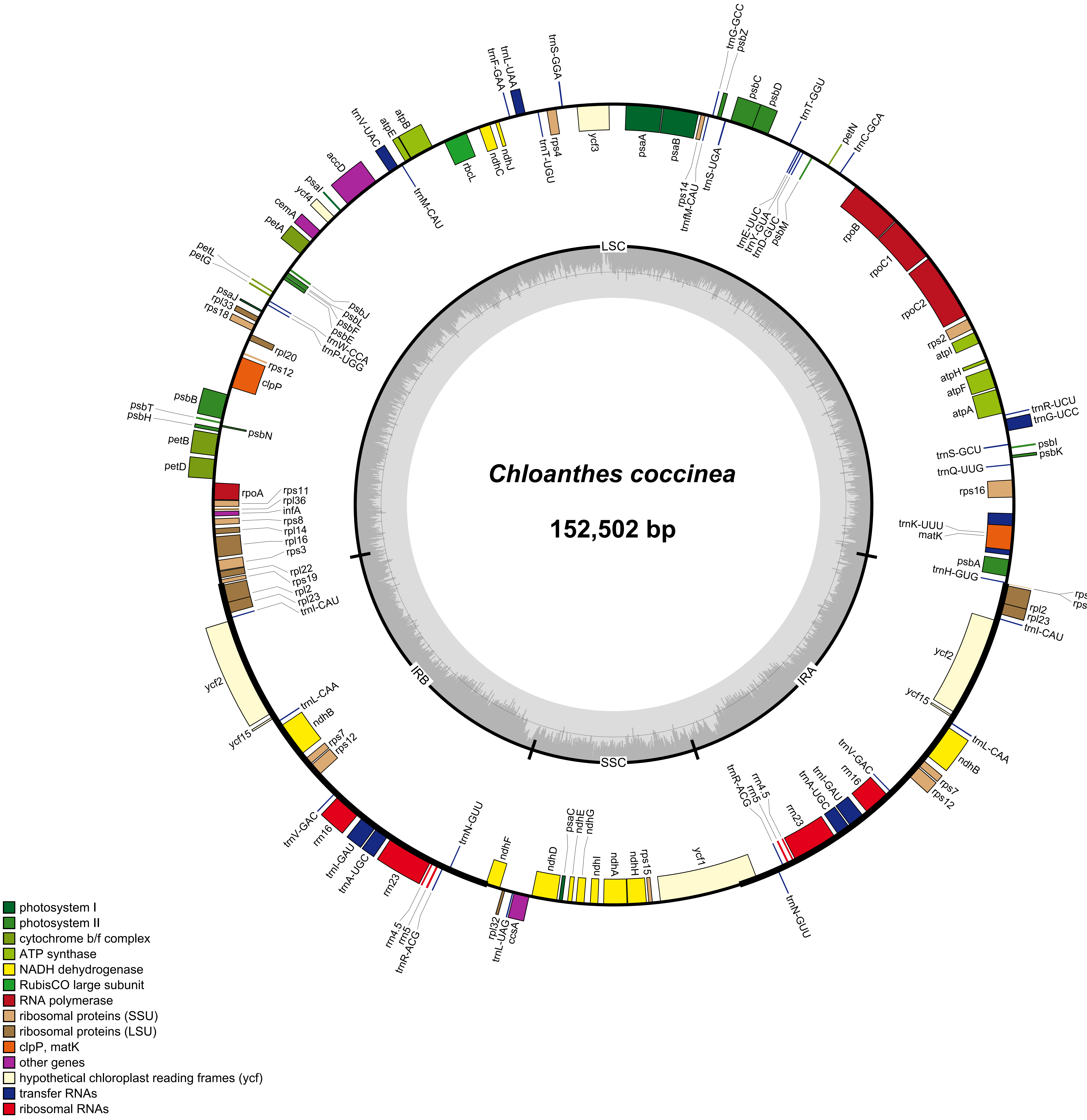
Chloanthes coccinea
152,502 bp
LSC
IRA
SSC
IRB
trnG-GCC
psbZ
psbC
psbD
trnT-GGU
petN
trnC-GCA
trnS-GGA
trnL-UAA
trnF-GAA
rps4
trnT-UGU
ycf3
psaA
psaB
rps14
trnfM-CAU
trnS-UGA
trnE-UUC
trnY-GUA
trnD-GUC
psbM
rpoB
rpoC1
rpoC2
rps2
atpI
atpH
atpF
atpA
trnR-UCU
trnG-UCC
psbI
psbK
trnS-GCU
trnQ-UUG
rps16
trnK-UUU
matK
psbA
trnH-GUG
rps19
rpl2
rpl23
trnI-CAU
ycf2
ycf15
trnL-CAA
ndhB
rps7
rps12
trnV-GAC
rrn16
trnI-GAU
trnA-UGC
rrn23
rrn4.5
rrn5
trnR-ACG
trnN-GUU
ycf1
rps15
ndhH
ndhA
ndhI
ndhG
ndhE
psaC
ndhD
ccsA
trnL-UAG
rpl32
ndhF
atpB
atpE
trnV-UAC
trnM-CAU
rbcL
ndhC
ndhJ
accD
psaI
ycf4
cemA
petA
petL
petG
psbJ
psbL
psbF
psbE
trnW-CCA
trnP-UGG
psaJ
rpl33
rps18
rpl20
clpP
psbB
psbT
psbH
psbN
petB
petD
rpoA
rps11
rpl36
infA
rps8
rpl14
rpl16
rps3
rpl22
photosystem I
photosystem II
cytochrome b/f complex
ATP synthase
NADH dehydrogenase
RubisCO large subunit
RNA polymerase
ribosomal proteins (SSU)
ribosomal proteins (LSU)
clpP, matK
other genes
hypothetical chloroplast reading frames (ycf)
transfer RNAs
ribosomal RNAs

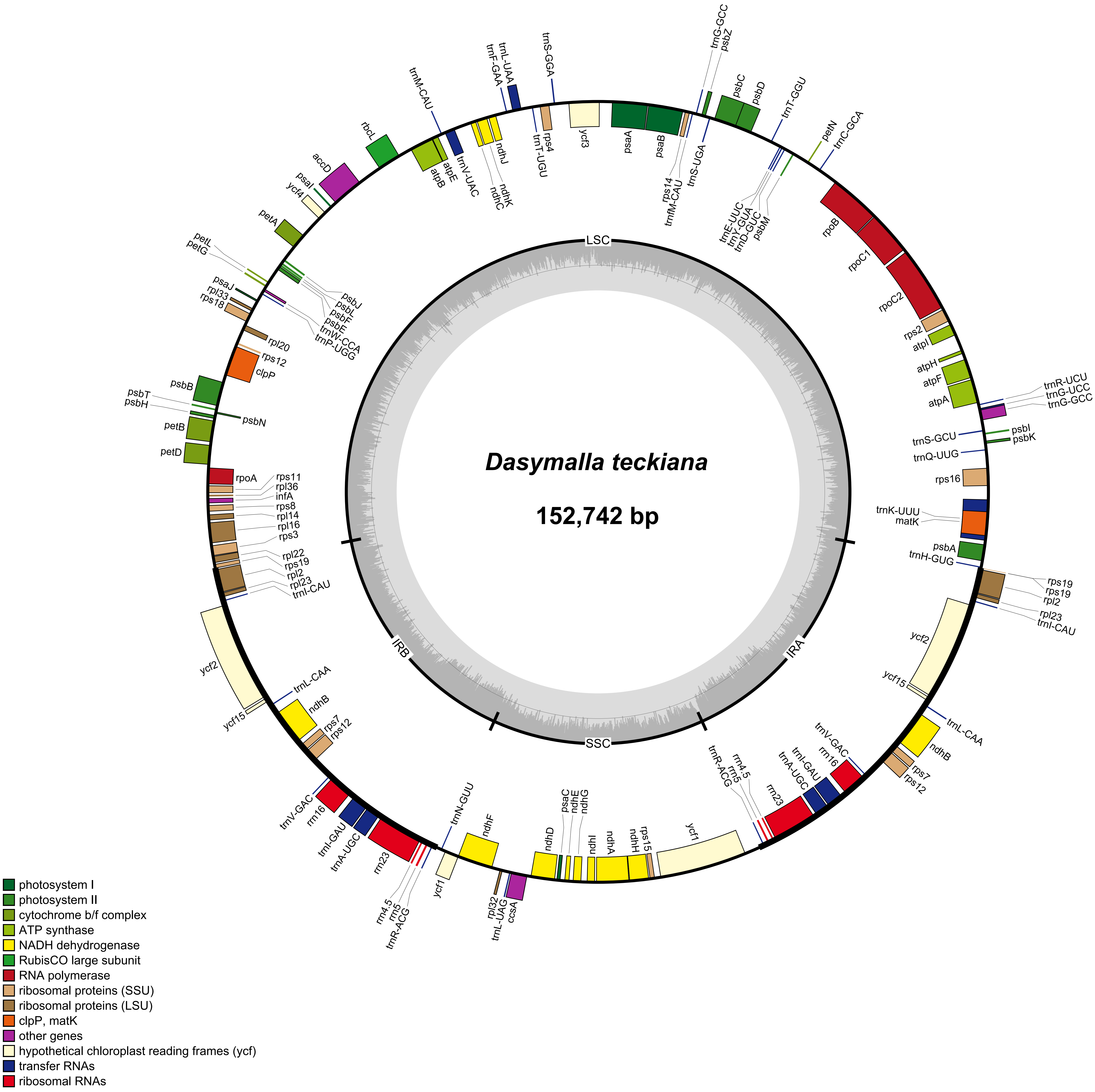

Dasymalla teckiana
152,742 bp
LSC
IRA
SSC
IRB
trnG-GCC
psbZ
psbC
psbD
trnT-GGU
petN
trnC-GCA
rpoB
rpoC1
rpoC2
rps2
atpI
atpH
atpF
atpA
trnR-UCU
trnG-UCC
trnG-GCC
psbI
psbK
trnS-GCU
trnQ-UUG
rps16
trnK-UUU
matK
psbA
trnH-GUG
rps19
rps19
rpl2
rpl23
trnI-CAU
ycf2
ycf15
trnL-CAA
ndhB
rps7
rps12
trnV-GAC
rrn16
trnI-GAU
trnA-UGC
rrn23
rrn4.5
rrn5
trnR-ACG
ycf1
rps15
ndhH
ndhA
ndhI
ndhG
ndhE
psaC
ndhD
ccsA
trnL-UAG
rpl32
ndhF
trnN-GUU
ycf1
trnR-ACG
rrn5
rrn4.5
rrn23
trnA-UGC
trnI-GAU
rrn16
trnV-GAC
rps12
rps7
ndhB
ycf15
trnL-CAA
ycf2
trnI-CAU
rpl23
rpl2
rps19
rpl22
rps3
rpl16
rpl14
rps8
infA
rpl36
rps11
rpoA
petD
petB
psbH
psbT
psbB
psbN
clpP
rps12
rpl20
rps18
rpl33
psaJ
trnP-UGG
trnW-CCA
psbE
psbF
psbL
psbJ
petG
petL
petA
ycf4
psaI
accD
rbcL
atpB
atpE
trnV-UAC
trnM-CAU
ndhC
ndhK
ndhJ
trnF-GAA
trnL-UAA
trnT-UGU
rps4
trnS-GGA
ycf3
psaA
psaB
rps14
trnfM-CAU
trnS-UGA
trnE-UUC
trnY-GUA
trnD-GUC
psbM
photosystem I
photosystem II
cytochrome b/f complex
ATP synthase
NADH dehydrogenase
RubisCO large subunit
RNA polymerase
ribosomal proteins (SSU)
ribosomal proteins (LSU)
clpP, matK
other genes
hypothetical chloroplast reading frames (ycf)
transfer RNAs
ribosomal RNAs

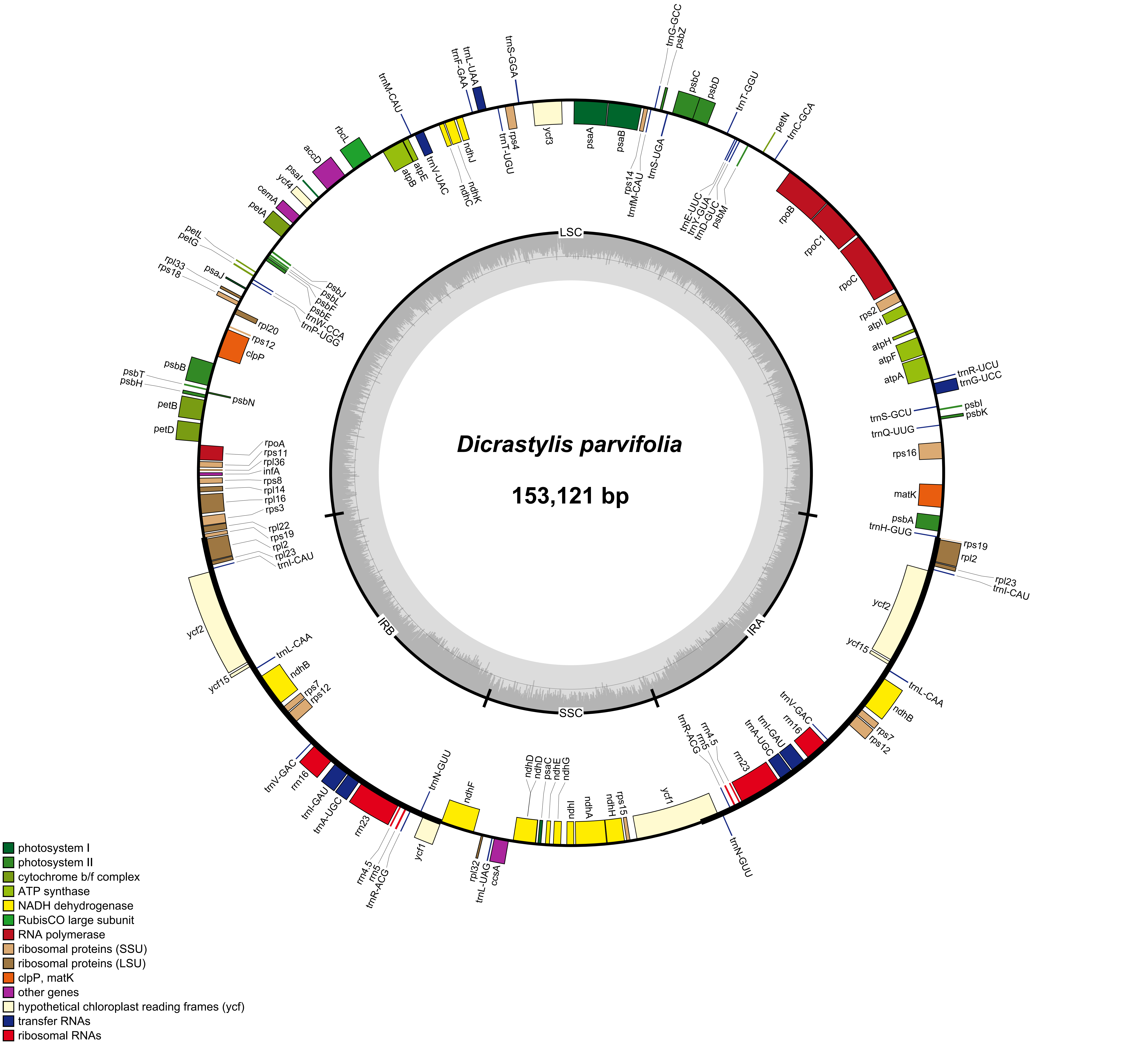

Dicrastylis parvifolia
153,121 bp
LSC
IRA
SSC
IRB
trnG-GCC
psbZ
psbC
psbD
trnT-GGU
petN
trnC-GCA
rpoB
rpoC1
rpoC
rps2
atpI
atpH
atpF
atpA
trnR-UCU
trnG-UCC
psbI
psbK
trnS-GCU
trnQ-UUG
rps16
matK
psbA
trnH-GUG
rps19
rpl2
rpl23
trnI-CAU
ycf2
ycf15
trnL-CAA
ndhB
rps7
rps12
trnV-GAC
rrn16
trnI-GAU
trnA-UGC
rrn23
rrn4.5
rrn5
trnR-ACG
trnN-GUU
ycf1
rps15
ndhH
ndhA
ndhI
ndhG
ndhE
psaC
ndhD
ndhF
ccsA
trnL-UAG
rpl32
psbE
trnW-CCA
trnP-UGG
psbJ
psbL
psbF
rpl20
clpP
psbB
psbT
psbH
psbN
petB
petD
rpoA
rps11
rpl36
infA
rps8
rpl14
rpl16
rps3
rpl22
petL
petG
rpl33
rps18
psaJ
petA
cemA
ycf4
psaI
accD
rbcL
atpB
atpE
trnM-CAU
trnV-UAC
ndhC
ndhK
ndhJ
trnF-GAA
trnL-UAA
trnT-UGU
trnS-GGA
rps4
ycf3
psaA
psaB
rps14
trnfM-CAU
trnS-UGA
trnE-UUC
trnY-GUA
trnD-GUC
psbM
photosystem I
photosystem II
cytochrome b/f complex
ATP synthase
NADH dehydrogenase
RubisCO large subunit
RNA polymerase
ribosomal proteins (SSU)
ribosomal proteins (LSU)
clpP, matK
other genes
hypothetical chloroplast reading frames (ycf)
transfer RNAs
ribosomal RNAs

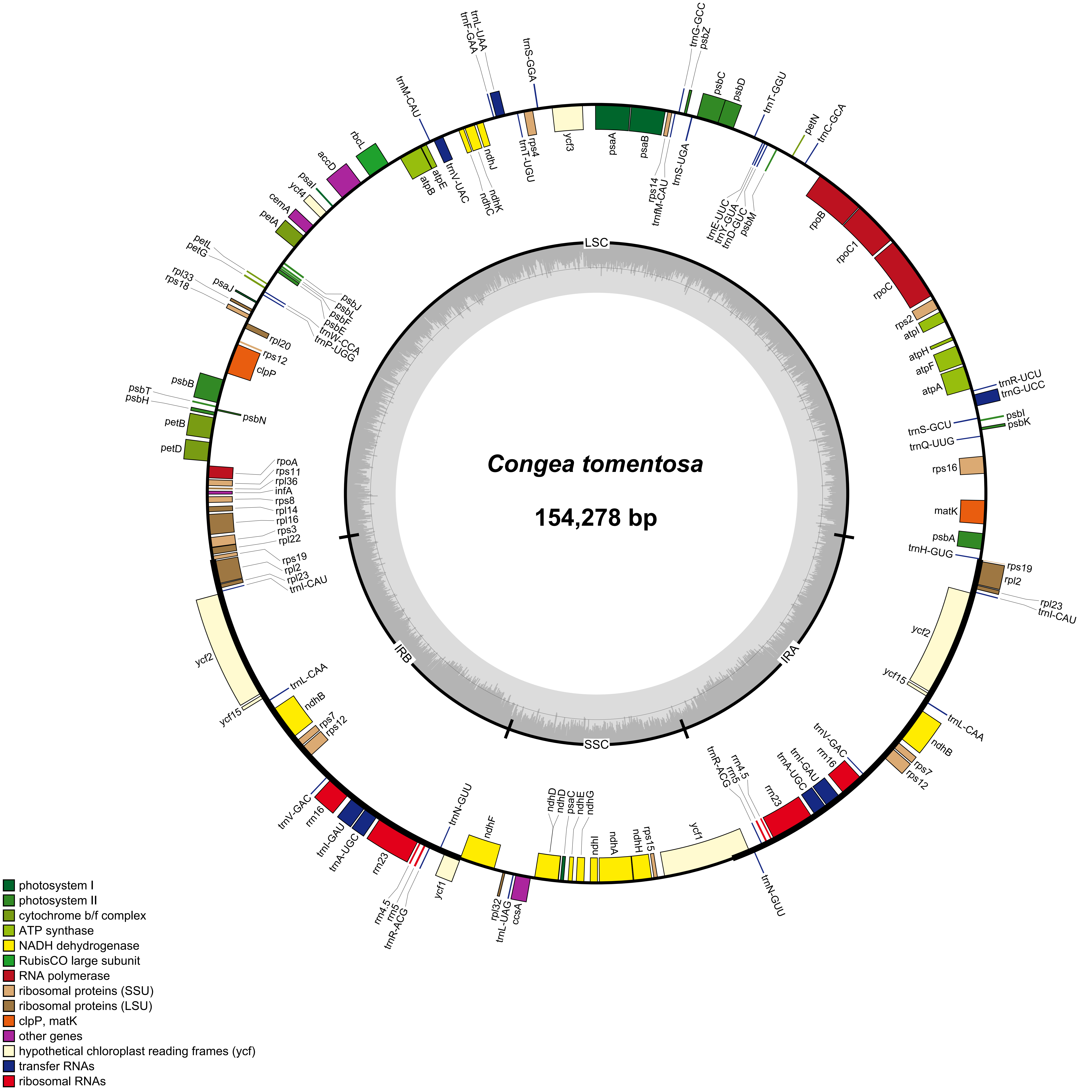
Congea tomentosa
154,278 bp
LSC
SSC
IRA
IRB
trnL-UAA
trnF-GAA
trnS-GGA
trnM-CAU
trnG-GCC
psbZ
psbC
psbD
trnT-GGU
petN
trnC-GCA
rbcL
accD
psaI
ycf4
cemA
petA
atpE
atpB
trnV-UAC
ndhJ
ndhK
ndhC
trnT-UGU
rps4
ycf3
psaA
psaB
rps14
trnfM-CAU
trnS-UGA
trnE-UUC
trnY-GUA
trnD-GUC
psbM
rpoB
rpoC1
rpoC
rps2
atpI
atpH
atpF
atpA
trnR-UCU
trnG-UCC
psbI
psbK
trnS-GCU
trnQ-UUG
rps16
matK
psbA
trnH-GUG
rps19
rpl2
rpl23
trnI-CAU
ycf2
ycf15
trnL-CAA
ndhB
rps7
rps12
trnV-GAC
rrn16
trnI-GAU
trnA-UGC
rrn23
rrn4.5
rrn5
trnR-ACG
trnN-GUU
ycf1
rps15
ndhH
ndhA
ndhI
ndhG
ndhE
psaC
ndhD
ndhF
rpl32
trnL-UAG
ccsA
petL
petG
psaJ
rpl33
rps18
psbJ
psbL
psbF
psbE
trnW-CCA
trnP-UGG
rpl20
rps12
clpP
psbB
psbT
psbH
psbN
petB
petD
rpoA
rps11
rpl36
infA
rps8
rpl14
rpl16
rps3
rpl22
photosystem I
photosystem II
cytochrome b/f complex
ATP synthase
NADH dehydrogenase
RubisCO large subunit
RNA polymerase
ribosomal proteins (SSU)
ribosomal proteins (LSU)
clpP, matK
other genes
hypothetical chloroplast reading frames (ycf)
transfer RNAs
ribosomal RNAs

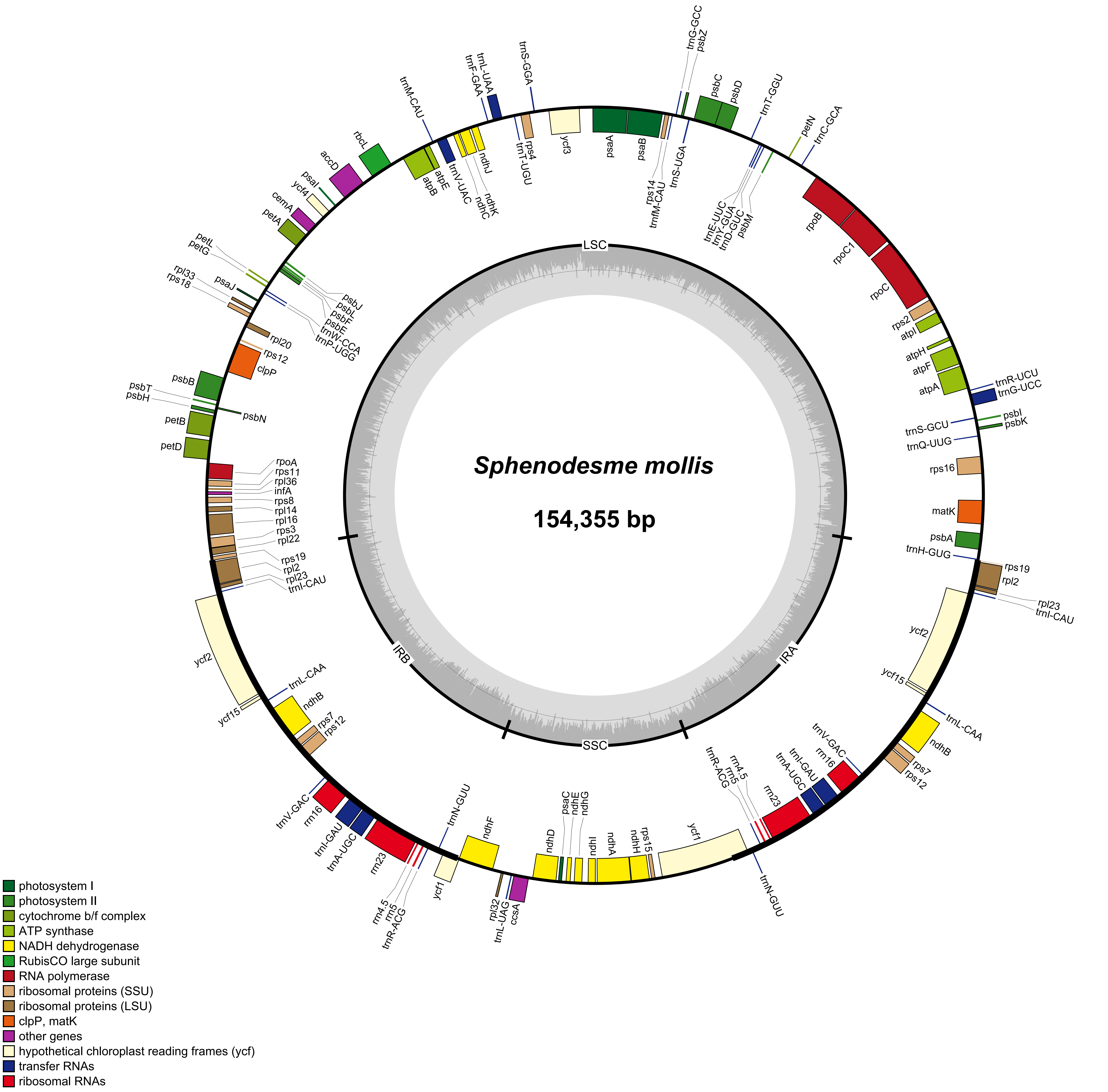
Sphenodesme mollis
154,355 bp
LSC
IRA
SSC
IRB
trnG-GCC
psbZ
psbC
psbD
trnT-GGU
petN
trnC-GCA
rpoB
rpoC1
rpoC
rps2
atpI
atpH
atpF
atpA
trnR-UCU
trnG-UCC
psbI
psbK
trnS-GCU
trnQ-UUG
rps16
matK
psbA
trnH-GUG
rps19
rpl2
rpl23
trnI-CAU
ycf2
ycf15
trnL-CAA
ndhB
rps7
rps12
trnV-GAC
rrn16
trnI-GAU
trnA-UGC
rrn23
rrn4.5
rrn5
trnR-ACG
trnN-GUU
ycf1
rps15
ndhH
ndhA
ndhI
ndhG
ndhE
psaC
ndhD
ccsA
trnL-UAG
rpl32
ndhF
rpoA
rps11
rpl36
infA
rps8
rpl14
rpl16
rps3
rpl22
petD
petB
psbH
psbT
psbB
psbN
clpP
rpl20
rpl33
rps18
psaJ
petL
petG
psbJ
psbL
psbF
psbE
trnW-CCA
trnP-UGG
petA
cemA
ycf4
psaI
accD
rbcL
atpB
atpE
trnM-CAU
trnV-UAC
ndhC
ndhK
ndhJ
trnF-GAA
trnL-UAA
trnS-GGA
trnT-UGU
rps4
ycf3
psaA
psaB
rps14
trnfM-CAU
trnS-UGA
trnE-UUC
trnY-GUA
trnD-GUC
psbM
photosystem I
photosystem II
cytochrome b/f complex
ATP synthase
NADH dehydrogenase
RubisCO large subunit
RNA polymerase
ribosomal proteins (SSU)
ribosomal proteins (LSU)
clpP, matK
other genes
hypothetical chloroplast reading frames (ycf)
transfer RNAs
ribosomal RNAs

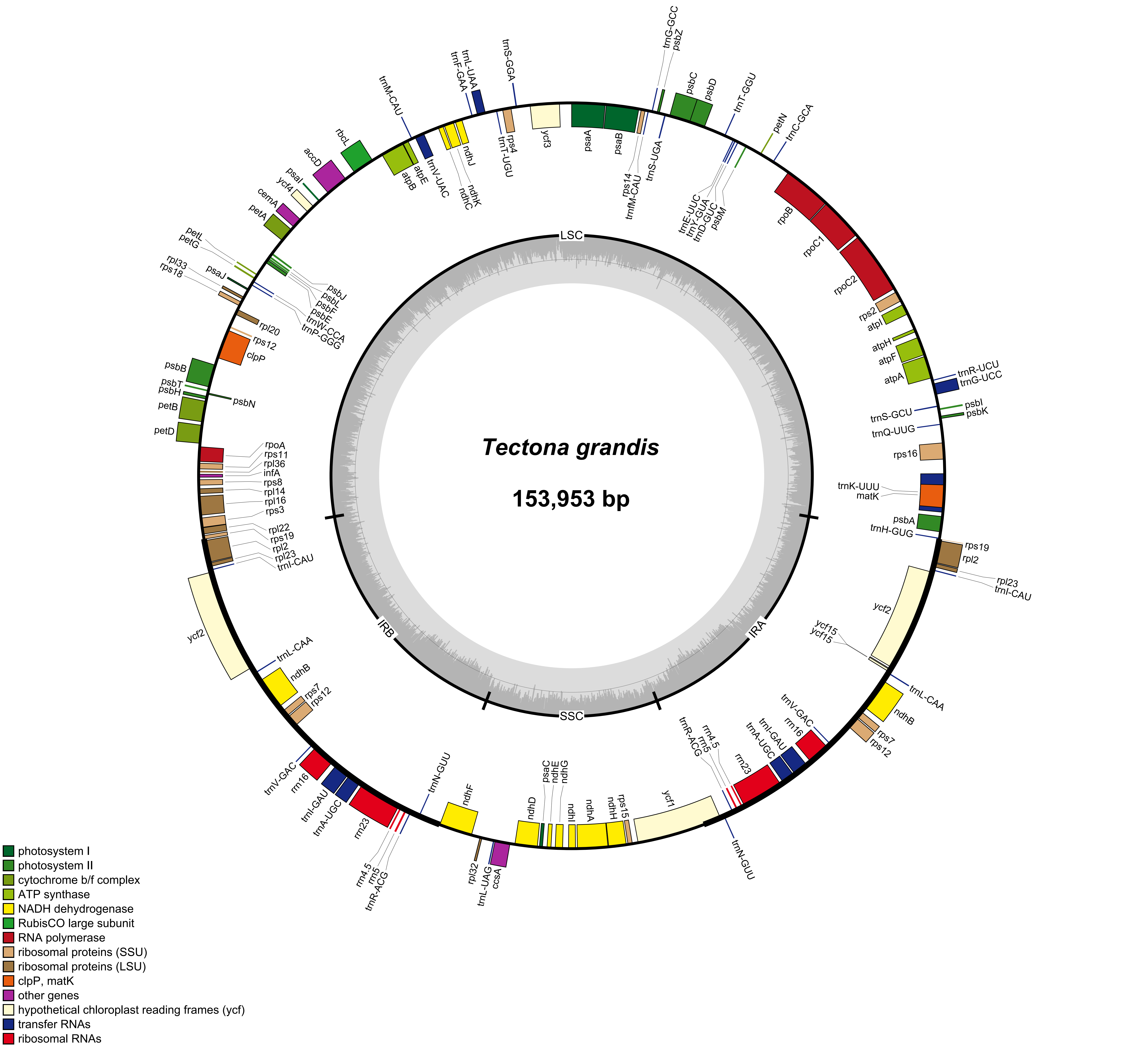

Tectona grandis
153,953 bp
LSC
IRA
SSC
IRB
psbA
trnH-GUG
trnK-UUU
matK
rps16
trnQ-UUG
trnS-GCU
psbI
psbK
trnG-UCC
trnR-UCU
atpA
atpF
atpH
atpI
rps2
rpoC2
rpoC1
rpoB
trnC-GCA
petN
psbM
trnD-GUC
trnY-GUA
trnE-UUC
trnT-GGU
psbD
psbC
trnS-UGA
psbZ
trnG-GCC
trnfM-CAU
rps14
psaB
psaA
ycf3
trnS-GGA
rps4
trnT-UGU
trnL-UAA
trnF-GAA
ndhJ
ndhK
ndhC
trnV-UAC
trnM-CAU
atpE
atpB
rbcL
accD
psaI
ycf4
cemA
petA
psbJ
psbL
psbF
psbE
petL
petG
trnW-CCA
trnP-GGG
psaJ
rpl33
rps18
rpl20
rps12
clpP
psbB
psbT
psbH
psbN
petB
petD
rpoA
rps11
rpl36
infA
rps8
rpl14
rpl16
rps3
rpl22
rps19
rpl2
rpl23
trnI-CAU
ycf2
ycf15
trnL-CAA
ndhB
rps7
trnV-GAC
rrn16
trnI-GAU
trnA-UGC
rrn23
rrn4.5
rrn5
trnR-ACG
trnN-GUU
ndhF
rpl32
trnL-UAG
ccsA
ndhD
psaC
ndhE
ndhG
ndhI
ndhA
ndhH
rps15
ycf1
photosystem I
photosystem II
cytochrome b/f complex
ATP synthase
NADH dehydrogenase
RubisCO large subunit
RNA polymerase
ribosomal proteins (SSU)
ribosomal proteins (LSU)
clpP, matK
other genes
hypothetical chloroplast reading frames (ycf)
transfer RNAs
ribosomal RNAs

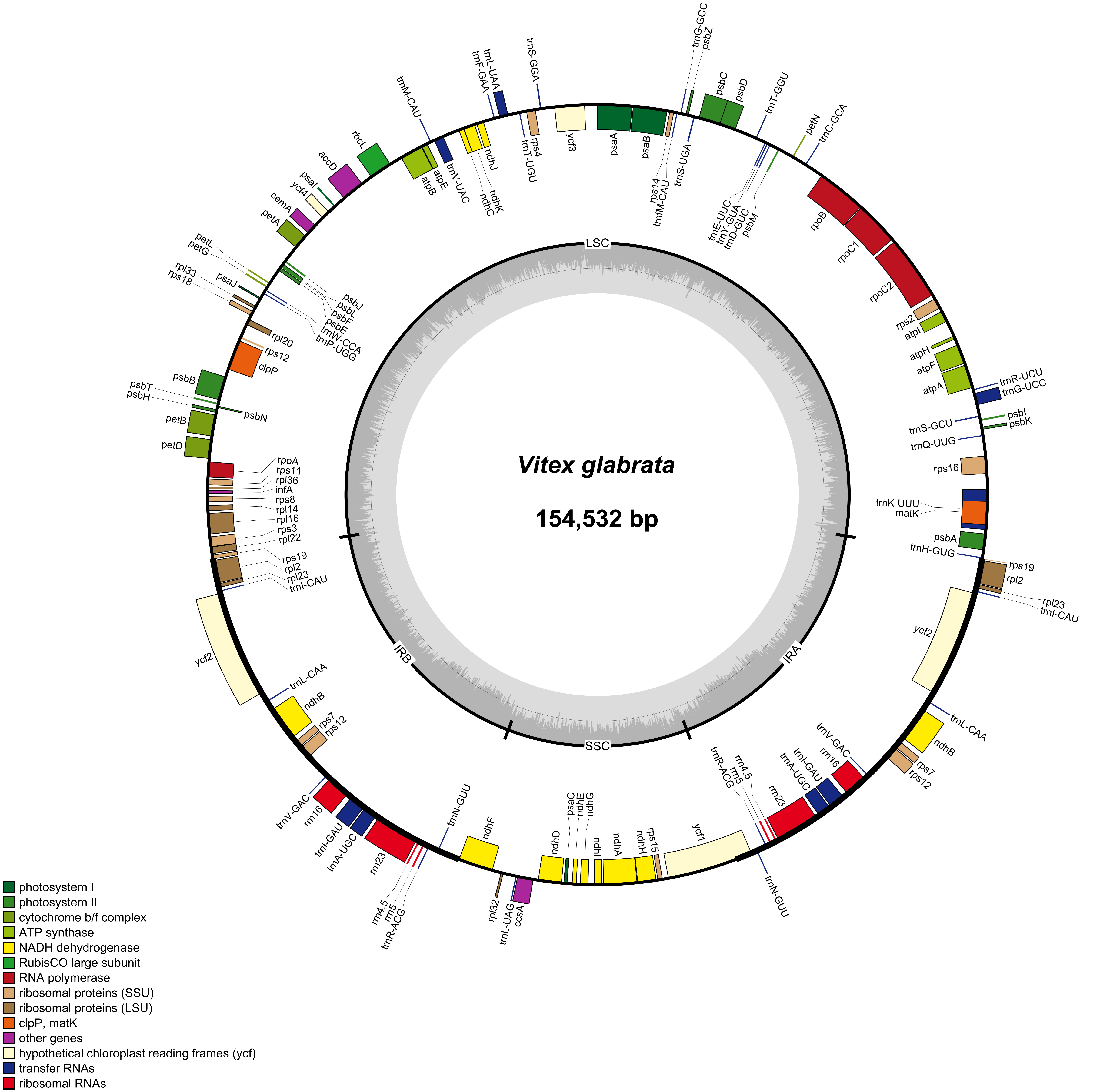

Vitex glabrata
154,532 bp
LSC
IRA
SSC
IRB
trnG-GCC
psbZ
psbC
psbD
trnT-GGU
petN
trnC-GCA
trnS-UGA
trnfM-CAU
rps14
psaB
psaA
ycf3
trnS-GGA
rps4
trnT-UGU
trnL-UAA
trnF-GAA
ndhJ
ndhK
ndhC
trnV-UAC
trnM-CAU
atpE
atpB
rbcL
accD
psaI
ycf4
cemA
petA
petL
petG
psaJ
rpl33
rps18
psbJ
psbL
psbF
psbE
trnW-CCA
trnP-UGG
rpl20
rps12
clpP
psbB
psbT
psbH
psbN
petB
petD
rpoA
rps11
rpl36
infA
rps8
rpl14
rpl16
rps3
rpl22
rps19
rpl2
rpl23
trnI-CAU
ycf2
trnL-CAA
ndhB
rps7
trnV-GAC
rrn16
trnI-GAU
trnA-UGC
rrn23
rrn4.5
rrn5
trnR-ACG
trnN-GUU
ndhF
rpl32
trnL-UAG
ccsA
ndhD
psaC
ndhE
ndhG
ndhI
ndhA
ndhH
rps15
ycf1
trnE-UUC
trnY-GUA
trnD-GUC
psbM
rpoB
rpoC1
rpoC2
rps2
atpI
atpH
atpF
atpA
trnR-UCU
trnG-UCC
psbI
psbK
trnS-GCU
trnQ-UUG
rps16
trnK-UUU
matK
psbA
trnH-GUG
photosystem I
photosystem II
cytochrome b/f complex
ATP synthase
NADH dehydrogenase
RubisCO large subunit
RNA polymerase
ribosomal proteins (SSU)
ribosomal proteins (LSU)
clpP, matK
other genes
hypothetical chloroplast reading frames (ycf)
transfer RNAs
ribosomal RNAs

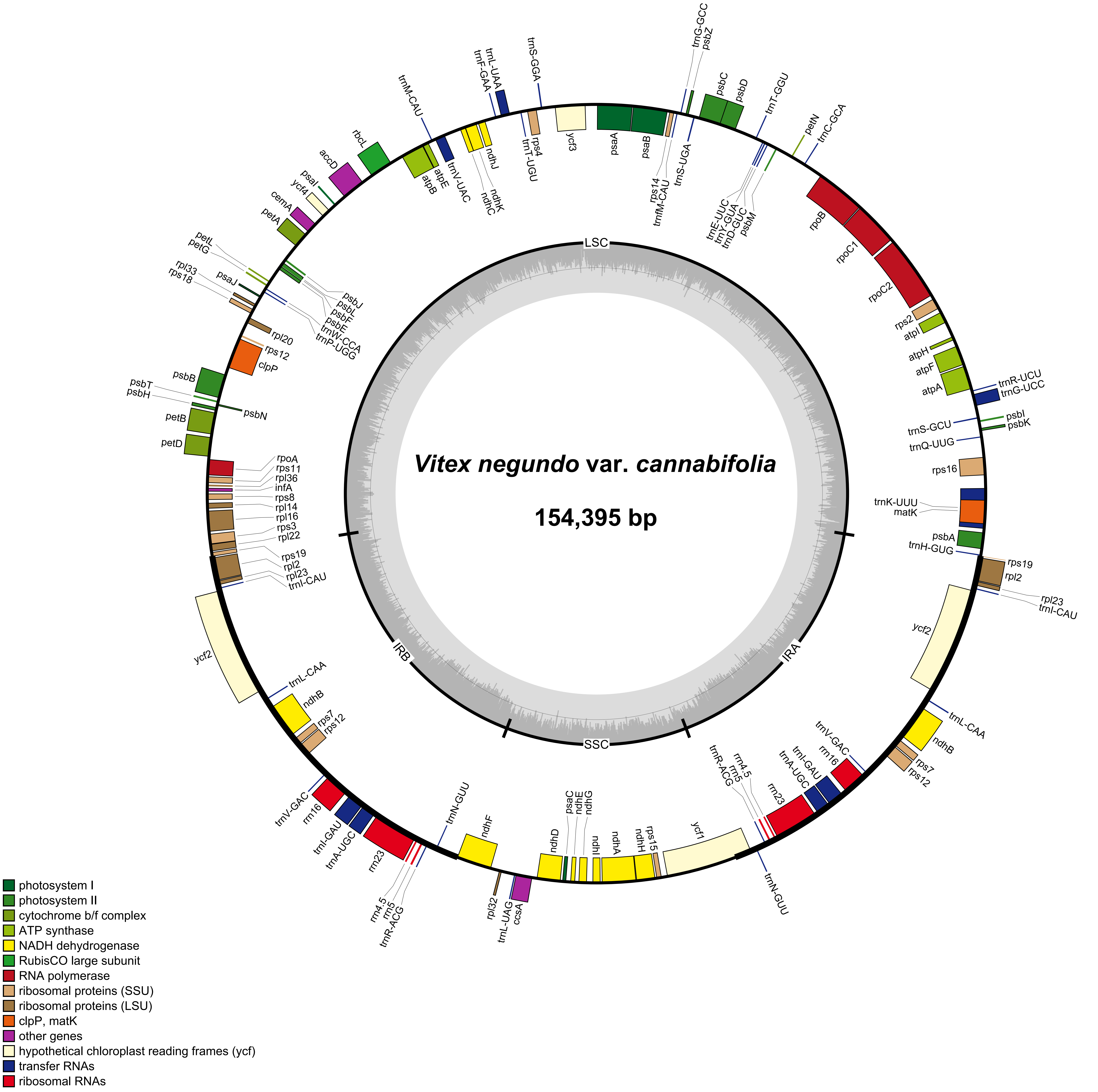
Vitex negundo var. cannabifolia
154,395 bp
LSC
SSC
IRA
IRB
trnG-GCC
psbZ
psbC
psbD
trnT-GGU
petN
trnC-GCA
rpoB
rpoC1
rpoC2
rps2
atpI
atpH
atpF
atpA
trnR-UCU
trnG-UCC
psbI
psbK
trnS-GCU
trnQ-UUG
rps16
trnK-UUU
matK
psbA
trnH-GUG
rps19
rpl2
rpl23
trnI-CAU
ycf2
trnL-CAA
ndhB
rps7
rps12
trnV-GAC
rrn16
trnI-GAU
trnA-UGC
rrn23
rrn4.5
rrn5
trnR-ACG
trnN-GUU
ycf1
rps15
ndhH
ndhA
ndhI
ndhG
ndhE
psaC
ndhD
ccsA
trnL-UAG
rpl32
ndhF
rpoA
rps11
rpl36
infA
rps8
rpl14
rpl16
rps3
rpl22
petD
petB
psbH
psbT
psbN
psbB
clpP
rpl20
rpl33
rps18
psaJ
trnP-UGG
trnW-CCA
psbE
psbF
psbL
psbJ
petL
petG
petA
cemA
ycf4
psaI
accD
rbcL
atpB
atpE
trnM-CAU
trnV-UAC
ndhC
ndhK
ndhJ
trnF-GAA
trnL-UAA
trnT-UGU
rps4
trnS-GGA
ycf3
psaA
psaB
rps14
trnfM-CAU
trnS-UGA
trnE-UUC
trnY-GUA
trnD-GUC
psbM
photosystem I
photosystem II
cytochrome b/f complex
ATP synthase
NADH dehydrogenase
RubisCO large subunit
RNA polymerase
ribosomal proteins (SSU)
ribosomal proteins (LSU)
clpP, matK
other genes
hypothetical chloroplast reading frames (ycf)
transfer RNAs
ribosomal RNAs

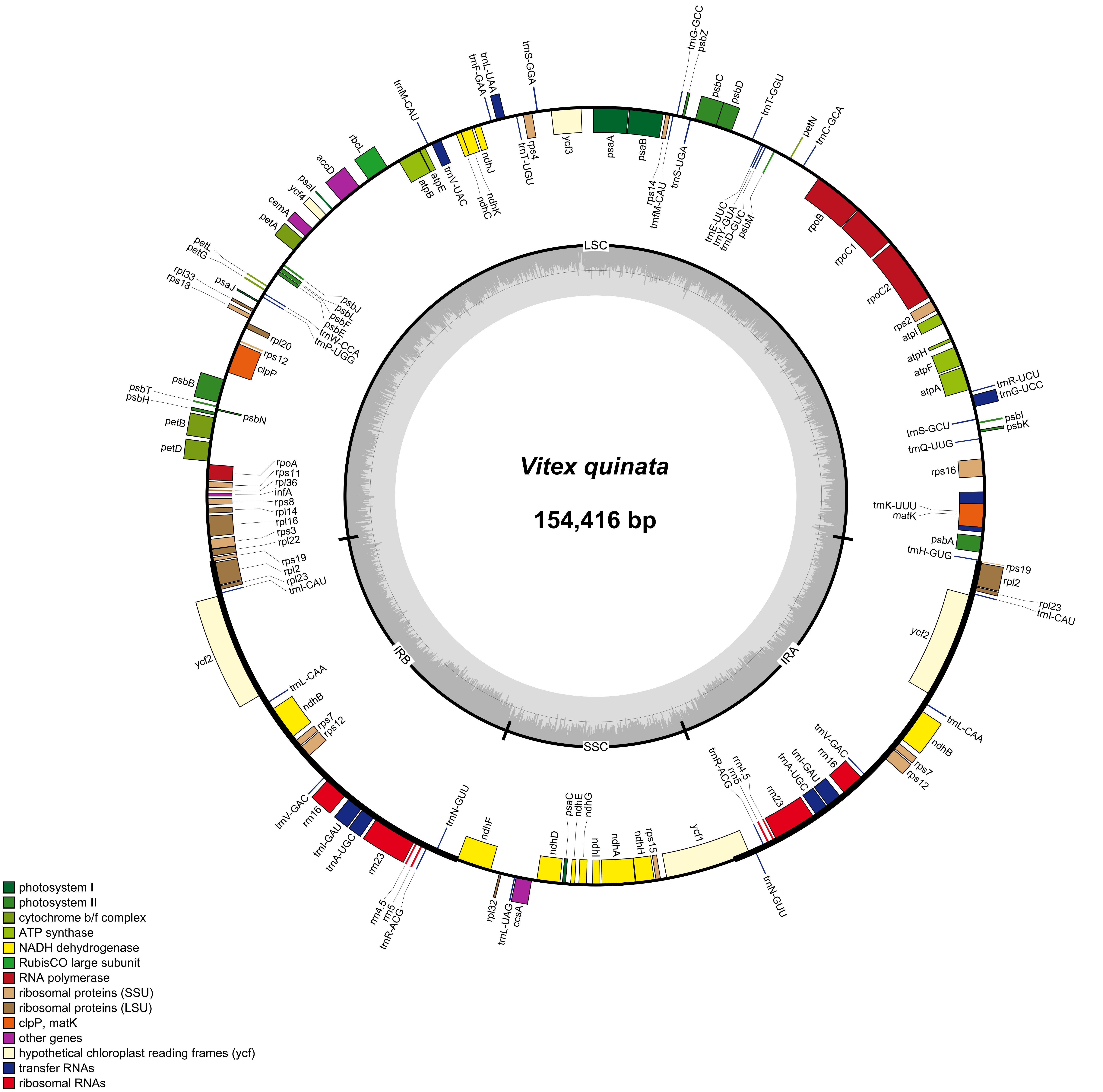

Vitex quinata
154,416 bp
LSC
IRA
SSC
IRB
trnG-GCC
psbZ
psbC
psbD
trnT-GGU
petN
trnC-GCA
rpoB
rpoC1
rpoC2
rps2
atpI
atpH
atpF
atpA
trnR-UCU
trnG-UCC
psbI
psbK
trnS-GCU
trnQ-UUG
rps16
trnK-UUU
matK
psbA
trnH-GUG
rps19
rpl2
rpl23
trnI-CAU
ycf2
trnL-CAA
ndhB
rps7
rps12
trnV-GAC
rrn16
trnI-GAU
trnA-UGC
rrn23
rrn4.5
rrn5
trnR-ACG
trnN-GUU
ycf1
rps15
ndhH
ndhA
ndhI
ndhG
ndhE
psaC
ndhD
ccsA
trnL-UAG
rpl32
ndhF
trnS-GGA
trnL-UAA
trnF-GAA
trnM-CAU
rbcL
accD
psaI
ycf4
cemA
petA
petL
petG
psaJ
rpl33
rps18
psbJ
psbL
psbF
psbE
trnW-CCA
trnP-UGG
rpl20
clpP
psbB
psbT
psbH
psbN
petB
petD
rpoA
rps11
rpl36
infA
rps8
rpl14
rpl16
rps3
rpl22
atpE
atpB
trnV-UAC
ndhJ
ndhK
ndhC
trnT-UGU
rps4
ycf3
psaA
psaB
rps14
trnfM-CAU
trnS-UGA
trnE-UUC
trnY-GUA
trnD-GUC
psbM
photosystem I
photosystem II
cytochrome b/f complex
ATP synthase
NADH dehydrogenase
RubisCO large subunit
RNA polymerase
ribosomal proteins (SSU)
ribosomal proteins (LSU)
clpP, matK
other genes
hypothetical chloroplast reading frames (ycf)
transfer RNAs
ribosomal RNAs

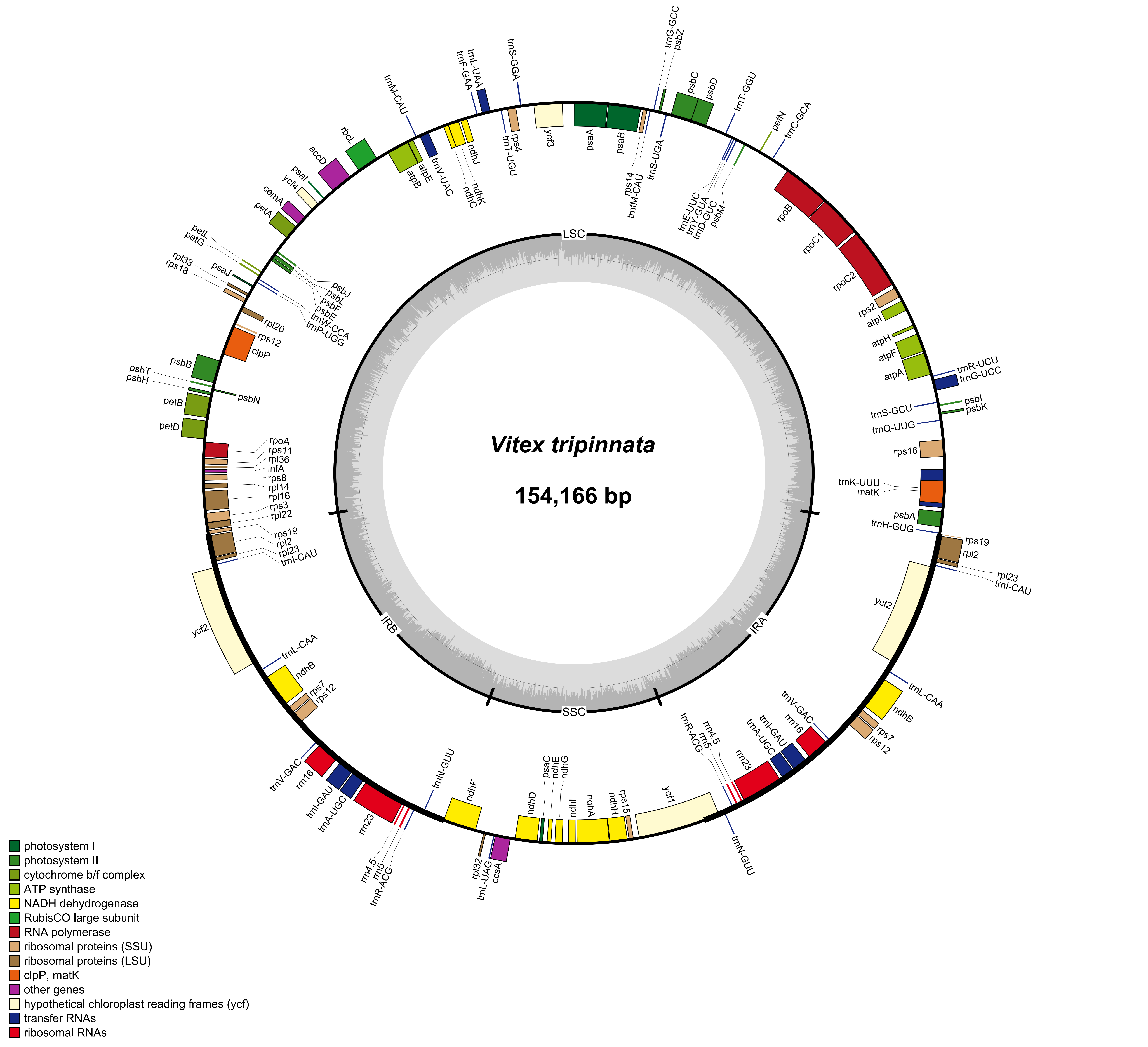

Vitex tripinnata
154,166 bp
LSC
IRA
SSC
IRB
trnG-GCC
psbZ
psbC
psbD
trnT-GGU
petN
trnC-GCA
rpoB
rpoC1
rpoC2
rps2
atpI
atpH
atpF
atpA
trnR-UCU
trnG-UCC
psbI
psbK
trnS-GCU
trnQ-UUG
rps16
trnK-UUU
matK
psbA
trnH-GUG
rps19
rpl2
rpl23
trnI-CAU
ycf2
trnL-CAA
ndhB
rps7
rps12
trnV-GAC
rrn16
trnI-GAU
trnA-UGC
rrn23
rrn4.5
rrn5
trnR-ACG
trnN-GUU
ycf1
rps15
ndhH
ndhA
ndhI
ndhG
ndhE
psaC
ndhD
ccsA
trnL-UAG
rpl32
ndhF
rpoA
rps11
rpl36
infA
rps8
rpl14
rpl16
rps3
rpl22
petD
petB
psbH
psbT
psbB
psbN
clpP
rps12
rpl20
rps18
rpl33
psaJ
petG
petL
psbJ
psbL
psbF
psbE
trnW-CCA
trnP-UGG
petA
cemA
ycf4
psaI
accD
rbcL
atpB
atpE
trnM-CAU
trnV-UAC
ndhC
ndhK
ndhJ
trnF-GAA
trnL-UAA
trnT-UGU
rps4
trnS-GGA
ycf3
psaA
psaB
rps14
trnfM-CAU
trnS-UGA
trnE-UUC
trnY-GUA
trnD-GUC
psbM
photosystem I
photosystem II
cytochrome b/f complex
ATP synthase
NADH dehydrogenase
RubisCO large subunit
RNA polymerase
ribosomal proteins (SSU)
ribosomal proteins (LSU)
clpP, matK
other genes
hypothetical chloroplast reading frames (ycf)
transfer RNAs
ribosomal RNAs

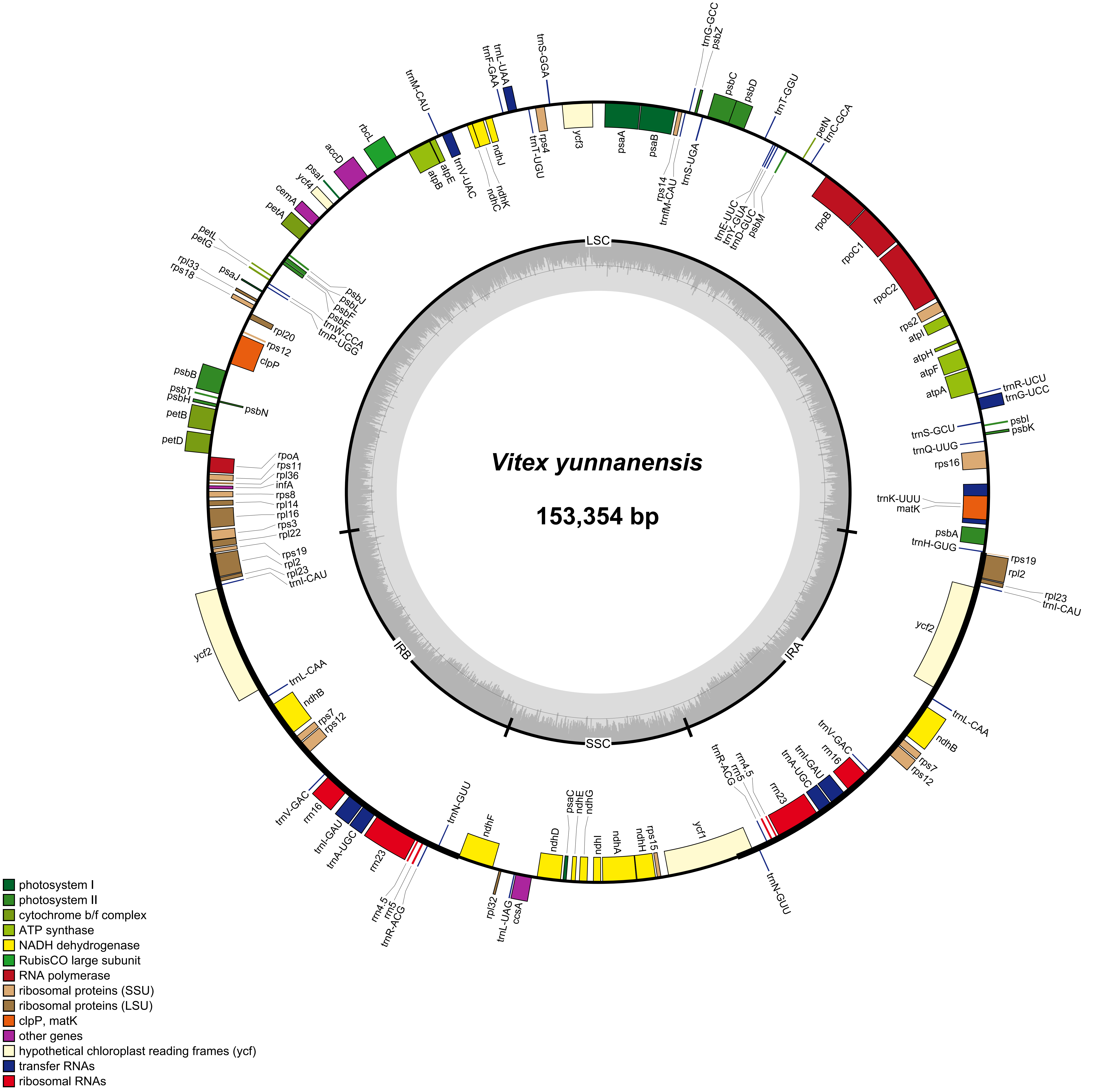

Vitex yunnanensis
153,354 bp
LSC
IRA
IRB
SSC
trnG-GCC
psbZ
psbC
psbD
trnT-GGU
petN
trnC-GCA
rpoB
rpoC1
rpoC2
rps2
atpI
atpH
atpF
atpA
trnR-UCU
trnG-UCC
trnS-GCU
psbI
psbK
trnQ-UUG
rps16
trnK-UUU
matK
psbA
trnH-GUG
rps19
rpl2
rpl23
trnI-CAU
ycf2
trnL-CAA
ndhB
rps7
rps12
trnV-GAC
rrn16
trnI-GAU
trnA-UGC
rrn23
rrn4.5
rrn5
trnR-ACG
trnN-GUU
ycf1
rps15
ndhH
ndhA
ndhI
ndhG
ndhE
psaC
ndhD
ccsA
trnL-UAG
rpl32
ndhF
trnS-GGA
trnL-UAA
trnF-GAA
trnM-CAU
rbcL
accD
psaI
ycf4
cemA
petA
petL
petG
rpl33
rps18
psaJ
psbJ
psbL
psbF
psbE
trnW-CCA
trnP-UGG
rpl20
clpP
psbB
psbT
psbH
psbN
petB
petD
rpoA
rps11
rpl36
infA
rps8
rpl14
rpl16
rps3
rpl22
ycf3
psaA
psaB
rps14
trnfM-CAU
trnS-UGA
rps4
trnT-UGU
ndhJ
ndhK
ndhC
trnV-UAC
atpE
atpB
trnE-UUC
trnY-GUA
trnD-GUC
psbM
photosystem I
photosystem II
cytochrome b/f complex
ATP synthase
NADH dehydrogenase
RubisCO large subunit
RNA polymerase
ribosomal proteins (SSU)
ribosomal proteins (LSU)
clpP, matK
other genes
hypothetical chloroplast reading frames (ycf)
transfer RNAs
ribosomal RNAs

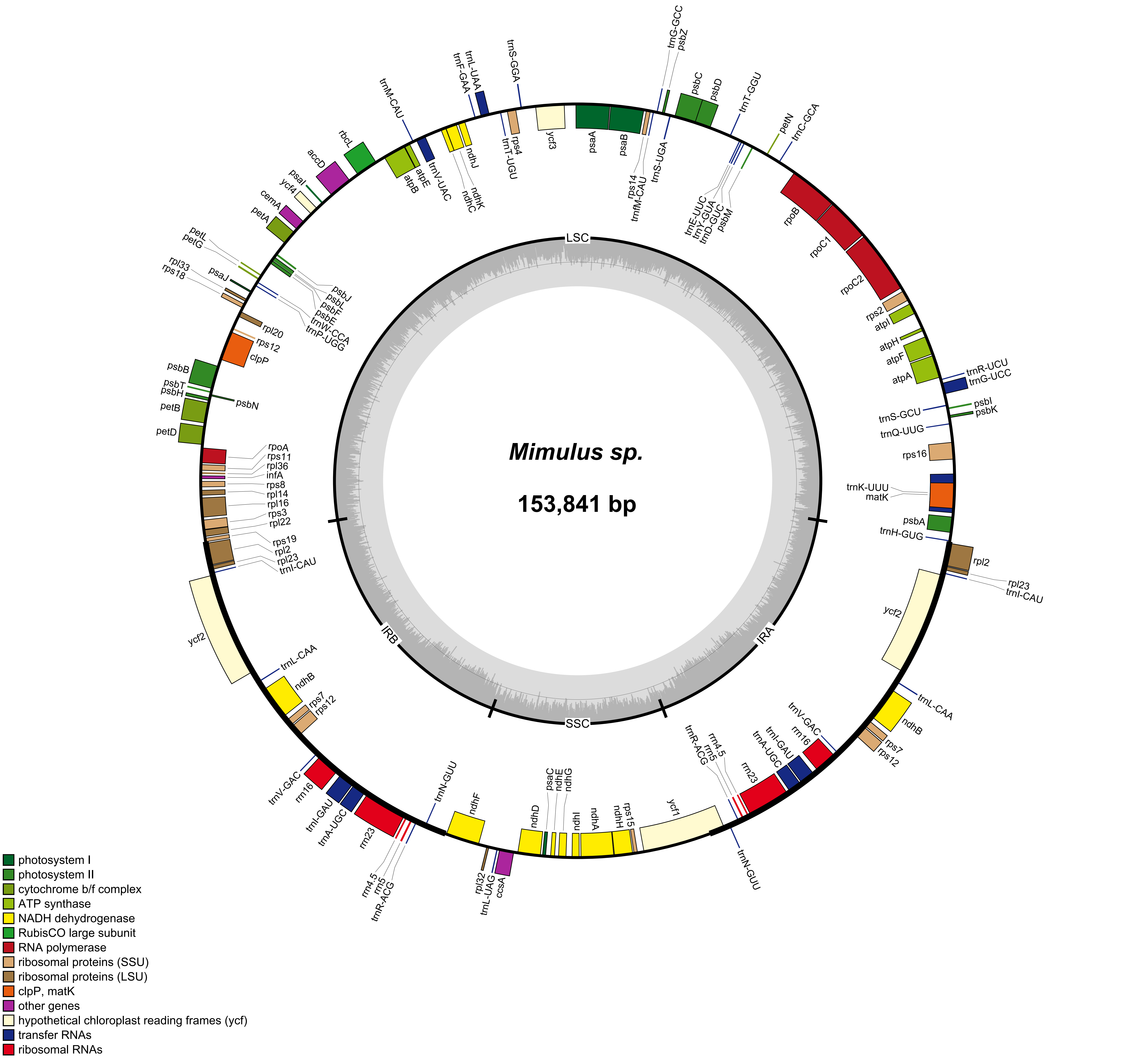

Mimulus sp.
153,841 bp
LSC
IRA
SSC
IRB
trnG-GCC
psbZ
psbC
psbD
trnT-GGU
petN
trnC-GCA
rpoB
rpoC1
rpoC2
rps2
atpI
atpH
atpF
atpA
trnR-UCU
trnG-UCC
psbI
psbK
trnS-GCU
trnQ-UUG
rps16
trnK-UUU
matK
psbA
trnH-GUG
rpl2
rpl23
trnI-CAU
ycf2
trnL-CAA
ndhB
rps7
rps12
trnV-GAC
rrn16
trnI-GAU
trnA-UGC
rrn23
rrn4.5
rrn5
trnR-ACG
trnN-GUU
ycf1
rps15
ndhH
ndhA
ndhI
ndhG
ndhE
psaC
ndhD
ccsA
trnL-UAG
rpl32
ndhF
trnS-GGA
rps4
trnT-UGU
ycf3
psaA
psaB
rps14
trnfM-CAU
trnS-UGA
trnE-UUC
trnY-GUA
trnD-GUC
psbM
trnL-UAA
trnF-GAA
ndhJ
ndhK
ndhC
trnV-UAC
trnM-CAU
atpE
atpB
rbcL
accD
psaI
ycf4
cemA
petA
petL
petG
psbJ
psbL
psbF
psbE
psaJ
rpl33
rps18
trnW-CCA
trnP-UGG
rpl20
rps12
clpP
psbB
psbT
psbH
psbN
petB
petD
rpoA
rps11
rpl36
infA
rps8
rpl14
rpl16
rps3
rpl22
rps19
rpl2
rpl23
trnI-CAU
photosystem I
photosystem II
cytochrome b/f complex
ATP synthase
NADH dehydrogenase
RubisCO large subunit
RNA polymerase
ribosomal proteins (SSU)
ribosomal proteins (LSU)
clpP, matK
other genes
hypothetical chloroplast reading frames (ycf)
transfer RNAs
ribosomal RNAs
