# Supplementary figures and images for: An updated tribal classification of Lamiaceae based on plastome phylogenomics
Source: BMC Biol. 2021 Jan 8;19:2. doi: 10.1186/s12915-020-00931-z (PMC7796571; doi:10.1186/s12915-020-00931-z)

A

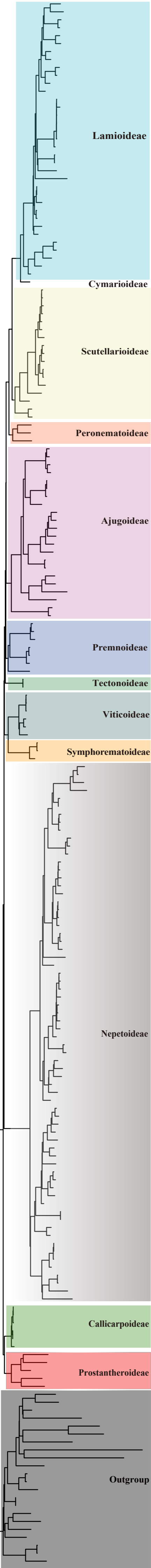

B

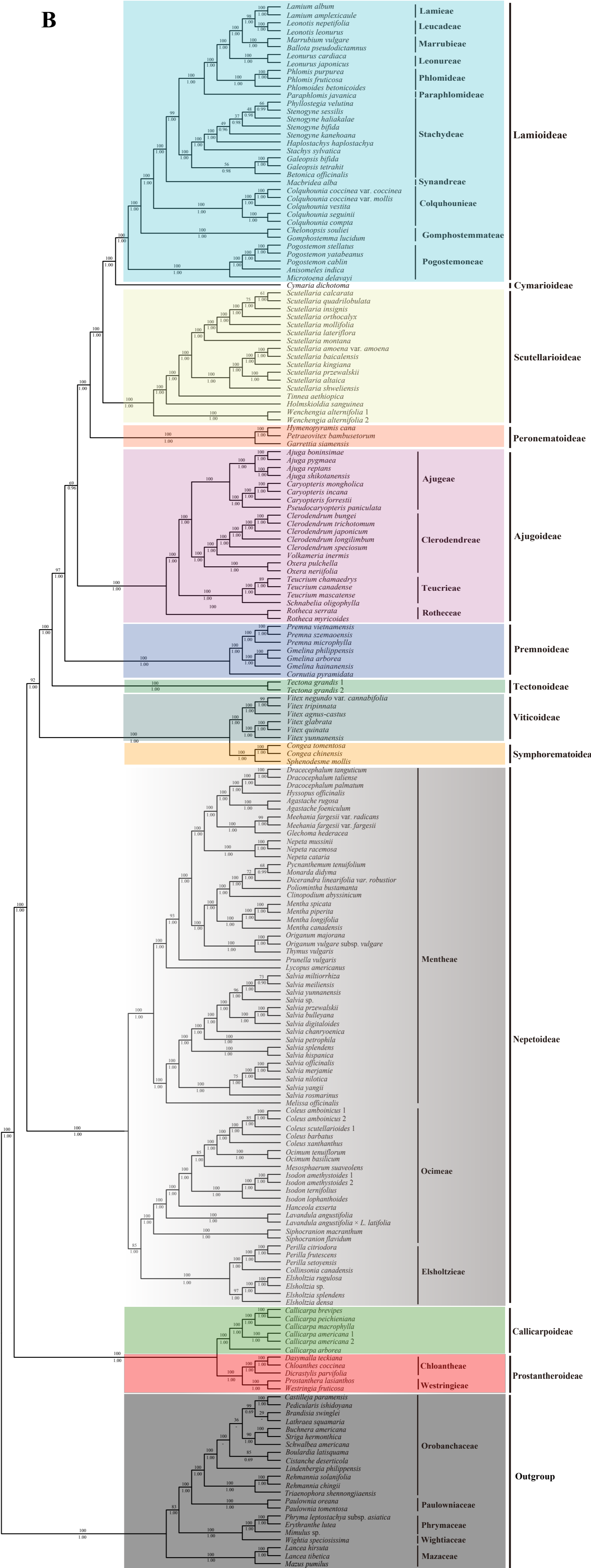

Supplement: Supplementary file 4 — Additional file 4: Figure S2. Phylograms inferred from ML analysis of concatenated nucleotide sequences of 79 protein-coding genes (dataset CR). A, phylogram showing branch lengths, where tips names are absent follow the same order as shown in B. Scale bar represents the mean number of nucleotide substitutions per site. B, maximum likelihood bootstrap support values and Bayesian inference posterior probabilities are shown above and below the branches, respectively. [file 12915_2020_931_MOESM4_ESM.pdf]

A

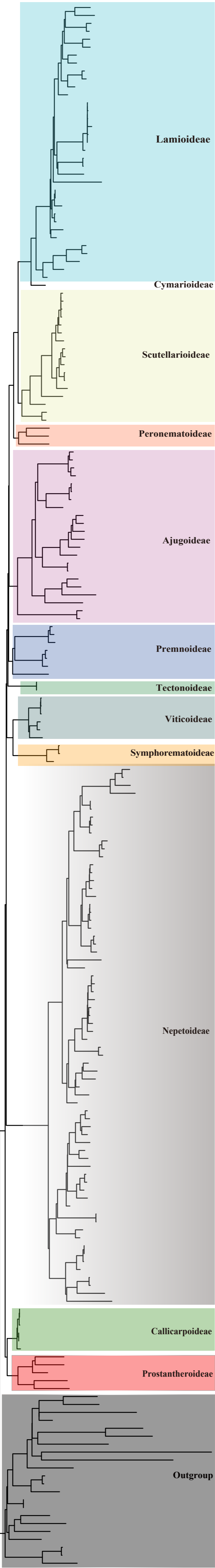

B

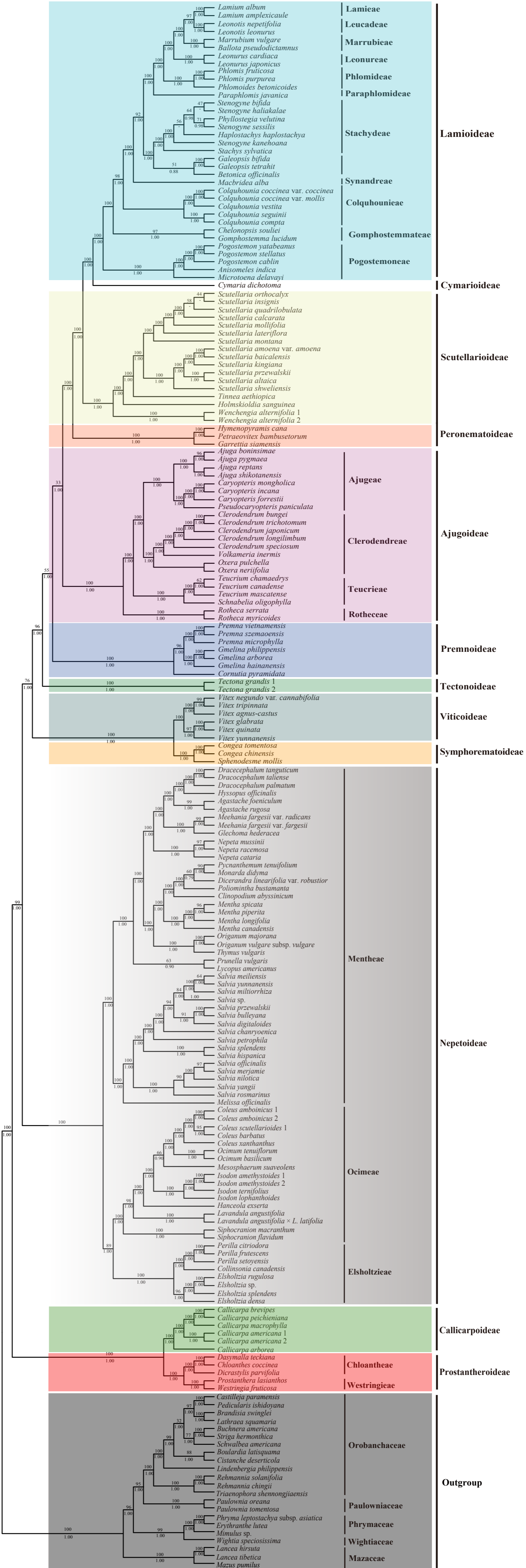

Supplement: Supplementary file 5 — Additional file 5: Figure S3. Phylograms inferred from ML analysis of concatenated nucleotide sequences of the 3rd codon positions (dataset CR3). A, phylogram showing branch lengths, where tip names are absent follow the same order as shown in B. Scale bar represents the mean number of nucleotide substitutions per site. B, maximum likelihood bootstrap support values and Bayesian inference posterior probabilities are shown above and below the branches, respectively. [file 12915_2020_931_MOESM5_ESM.pdf]

A

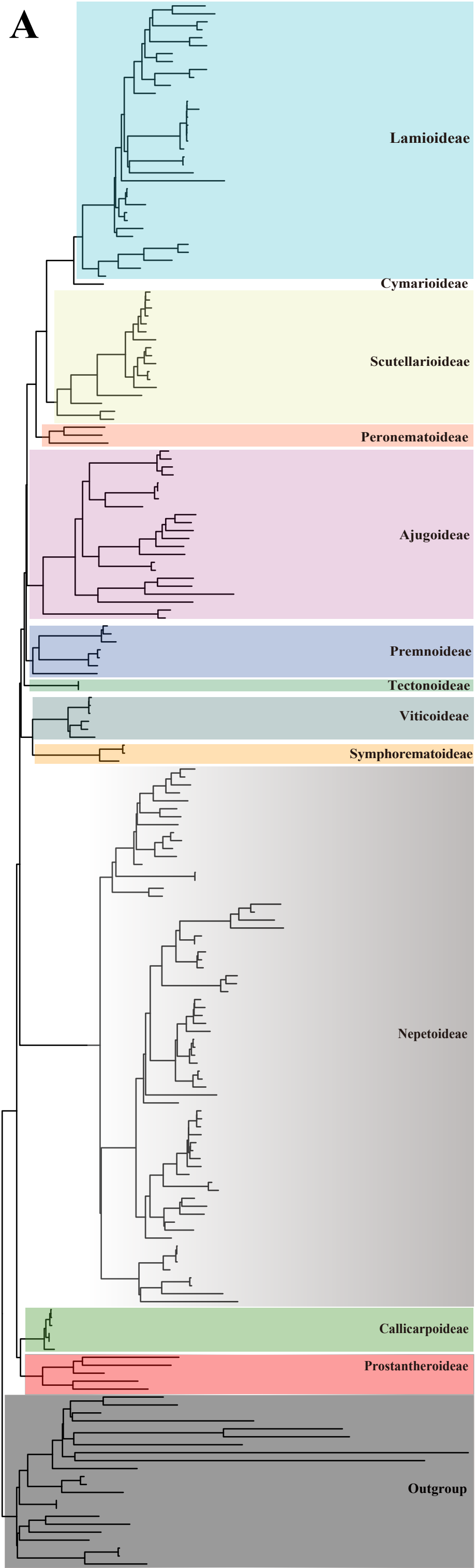

B

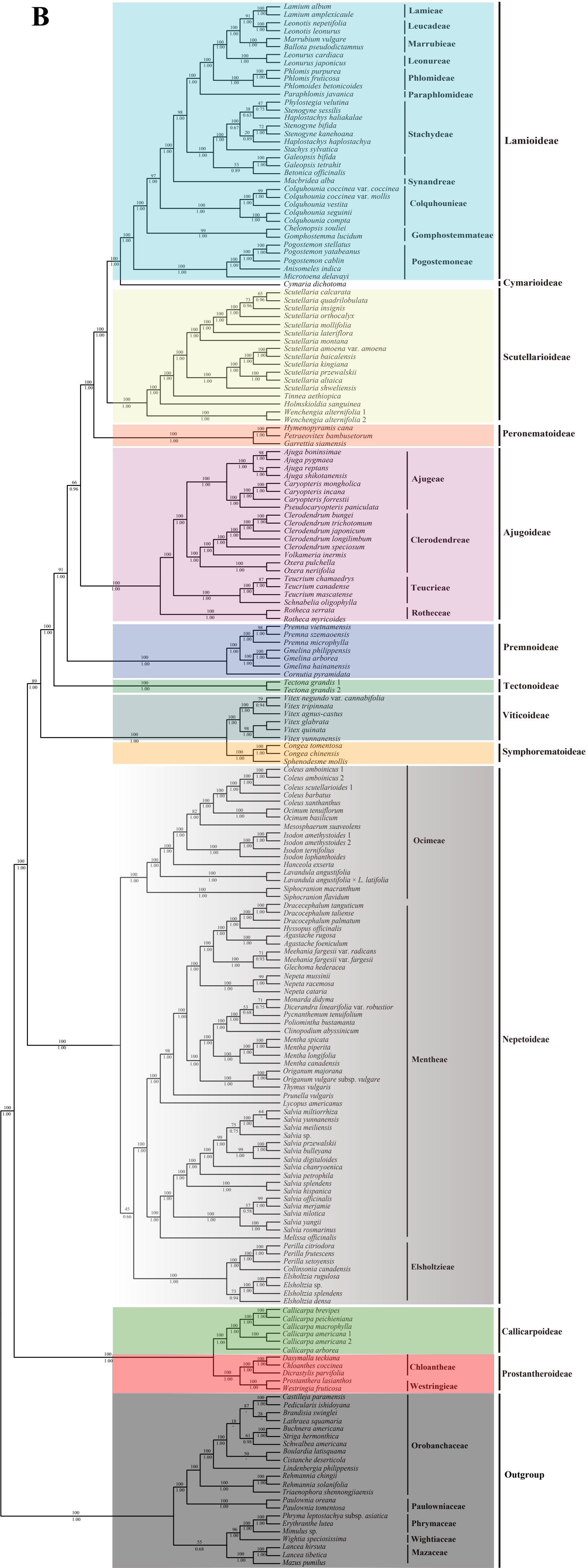

Supplement: Supplementary file 6 — Additional file 6: Figure S4. Phylograms inferred from ML analysis of concatenated nucleotide sequences of the 1st and 2nd codon positions (dataset CR12). A, phylogram showing branch lengths, where tips names are absent follow the same order as shown in B. Scale bar represents the mean number of nucleotide substitutions per site. B, maximum likelihood bootstrap support values and Bayesian inference posterior probabilities are shown above and below the branches, respectively. [file 12915_2020_931_MOESM6_ESM.pdf]

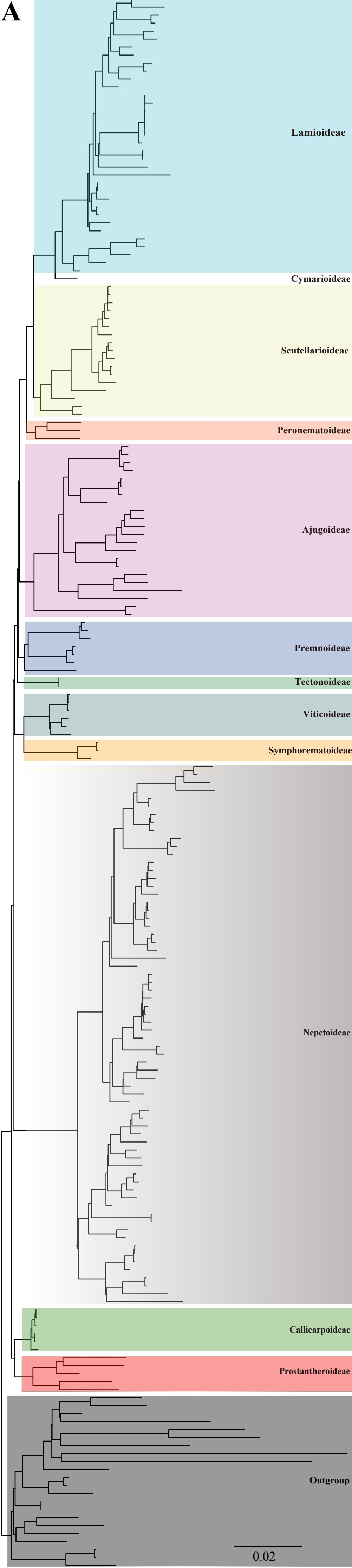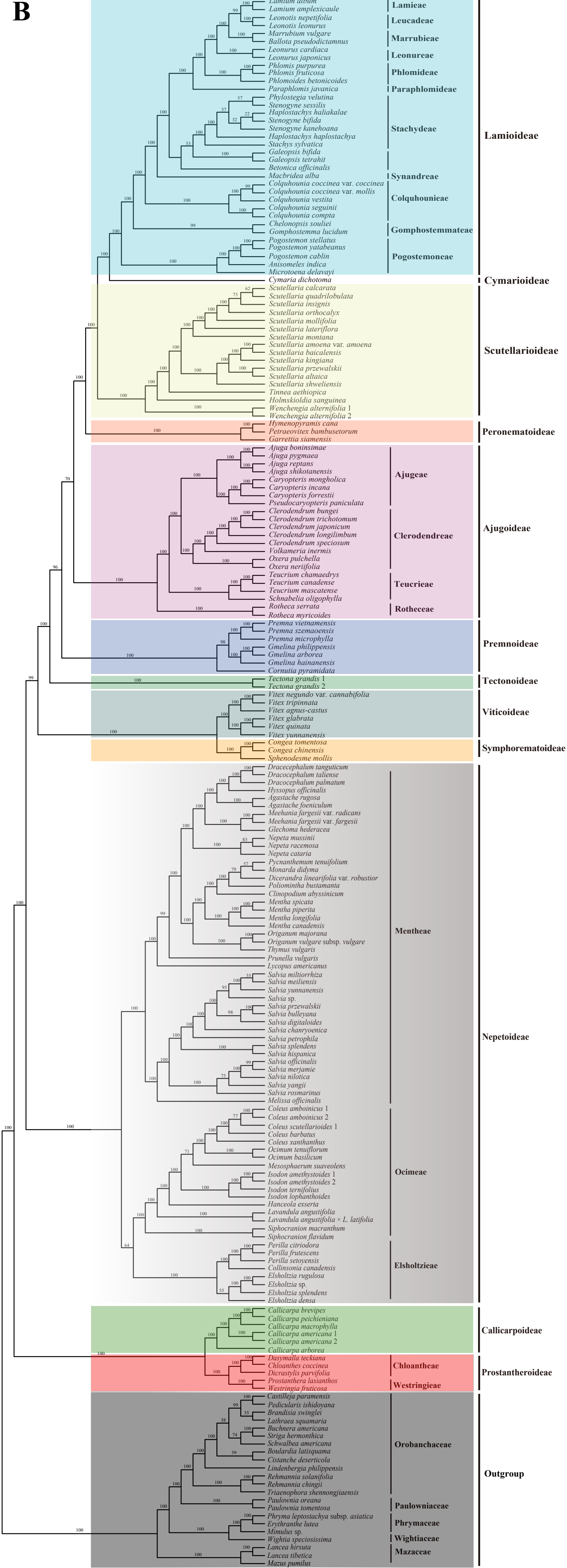

Supplement: Supplementary file 7 — Additional file 7: Figure S5. Phylograms inferred from ML analysis of concatenated nucleotide sequences of the degeneracy nucleotide sequence (dataset dePCS). A, phylogram showing branch lengths, where tip names are absent follow the same order as shown in B. Scale bar represents the mean number of nucleotide substitutions per site. B, maximum likelihood bootstrap support values are shown above the branches. [file 12915_2020_931_MOESM7_ESM.pdf]
